# Molecular basis for depsipeptide HDAC inhibitor combinatorial biosynthesis

**DOI:** 10.1038/s41467-026-74383-4

**Published:** 2026-07-01

**Authors:** Munro Passmore, Xinyun Jian, Xinyi Zhao, Emmanuel L. C. de los Santos, Douglas M. Roberts, Józef R. Lewandowski, Matthew Jenner, Lona M. Alkhalaf, Gregory L. Challis

**Affiliations:** 1 Department of Chemistry, University of Warwick, Coventry, CV4 7AL UK; 2 Warwick Integrative Synthetic Biology Centre, University of Warwick, Coventry, CV4 7AL UK; 3 Department of Biochemistry and Molecular Biology, Biomedicine Discovery Institute, Monash University, Clayton, VIC Australia; 4 ARC Centre of Excellence for Innovations in Protein and Peptide Science, Monash University, Clayton, VIC Australia; 5 Present Address: UCB Pharma, 208 Bath Road, Slough, SL1 3WE UK; 6 Present Address: Erebagen Ltd, CIE Building, Begbroke Science Park, Oxfordshire OX5 1PF UK

**Keywords:** Multienzyme complexes, Natural products, Structural biology, Biophysics, Biochemistry

## Abstract

Polyketides and nonribosomal peptides are important natural product classes with wide-ranging medical and agricultural applications. The analogous enzymatic logic employed by bacterial modular polyketide synthases (PKSs) and nonribosomal peptide synthetases (NRPSs) enables the assembly of hybrid products. One important group of polyketide-nonribosomal peptide hybrids is exemplified by the HDAC-targeting drug romidepsin. This group is assembled by combinatorial biosynthesis involving fusion of a conserved Zn^2+^-binding pharmacophore to a variable peptide-based cap. Here, we use gene proximity searching to identify the FR-901375 biosynthetic gene cluster in *Pseudomonas chlororaphis* subsp. *piscium* DSM 21509. Comparison of the PKS-NRPS encoded by this gene cluster with those assembling related depsipeptide HDAC inhibitors suggests an unusual subunit docking modality enables interaction between the conserved pharmacophore and variable cap biosynthetic machineries. This hypothesis is validated using crosstalk assays, mutagenesis, AlphaFold predictions, and carbene footprinting, providing insight into the evolution of mechanisms for hybrid polyketide-nonribosomal peptide combinatorial biosynthesis.

## Abstract (web summary)

Docking domains ensure subunit interaction fidelity in polyketide and nonribosomal peptide assembly lines. Here, the authors demonstrate how a conserved short linear motif– beta-hairpin docking domain pair enables the evolution of combinatorial histone deacetylase inhibitor biosynthesis.

## Introduction

Polyketides and nonribosomal peptides are families of natural products with diverse applications in medicine (e.g. as antibiotics, anticancer agents, and immunomodulators) and agriculture (e.g. as insecticides, herbicides and fungicides). In bacteria, these metabolite families are typically assembled by PKS and NRPS modular multienzymes, respectively, which condense and modify a series of (alkyl)malonyl, or aminoacyl building blocks^1,2^. The alkyl(malonyl) building blocks employed by PKSs are coenzyme A (CoA) thioester derivatives, which are activated towards condensation. In contrast, NRPSs employ amino acids as substrates, which require activation to enable them to be condensed. Thus, acyltransferase (AT) domains are used by PKSs to transfer an acyl or (alkyl)malonyl starter unit and (alkyl)malonyl extender units from CoA to the phosphopantetheine prosthetic group of acyl carrier protein (ACP) domains in the chain initiation module and each chain extension module, respectively^3^. On the other hand, NRPSs use adenylation (A) domains to first activate amino (and other) acids via reaction with ATP, forming aminoacyl adenylates, then transfer the aminoacyl groups onto the phosphopantethiene thiols of peptidyl carrier protein (PCP) domains in each module^4^. Ketosynthase (KS) domains in each chain elongating module of PKSs receive the acyl group attached to the ACP domain in the preceding module onto a conserved active site Cys residue and catalyse decarboxylative two-carbon elongation of the chain with the (alky)malonyl thioester attached to the downstream ACP domain^5^. Similarly, condensation (C) domains in each chain elongating module of NRPSs catalyse elongation of the acyl group attached to the PCP domain in the preceding module with the aminoacyl thioester attached to the downstream PCP domain^6^.

The β-ketothioester resulting from chain elongation by KS domains in PKSs can be further functionalised by optional ketoreductase (KR), dehydratase (DH), and enoyl reductase (ER) domains^7–9^. KR domains control the stereochemistry of the α (where applicable) and β-carbons in their products^10^. Similarly, ER domains control the stereochemistry of the α-carbon^11^. Some DH domains catalyse sequential dehydration of β, δ-dihydroxy thioesters to form the corresponding dienes^12^. The stereochemical outcomes of DH domain-catalysed dehydration reactions are dictated by the stereochemistry of the β (and δ) carbons in the substrates^12,13^.

NRPS A domains typically activate and load l-configured amino acids, but the stereochemistry of the α-carbon in the resulting aminoacyl thioesters gets inverted if an optional epimerisation (E) domain is also present in the module, or if a bifunctional E/C domain is present in the downstream module^14,15^. Occasionally, NRPS modules contain *N* or *C*-methyltransferase (MT) domains, which methylate the amino group or α-carbon of the aminoacyl thioester prior to peptide bond formation^16,17^. C domains can be substituted by bifunctional heterocyclisation (Cy) domains, which catalyse peptide bond formation followed by cyclodehydration to yield an oxazoline or thiazoline, when the substrate attached to the downstream PCP domain is a serinyl, threoninyl, or cysteinyl thioester^18^. Modules with a Cy domain in place of the C domain sometimes also contain a flavin-dependent oxidase (Ox) domain that converts the ox/thiazoline to the corresponding ox/thiazole^19^.

The analogous enzymatic logic employed by PKSs and NRPSs, involving shuttling of thioester intermediates tethered to carrier protein domains between successive catalytic domains, has enabled the evolution of numerous hybrid PKS-NRPS assembly lines^20^. In these systems, an ACP domain at the C-terminus of a PKS subunit frequently interfaces with a C or Cy domain at the N-terminus of an NRPS subunit. This enables one or more amino acids to be grafted onto structurally complex acyl chains assembled by PKSs.

**Fig. 1:**
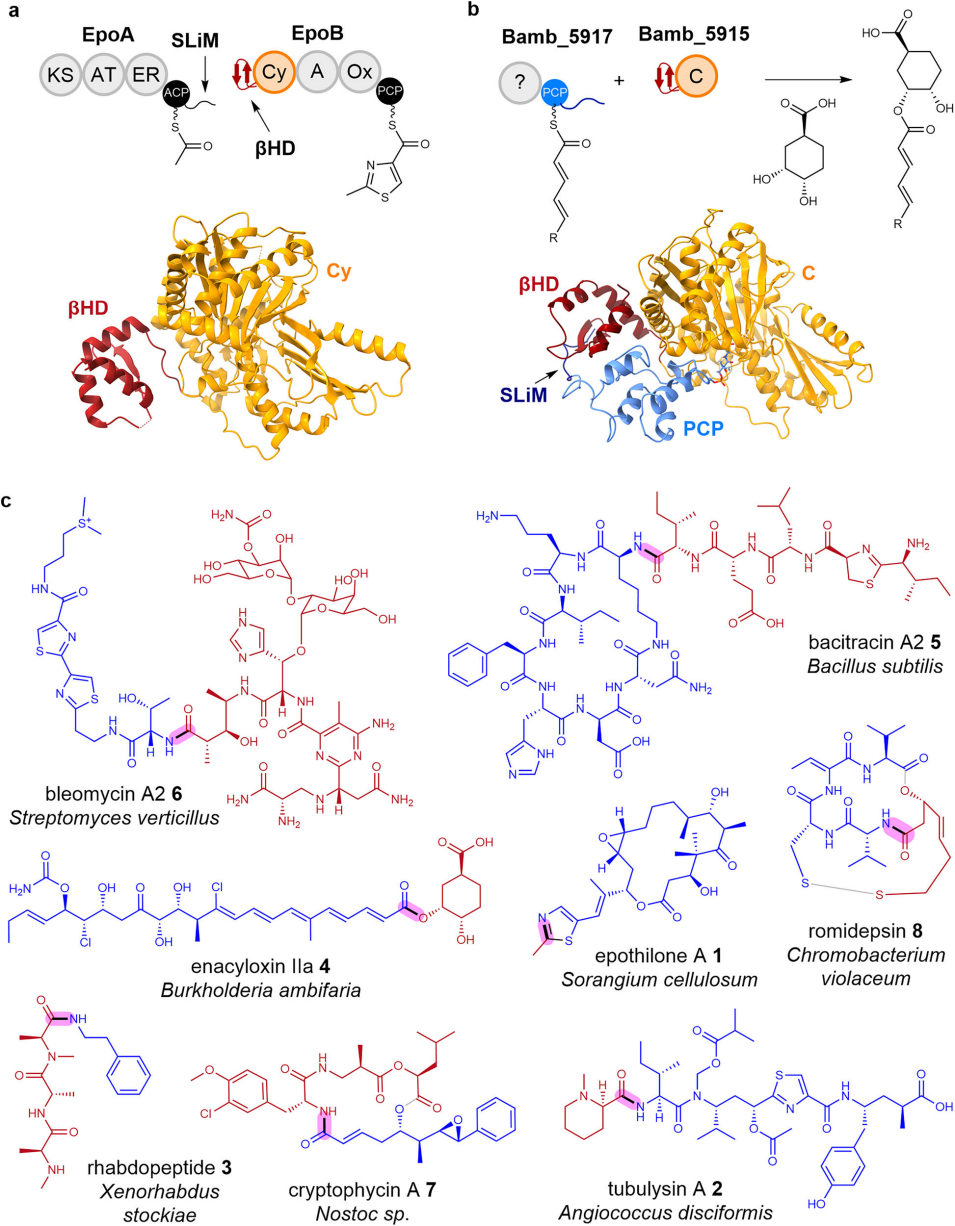
Molecular basis for SLiM/βHD domain-mediated subunit interaction in hybrid PKS-NRPS and NRPS assembly lines and structures of selected bioactive products biosynthesised by systems employing these docking elements. **a** SLiM/βHD domain-mediated interaction of EpnA and EpnB in the hybrid PKS-NRPS that assembles epothilones (top) and X-ray crystal structure of the excised EpoB βHD-Cy di-domain (bottom; PDB ID: 5T7Z). **b** SLiM/ βHD domain-mediated interactions of Bamb_5917 and Bamb_5915, involved in PKS chain release during enacyloxin IIa biosynthesis (top), and a model of the PCP-SLiM / βHD-C tetra-domain complex based on an X-ray structure of Bamb_5915 (PDB ID: 6CGO) and NMR structures of the excised Bamb_5917 PCP-SLiM di-domain (bottom). **c** Structures of selected natural products assembled by hybrid PKS-NRPS and NRPS systems employing SLiM/βHD domain interactions, including several used clinically (bleomycin, bacitracin, epothilone and romidepsin), and others with strong potential to address unmet clinical needs. The diverse taxonomic origin of these systems highlights that SLiM/βHD docking domain-mediated interactions are of widespread importance in bacterial specialised metabolism. The bond formed due to the SLiM/βHD domain-mediated interaction is highlighted in pink, resulting in a union between the fragments coloured blue and red in each example.

Mutually compatible docking domains attached to the C-terminus of the ACP domain and the N-terminus of the C/Cy domain are frequently employed to ensure productive interactions between the PKS and NRPS subunits^21,22^. One class of such docking domains employs a short linear motif (SLiM) appended to the ACP domain to engage with a β-hairpin docking (βHD) domain fused to the C/Cy domain^23–25^. This docking domain class was first identified at the interface between the EpoA and EpoB subunits of the hybrid PKS-NRPS that assemble epothilone A **1**(Fig. 1a)^26,27^. Subsequent structural characterisation of a homologue of the EpoB docking domain excised from the N-terminus of the TubC subunit of the PKS-NRPS that assembles tubulysin A **2** showed it contains a conserved β-hairpin that mediates the docking interaction^23^. This was later confirmed by X-ray crystallographic analysis of the excised Cy domain from EpoB (Fig. 1a)^28^, and studies of rhabdopeptide **3** biosynthesis showed that SLiM-βHD domain pairs can also mediate interactions between NRPS subunits^24^. Extensive biochemical and structural studies of Bamb5917 and Bamb5915, which mediate chain release from the PKS that assembles enacyloxin IIa **4**, provided the first clear insights into how SLiM-βHD domain interactions enable productive association of PCP and C domains (Fig. 1b)^25,29^. Using a hidden Markov model (HMM), βHD domains and associated SLiMs were identified at PKS-NRPS and NRPS-NRPS subunit interfaces in numerous biosynthetic assembly lines^25^. In addition to those described above, these include synthetases for bacitracin A2 **5** (a widely used topical antibiotic), bleomycin A2 **6** (employed to treat diverse forms of cancer), cryptophycin A **7**, a promising anti-tumour agent, and romidepsin **8** (used to treat T-cell lymphomas) (Fig. 1c)^30–33^.

**Fig. 2:**
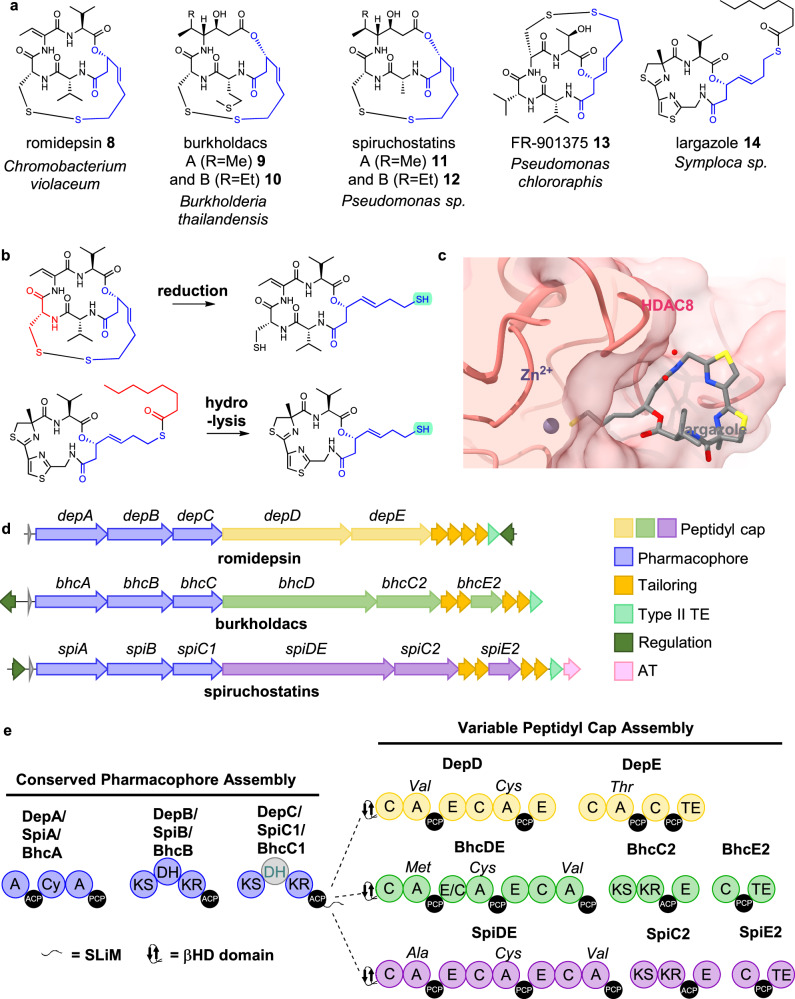
Structures, mechanisms of action, biosynthetic gene clusters and hybrid PKS-NRPS assembly lines of depsipeptide HDAC-inhibiting natural products. **a** Structures of romidepsin, burkholdacs/spiruchostatins, FR-901375 and largazole, representing four distinct classes of depsipeptide HDAC inhibitors. The conserved pharmacophore is highlighted in blue. **b** Two alternative pro-drug activation mechanisms for depsipeptide HDAC inhibitors. The pharmacophore thiol (highlighted in green) is unmasked in cellulo either by reduction (disulfide-containing prodrugs; top) or hydrolysis (thioester-containing prodrugs, bottom). Masking residues are highlighted in red. **c** X-ray crystal structure of HDAC8-largazole complex (PDB ID: 3RQD) showing the unmasked pharmacophore thiol group coordinated to the active site Zn^2+^ ion. **d** Organisation of the romidepsin (*dep*), burkholdac (*bhc*) and spiruchostatin (*spi*) BGCs. The FR-901375 BGC was identified in this study (Fig. 3a), whereas the largazole BGC remains to be discovered. **e** The domain organisation of the conserved megasynthetase machinery for pharmacophore assembly (in blue; a putatively inactive DH domain is in grey) and the divergent megasynthetase machinery for biosynthesis of the variable peptidyl caps in romidepsin and the burkholdacs/spiruchostatins (in various other colours). The domain organisation in the first conserved pharmacophore assembly subunit was incorrectly assigned in previous reports^30,34,35^.

Romidepsin **8** belongs to a family of structurally diverse depsipeptides produced by Gram-negative bacteria, all of which potently inhibit class I histone deacetylases (HDACs). Other members of this family include the burkholdacs A **9** and B **10**, spiruchostatins A **11** and B **12**, FR-901375 **13**, and largazole **14** (Fig. 2a)^34–37^. Members of this family employ two related pro-drug mechanisms, whereby disulfide reduction (**8**–**13**) or thioester hydrolysis (**14**) in target cells unmasks the terminal thiol group of the conserved pharmacophore, which coordinates to the catalytically essential Zn^2+^ ion in the active site of HDACs (Fig. 2b, c)^38^. Differences in the selectivity of these compounds toward different HDAC isoforms has been attributed to their structurally diverse peptidyl caps, which interact with the outer rim of the active site tunnel in HDACs, as illustrated by the X-ray crystal structure of the HDAC8-largazole complex (Fig. 2c)^39^.

The biosynthetic gene clusters (BGCs) for romidepsin, the spiruchostatins, and burkholdacs have been identified in *Chromobacterium violaceum* no. 968, *Pseudomonas sp*. Q71576 and *Burkholderia thailandensis* E264, respectively (Fig. 2d)^21,26,27^, whereas those for FR-901375 and largazole remain to be discovered. The romidepsin, burkholdac and spiruchostatin BGCs encode a highly conserved NRPS-PKS that is proposed to assemble the common pharmacophore. An NRPS in the romidepsin BGC, and architecturally similar NRPS-PKSs in the burkholdac and spiruchostatin BGCs, are hypothesised to fuse diverse tetra and tripeptidyl caps, respectively, with the conserved pharmacophore (Fig. 2e). Although the romidepsin BGC was identified almost two decades ago, several important aspects of despipeptide HDAC inhibitor biosynthesis remain mysterious. Our previously reported HMM^25^, identified βHD domains at the N-terminus of the first (NRPS) subunit of the variable peptidyl cap biosynthetic apparatus and corresponding SLiMs were found appended to the C-terminus of the last (PKS) subunit of the conserved machinery for pharmacophore assembly encoded by the romidepsin, burkholdac, and spiruchostatin BGCs (Fig. 2e and Supplementary Fig. 1). Previous bioinformatics analyses of these BGCs failed to identify these important docking elements^30,34,35^. We hypothesised that interactions between the SLiMs and βHD domains, and their associated ACP and C domains, likely play a critical role in the evolution of depsipeptide HDAC inhibitor structural diversity by enabling the machinery for conserved pharmacophore assembly to engage productively with diverse peptidyl cap biosynthetic systems.

Here we report the discovery of the hitherto unknown BGC for FR-901375 in the previously unreported producer *Pseudomonas chlororaphis* subsp. *piscium* DSM 21509 using gene proximity searching of a public sequence repository, LC-MS/MS comparisons with an authentic synthetic standard, and gene deletion experiments. Comparative analysis of the FR-901375 BGC and the proteins it encodes suggests it evolved from the spiruchostatin BGC via horizontal transfer of a gene encoding an NRPS with a compatible N-terminal β-HD domain that subsequently underwent a series of duplication and recombination events to enable assembly of a distinct tetrapeptidyl cap. Recombination within the genes encoding the original peptidyl cap biosynthetic machinery appears to have subsequently eliminated the capability to produce spiruchostatins. In vitro reconstitution of the reactions catalysed by the first modules of the NRPSs mediating variable cap assembly in romidepsin, burkholdac, and FR-901375 biosynthesis enables us to demonstrate productive engagement with the conserved pharmacophore biosynthetic apparatus from all four systems, establishing the molecular basis for combinatorial biosynthesis of this important class of HDAC-inhibiting depsipeptides. Using a combination of mutagenesis, AlphaFold models, and carbene footprinting mass spectrometry (MS), we evaluate the roles played by the SLiM and βHD domain and their associated ACP and C domains in ensuring productive interaction between the pharmacophore and peptidyl cap biosynthetic machineries. We conclude that the βHD domain employs a unique mechanism to facilitate productive engagement of PKS and NRPS subunits, involving direct binding to a conserved epitope on the SLiM-bearing ACP domain.

## Results

### Discovery of FR-901375 biosynthetic gene cluster

**Fig. 3:**
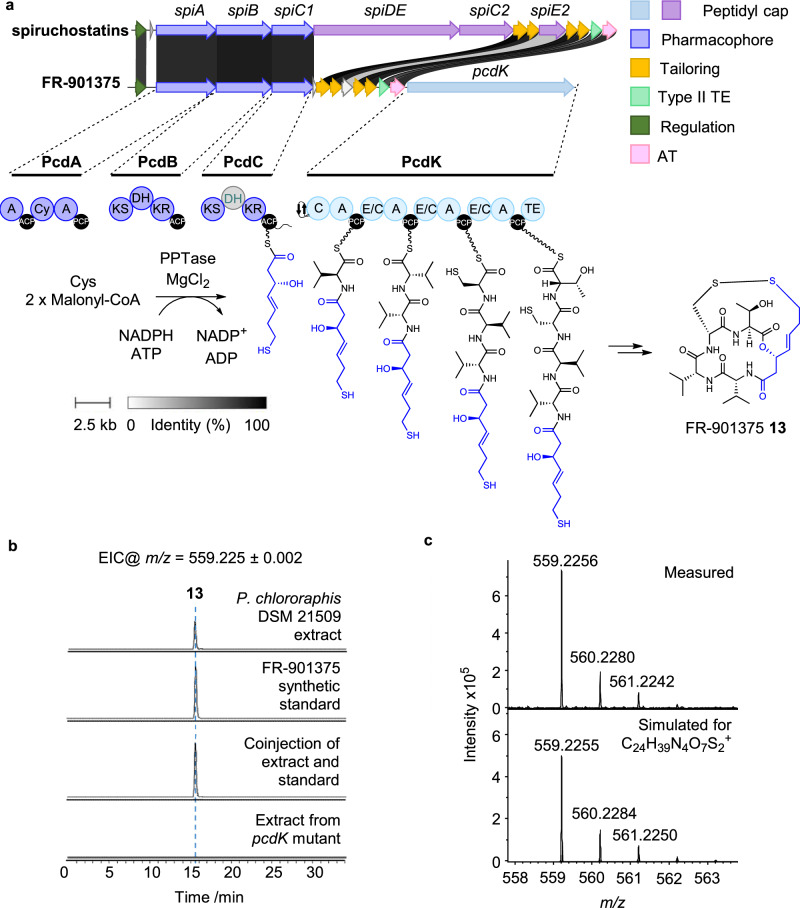
Comparison of the FR-901375 BGC in *P. chlororaphis* subsp. *piscium* DSM 21509 to the spiruchostatin BGC, proposed domain organisation of the FR-901375 PKS-NRPS, and confirmation that this BGC directs FR-901375 biosynthesis. **a** Comparison of the spiruchostatin and putative FR-901375 BGCs, illustrating the proposed domain organisation and substrates of the PKS and NRPS subunits encoded by the latter. The pharmacophore transferred by the PKS is shown in blue. **b** Extracted ion chromatograms at *m/z* = 559.2255 (corresponding to [M + H]^+^ for FR-901375) from UHPLC-ESI-Q-TOF-MS analyses of extracts of *P. chlororaphis* subsp. *piscium* DSM 21509 (top), a *pcdK* mutant (bottom), a synthetic standard of FR-901375 (second from top), and a co-injection (second from bottom). **c** Comparison of the observed high-resolution mass spectrum (top) for the [M + H]^+^ ion of FR-901375 in *P. chlororaphis* subsp. *piscium* DSM 21509 extracts with the simulated spectrum (bottom).

Based on the common features of the romidepsin, spiruchostatin, and burkholdac BGCs, we anticipated the FR-901375 BGC to contain a set of genes encoding a hybrid NRPS-PKS for assembly of the conserved pharmacophore and a gene encoding an NRPS with an N-terminal βHD domain. Thus, we used clusterTools to search the antiSMASH database 2.0 for BGCs containing genes encoding these features (Supplementary Table 1)^40^, using NRPS-PKS HMMs from antiSMASH 5.0 and a previously reported HMM for the βHD domain. This search returned 15 hits (Supplementary Table 2). Among these, a BGC in *P. chlororaphis* subsp. *piscium* DSM 21509 was of greatest interest, because the conserved pharmacophore genes (*pcdA-B-C*) and variable peptidyl cap biosynthetic gene (*pcdK*) are juxtaposed differently to other depsipeptide HDAC inhibitor BGCs (Fig. 3a).

On closer inspection, it became apparent that most genes in this BGC encode proteins with a very high degree of similarity to those encoded by the spiruchostatin BGC (Supplementary Table 3). Intriguingly, however, the orthologue of *spiE2*, which encodes the final subunit of the hybrid NRPS-PKS mediating assembly of the spiruchostatin peptidyl cap, contains several substantial in-frame deletions that very likely render the corresponding protein non-functional (Supplementary Fig. 1). Moreover, the 5′ end of the *spiDE* and 3′ end of the *spiC2* orthologues, which encode the first and second subunits, respectively, of the peptidyl cap NRPS-PKS, have been concatenated via another series of in-frame deletion events, resulting in a pseudogene containing 140 of the first 149 bases of *spiDE* fused to the last 94 bases of *spiC2* (Supplementary Fig. 1).

In lieu of the *spiDE*, *spiC2* and *spiE2* orthologues, the BGC has an additional gene (*pcdK*) at its right flank that encodes an NRPS with a relatively low level of overall sequence similarity to the NRPS subunits involved in assembling the peptidyl caps of romidepsin/burkholdacs/spiruchostatins (40–60%). Sequence analysis of the protein encoded by *pcdK* revealed that it contains four modules, indicating that it assembles a tetrapeptide. The substrate specificities of the A domains in modules 1–4 were predicted to be l-Val, l-Val, l-Cys and l-Thr, respectively (Supplementary Table 4)^41^. The second, third, and fourth modules contain bifunctional E/C domains, and a thioesterase (TE) domain is appended to the C-terminus of module 4, suggesting, overall, that PcdK catalyses assembly and attachment of a tetrapeptidyl cap with the sequence d-Val-d-Val-d-Cys-l-Thr to the conserved pharmacophore of a depsipeptide HDAC inhibitor (Fig. 3a). The *pcdH* gene encodes a homologue of DepH, which has been shown to catalyse disulfide bond formation between the Cys residue in the peptidyl cap and the terminal thiol of the conserved pharmacophore in romidepsin biosynthesis^42^. We therefore propose that PcdH catalyses an analogous reaction (Fig. 3a), noting that the Cys residue in the peptidyl cap assembled by PcdK is in a different position from the corresponding residue in the peptidyl caps of other disulfide-containing HDAC inhibitors.

The predicted PcdK product is identical in composition and stereochemistry to the tetrapeptidyl cap of FR-901375. Thus, we hypothesised that the BGC we identified via our gene proximity searching approach directs FR-901375 biosynthesis. To verify this hypothesis, *P. chlororaphis* subsp. *piscium* DSM 21509 was grown in LB supplemented with 0.5% HP-20 and XAD-16 resins at 30 °C for 72 h, and the metabolites adsorbed onto the resins were eluted with ethyl acetate. A compound with *m/z* = 559.2256, corresponding to the [M + H]^+^ ion of FR-901375 (calculated *m/z* = 559.2255) was observed in UHPLC-ESI-Q-TOF-MS analyses of the eluate (Fig. 3b, c). To confirm the identity of this compound, we conducted comparative UHPLC-ESI-Q-TOF-MS/MS analyses with a synthetic standard of FR-901375^43^. The synthetic standard and the extracted metabolite had the same retention time and MS/MS spectrum (Fig. 3b and Supplementary Fig. 2). To further confirm that the BGC identified in *P. chlororaphis* subsp. *piscium* DSM 21509 directs FR-901375 biosynthesis an in-frame deletion in *pcdK* was constructed (Supplementary Fig. 3). UHPLC-ESI-Q-TOF-MS analysis of the resin eluates from cultures of the mutant confirmed FR-901375 production was abolished (Fig. 3b).

### The βHD domain but not the SLiM is required for FR-901375 biosynthesis

Conservation of the SLiM and βHD domain at the C and N-terminus, respectively, of the conserved pharmacophore and variable peptidyl cap biosynthetic machineries for assembly of romidepsin, the burkholdacs, the spiruchostatins, and FR-901375 (Supplementary Fig. 4), suggests these docking elements play an important role in bicyclic depsipeptide HDAC inhibitor assembly. To investigate this, regions of *pcdC* encoding the ACP-SLiM di-domain and *pcdK* encoding the βHD-C-A-PCP tetra-domain were cloned from *P. chlororaphis* subsp. *piscium* DSM 21509 genomic DNA into a modified pET28a(+) vector containing three mutations that convert the N-terminal MGSSH_6_ affinity purification tag to MKH_8_. Mutation of the second residue from G to K minimises gluconylation of N-terminal poly-His fusion proteins, which can complicate intact protein MS analyses and reduce signal intensity^44^. The resulting His_8_ fusions were overproduced in *E. coli* and purified to homogeneity using nickel affinity chromatography (Supplementary Fig. 5).

**Fig. 4:**
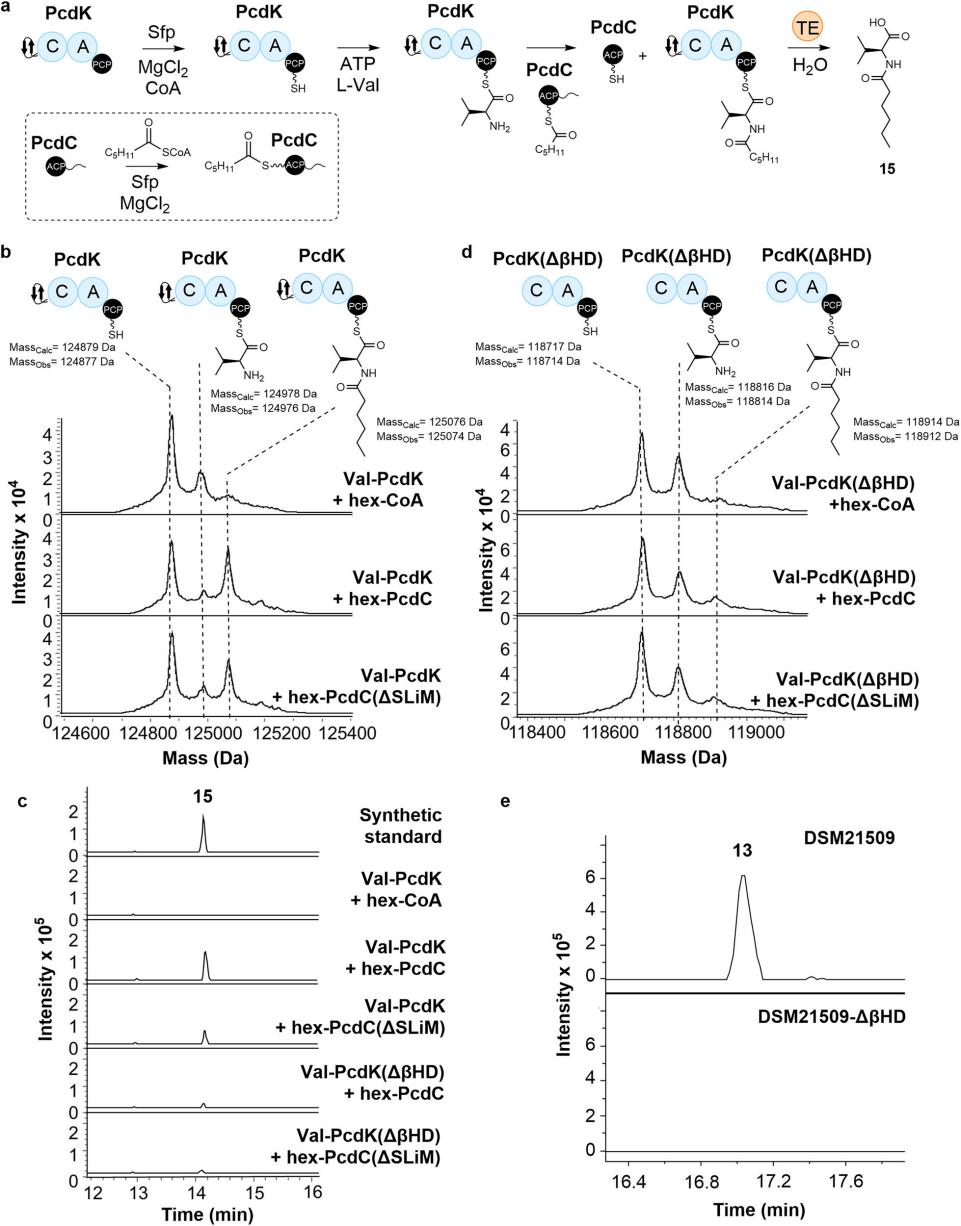
Reconstitution of chain elongation across the interface between the pharmacophore and peptidyl cap assembly apparatus in FR-901375 biosynthesis and investigation of the role played by the SLiM and βHD domains. **a** Schematic representation of an enzymatic assay for chain elongation across the PcdC-PcdK interface. The inset framed by the dotted line illustrates the procedure used to create the PcdC hexanoyl-ACP-SLiM di-domain (and derivatives). Each step along the path to production of compound **15** was validated by intact protein MS. The type II thioesterase (TE) used to liberate **15** is encoded by *bamb*_*_*_*5926* in the enacyloxin IIa BGC^60^. **b** Intact protein mass spectra of the PcdK valinyl-βHD-C-A-PCP tetra-domain after incubation with hexanoyl-CoA, the hexanoyl-ACP-SLiM di-domain and the hexanoyl-ACP-ΔSLiM construct for 2 h. **c** Extracted ion chromatograms at *m/z* = 216.1590 ± 0.002 (corresponding to [M + H]^+^ for **15**) from UHPLC-ESI-Q-ToF-MS analyses of hydrolytic release products resulting from incubation of the valinyl-βHD-C-A-PCP tetra-domain with hexanoyl-CoA, the hexanoyl-ACP-SLiM di-domain, or the hexanoyl-ACP-ΔSLiM construct, and the valinyl-ΔβHD-C-A-PCP construct with the hexanoyl-ACP-SLiM di-domain, or hexanoyl-ACP-ΔSLiM construct. **d** Intact protein mass spectra of the valinyl-ΔβHD-C-A-PCP construct after incubation with hexanoyl-CoA, the hexanoyl-ACP-SLiM di-domain, and the hexanoyl-ACP-ΔSLiM construct. **e**, Extracted ion chromatograms at *m/z* = 559.2255 ± 0.002 (corresponding to [M + H]^+^ for FR-901375) from UHPLC-ESI-Q-TOF-MS analyses of culture extracts from *P. chlororaphis* subsp. *piscium* DSM 21509 and the mutant with an in-frame deletion in the βHD domain.

The purified tetra-domain was confirmed to be catalytically active by converting it to the *holo* form using the phosphopantetheinyl transferase Sfp and demonstrating the ability of the A domain to load an l-valinyl residue onto the PCP domain using intact protein MS (Fig. 4a and Supplementary Fig. 7). To probe the ability of the tetra-domain to engage productively with the purified ACP-SLiM construct, we exploited the broad substrate tolerance of Sfp to load a hexanoyl thioester onto the *apo*-ACP domain (Fig. 4a and Supplementary Fig. 7)^45^. The hexanoyl group serves as a simplified mimic of the pharmacophore proposed to be assembled by PcdA, PcdB and PcdC. After 2 h incubation of the hexanoyl-ACP-SLiM di-domain with an equimolar quantity of the valinyl-βHD-C-A-PCP tetra-domain at room temperature, intact protein MS analysis revealed a new species attached to the latter. This had a mass 98 Da greater than the valinylated tetra-domain, consistent with formation of an *N*-hexanoyl-valinyl thioester (Fig. 4b). Hydrolytic cleavage of the thioester using a promiscuous type II thioesterase and UHPLC-ESI-Q-TOF-MS comparison of an organic extract with a synthetic standard confirmed it was *N*-hexanoyl-l-valine **15** (Fig. 4c).

The regions encoding the SLiM and βHD domain in the ACP-SLiM di-domain and βHD-C-A-PCP tetra-domain expression vectors were deleted using site-directed mutagenesis. The resulting PcdC ACP-ΔSLiM and PcdK ΔβHD-C-A-PCP constructs were overproduced and purified, and their integrity was confirmed by intact protein MS (Supplementary Fig. 5). Size exclusion chromatography of the PcdK βHD-C-A-PCP and C-A-PCP constructs showed they have the same oligomerisation state (Supplementary Fig. 6). After loading with substrates as described above (Supplementary Fig. 7), the hexanoyl-ACP-ΔSLiM construct was incubated with the valinyl-βHD-C-A-PCP tetra-domain, the hexanoyl-ACP-SLiM di-domain was incubated with the valinyl-ΔβHD-C-A-PCP construct, and the hexanoyl-ACP-ΔSLiM and valinyl-ΔβHD-C-A-PCP constructs were incubated with each other for 2 hours. Trace quantities of *N*-hexanoyl-valinyl thioester and **15** were observed in the intact protein MS and hydrolytic cleavage assays, respectively, for all experiments involving the construct lacking the βHD domain. In contrast, significant quantities of the *N*-hexanoyl-valinyl thioester were produced in experiments where only the SLiM is absent (Fig. 4c, d). This suggests that the βHD domain plays a critical role in FR-901375 biosynthesis. To confirm this, an in-frame deletion was introduced into the region encoding it in *P. chlororaphis* subsp. *piscium* DSM 21509. The resulting mutant was unable to produce FR-901375 (Fig. 4e). The trace amount of product formation in all assays employing the valinyl-ΔβHD-C-A-PCP construct likely results from the relatively high concentration of proteins used for these in vitro experiments.

To investigate whether the SLiM is dispensable in the three previously known HDAC inhibitor assembly lines, we attempted to overproduce and purify the corresponding ACP-SLiM di-domains, ACP domains, and βHD-C-A-PCP tetra-domains (Supplementary Fig. 8). An L14Y mutation was introduced into the BhcC ACP-SLiM di-domain to facilitate protein quantification by UV absorbance. The tetra-domain from SpiDE was insoluble. Thus, it could not be used in subsequent experiments. Employing an analogous assay to that used to examine the interaction of the PcdK βHD-C-A-PCP tetra-domain and the PcdC ACP-SLiM di-domain, the BhcDE and DepD tetra-domains were converted to their *holo* form using Sfp, then methioninylated and valinylated, respectively, by incubation with the requisite amino acid and ATP. (Supplementary Fig. 9). The *apo*-ACP-SLiM di-domains and *apo*-ACP domains excised from BhcC and DepC were loaded with a hexanoyl thioester and incubated with the cognate aminoacyl-βHD-C-A-PCP tetra-domain. Intact protein MS analysis of *N*-hexanoyl-aminoacyl thioester formation on the βHD-C-A-PCP tetra-domains and UHPLC-ESI-Q-TOF-MS analysis of *N*-hexanoyl-amino acid production following hydrolytic offloading showed that deletion of the SLiM did not affect the burkholdac system, while it was moderately detrimental to the romidepsin system (Supplementary Fig. 10).

### Structural basis for the interaction of the pharmacophore and the cap machineries

**Fig. 5:**
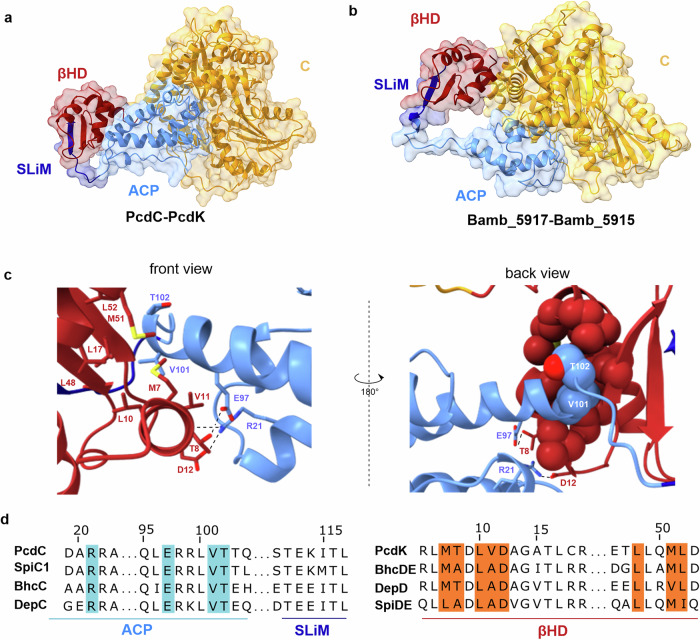
Comparison of structural models for complexes of carrier protein-SLiM (light blue/dark blue) and βHD-C (red/yellow) di-domains from the FR-901375 and enacyloxin IIa assembly lines and the role played by the βHD domain in positioning the ACP domain in depsipeptide HDAC inhibitor biosynthesis. **a** AlphaFold model of the PcdC ACP-SLiM di-domain complexed to the PcdK βHD-C di-domain. **b** AlphaFold model of the Bamb_5917 PCP-SLiM di-domain complexed to the Bamb_5915 βHD-C di-domain. **c** Region of the AlphaFold model of the PcdC-PcdK complex, highlighting residues mediating contact between the first β-sheet and the first and last α-helices of the βHD domain (in red) and the *N* and *C*-termini of the first and last α-helices, respectively, of the ACP domain (in blue). A space-filling representation is used to highlight the interaction between side chains of V101 and T102 of the ACP domain and the cluster of hydrophobic residues in the βHD domain. Key inter-domain hydrogen bonds and salt bridges are indicated by dashed lines. **d** Part of a sequence alignment of the ACP-SLiM di-domains (left) and βHD domains (right) in the PcdC/PcdK, SpiC1/SpiDE, BhcC/BhcDE and DepC/DepD proteins, highlighting that residues mediating the interaction between the first β-sheet/the first and last α-helices of the βHD domain (in orange) and the N/C-termini of the first/last α-helices, respectively, of the ACP domain (in blue) are highly conserved. The T8A mutation in SpiDE and BhcDE appears to be compensated for by shortening the hydrogen bond between the side chain of the R21 and the backbone carbonyl group of the A8 residue.

To illuminate the structural role played by the βHD domain in enabling productive interaction between the pharmacophore and peptidyl cap biosynthetic machineries, an AlphaFold model of the complex between the PcdC ACP-SLiM and PcdK βHD-C di-domains was constructed (Fig. 5a). Comparison with an AlphaFold model of the complex between the Bamb_5917 PCP-SLiM and Bamb5915 βHD-C di-domains (Fig. 5b), which is very similar to the previously published experimentally derived structural model (Supplementary Fig. 11)^25^, revealed significant differences. In the Bamb_5917/Bamb_5915 complex, the βHD domain interacts primarily with the SLiM, which docks onto the second β-sheet of the β-hairpin. While a similar interaction occurs between the SLiM and βHD domain in the PcdC/PcdK complex, the βHD domain also binds directly to the ACP domain via a network of hydrophobic and polar contacts. This involves a hydrophobic protuberance formed by the V101 and T102 side chains at the C-terminus of the last α-helix of the ACP domain, which nestles in a hydrophobic pocket formed by the side chains of M7, L10, V11, T16, L17, L48, M51 and L52 of the βHD domain (Fig. 5c). The side chain of R21 at the *N*-terminus of the first α-helix of the ACP domain forms a salt bridge with the side chain of E97 at the C-terminus of the last α-helix, pinning the ends of the ACP domain to each other (Fig. 5c). In addition, R21 in the ACP domain forms a salt bridge with the side chain of D12 and donates a hydrogen bond to the backbone carbonyl group of T8 in the βHD domain. The side chain of E97 at the C-terminus of the last α-helix of the ACP domain, accepts a hydrogen bond from the side chain of T8 in the βHD domain (Fig. 5c). Comparison with AlphaFold models of the complexes between the ACP-SLiM and βHD-C di-domains from the romidepsin, burkholdac, and spiruchostatin assembly lines indicate that the interaction of the first β-sheet and three α-helices of the βHD domain with first and last α-helices of the ACP domain exhibits a high degree of structural conservation across all four systems (Supplementary Fig. 12). Accordingly, the residues involved in mediating this interaction are also highly conserved (Fig. 5d).

The AlphaFold models predict the main points of interaction between the ACP and C domains in the four systems to be the second α-helix and the helical-loop region connecting this to the C-terminus of the first α-helix in the ACP domain with the floor loop and the C-terminus of α-helix 10 and the downstream loop in the C domain (Supplementary Fig. 12). However, a well-defined interaction epitope is not observed, even though the interfacial residues in these regions of the ACP and C domains are highly conserved. Despite this, all four models place the conserved Ser residue in the ACP domain, which bears the phosphopantetheine arm that delivers the substrate to the active site, near the entrance to the active site tunnel in the respective C domain (Supplementary Fig. 13). The complex between the PcdC ACP-SLiM and PcdK βHD-C di-domains remains stably associated throughout 500 ns MD simulations (Supplementary Fig. 13).

As an initial experimental validation of the AlphaFold models, carbene footprinting MS was conducted^46^. This technique utilises a photoreactive diazirine to probe the solvent accessibility of protein surface residues in the presence and absence of a binding partner. Photolysis of the diazirine yields a highly reactive carbene that rapidly inserts into surface-accessible residues. Proteolytic digestion of the proteins enables the extent of peptide-level labelling to be monitored. Decreased labelling in the presence of a binding partner (masking) is indicative of reduced solvent accessibility, and increased labelling (unmasking) is suggestive of increased solvent accessibility. While masking can result from both a binding interaction and a conformational change upon complex formation, unmasking typically only arises from the latter. We have previously used carbene footprinting MS, in conjunction with other techniques, to map diverse protein-protein interaction interfaces in several PKS and NRPS systems^25,47–50^.

The PcdK βHD-C di-domain was genetically excised from the tetra-domain construct used in the experiments described above, then overproduced, purified and verified using intact protein MS (Supplementary Fig. 4). The interaction of the PcdC ACP-SLiM and PcdK βHD-C di-domains was then investigated using carbene footprinting MS. Fractional modification of tryptic peptides was analysed, and masked/unmasked regions were mapped onto AlphaFold models of the ACP-SLiM and βHD-C di-domains (Supplementary Figs. 14–17).

**Fig. 6:**
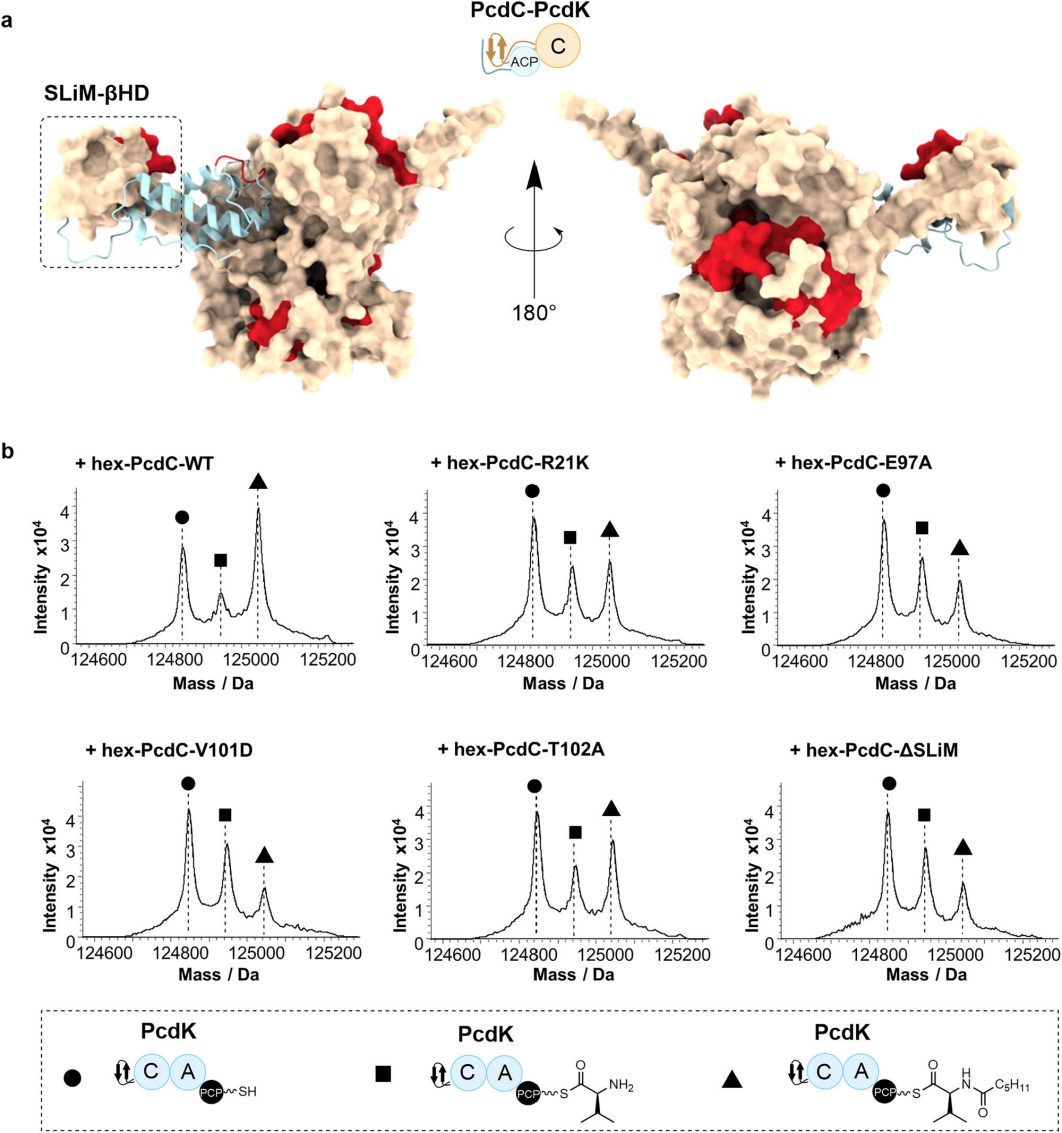
Carbene footprinting MS of PcdC ACP-SLiM/PcdK βHD-C di-domain complex and site-directed mutagenesis of PcdC ACP-SLiM di-domain residues predicted to contact the PcdK βHD domain. **a** AlphaFold model of ACP-SLiM/βHD-C di-domain complex with peptides masked in carbene footprinting MS analysis highlighted in red. **b** Intact protein MS analysis of hexanoyl-valinyl thioester formation on the PcdK βHD-C-A-PCP tetra-domain following incubation for 20 min with the wild-type (WT) PcdC hexanoyl-ACP-SLiM di-domain and R21K, E97A, V101D, T102A and ΔSLiM mutants.

Masking was observed in a loop region of the ACP domain proximal to the phosphopantetheinylated serine, where it contacts the C domain, and in the N-terminal helix of the βHD domain, where it contacts the C-terminus of the final helix in the PCP domain (Fig. 6a). Both are consistent with the structure of the complex predicted by AlphaFold. Several regions of masking were also observed in globular parts of the C domain not in direct contact with the ACP domain (Fig. 6a). Moreover, regions of unmasking were observed in both the ACP-SLiM di-domain and the C domain (Supplementary Fig. 16b). The remote regions of masking and regions of unmasking are indicative of significant conformational changes in both the ACP-SLiM di-domain and the C domain upon complex formation. However, the precise motions involved are difficult to assign without further data.

R21K, E97A, V101D and T102A mutants of the PcdC ACP-SLiM di-domain were constructed (Supplementary Fig. 18) to further verify the validity of the interaction epitope with the PcdK βHD-C di-domain predicted by AlphaFold. Each of the mutants was loaded with hexanoyl-phosphopantetheine (Supplementary Fig. 19) and incubated with the valinyl-βHD-C-A-PCP tetra-domain. After 20 min, the reaction was quenched, and the βHD-C-A-PCP tetra-domain was analysed by intact protein MS. In each case, a significant drop in the level of the *N*-hexanoyl-valinyl thioester, relative to the valinyl thioester, was observed compared to the reaction employing the wild-type hexanoyl-ACP-SLiM di-domain (Fig. 6b). Interestingly, *N*-hexanoyl-valinoyl thioester formation was also significantly affected when the hexanoyl-ACP- ΔSLiM mutant was used. However, this was of a similar magnitude to the V101D mutant, consistent with the SLiM making a relatively modest contribution to the overall stabilisation of the ACP-SLiM / βHD-C di-domain complex (Fig. 6b).

### Noncognate pharmacophore and cap machineries crosstalk productively

The above experiments established that the βHD domains in depsipeptide HDAC inhibitor assembly lines employ a highly conserved epitope to bind the final ACP domain in the conserved pharmacophore biosynthetic apparatus, enabling productive interaction with the first C domain of the variable peptidyl cap machinery. This led us to hypothesise that the ACP-SLiM di-domains from the pharmacophore apparatus should be able to crosstalk productively with the first module of the peptidyl cap machinery in each noncognate system.

**Fig. 7:**
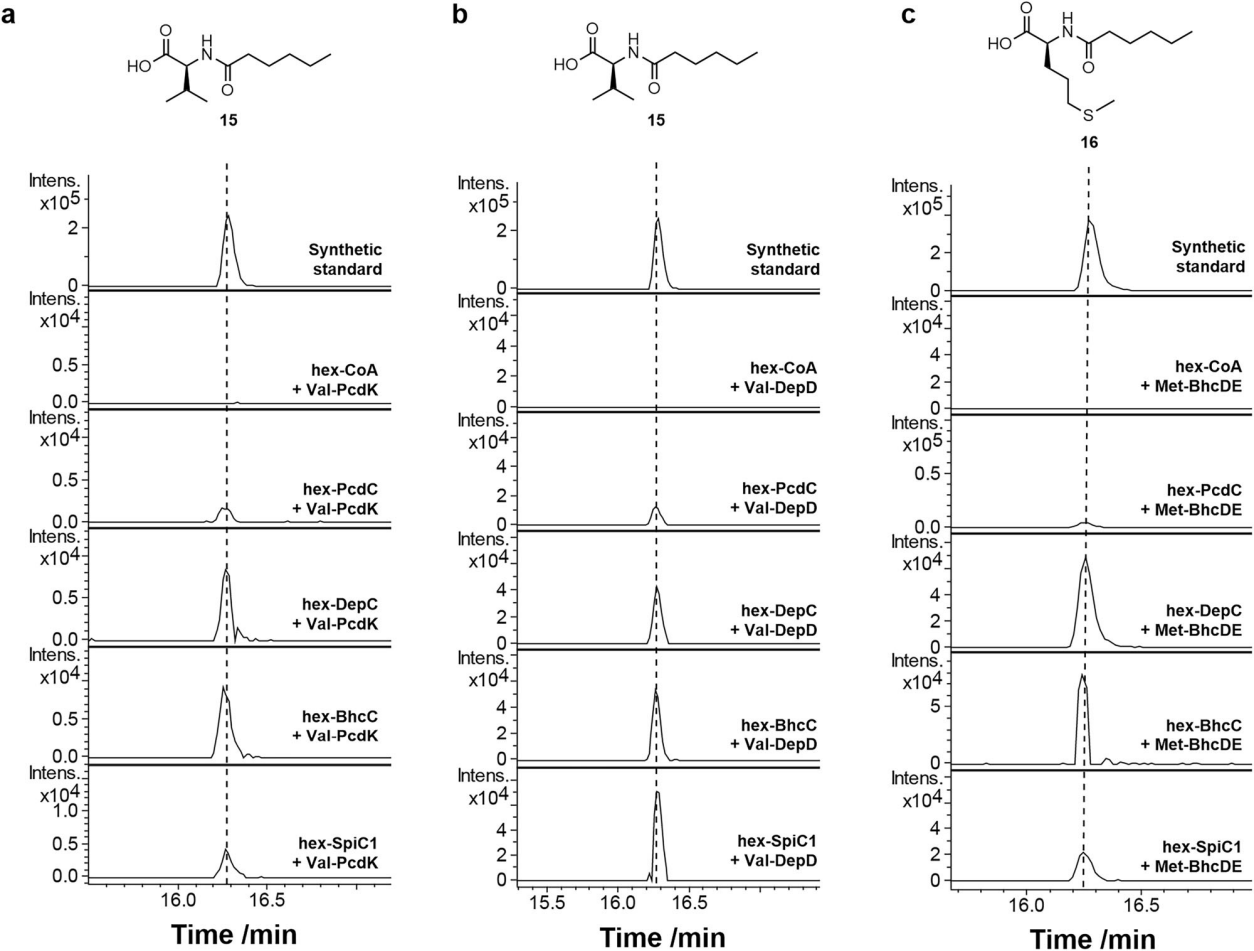
UHPLC-ESI-Q-ToF-MS analysis of *N*-hexanoyl amino acids in organic extracts of reactions of cognate and noncognate hexanoyl-ACP-SLiM di-domains with valinyl/methioninyl-βHD-C-A-PCP tetra-domains and subsequent type II TE-mediated hydrolytic cleavage. **a**, Extracted ion chromatogram at 216.1600 ± 0.002 Da (corresponding to the [M + H]^+^ ion for **15**) from reactions of the PcdC, DepC, BhcC and SpiC1 hexanoyl-ACP-SLiM di-domains with the PcdK valinyl-βHD-C-A-PCP tetra-domain. **b** Extracted ion chromatogram at 216.1600 ± 0.002 Da (corresponding to the [M + H]^+^ ion for **15**) from reactions of the PcdC, DepC, BhcC and SpiC1 hexanoyl-ACP-SLiM di-domains with the DepD valinyl-βHD-C-A-PCP tetra-domain. **c** Extracted ion chromatogram at 248.1315 ± 0.002 Da (corresponding to the [M + H]^+^ ion for **16**) from reactions of the PcdC, DepC, BhcC and SpiC1 hexanoyl-ACP-SLiM di-domains with the BhcDE valinyl-βHD-C-A-PCP tetra-domain. The second from the top spectrum in each case is a negative control to which hexanoyl-CoA was included in place of the hexanoyl-ACP-SLiM di-domains.

To probe the ability of each of the PcdC, DepD and BhcDE tetra-domains to engage productively with the purified PcdC, DepC, BhcC and SpiC1 ACP-SLiM di-domains, we loaded a hexanoyl thioester as a simplified mimic of the pharmacophore onto the *apo*-ACP domain in each of the latter (Supplementary Fig. 9). Each hexanoyl-ACP-SLiM di-domain was separately incubated with an equimolar quantity of the valinyl/methionyl-βHD-C-A-PCP tetra-domains. After 2 h incubation, the thioesters were hydrolysed using the promiscuous Bamb_5926 type II thioesterase. UHPLC-ESI-Q-TOF-MS comparison of organic extracts with synthetic standards of *N*-hexanoyl-l-valine **15** and *N*-hexanoyl-l-methionine **16** confirmed formation of the expected *N*-hexanoyl-amino acid by all the ACP-SLIM di-domain and βHD-C-A-PCP tetra-domain pairs (Fig. 7). Overall, these data demonstrate that noncognate pharmacophore and peptidyl cap biosynthetic machineries can engage in productive crosstalk, providing further experimental validation of the highly conserved interaction epitope between the ACP and βHD domains predicted by AlphaFold.

## Discussion

Our discovery of the FR-901375 BGC and characterisation of key biosynthetic elements, reported here, significantly advances understanding of mechanisms for depsipeptide HDAC inhibitor assembly. This clinically important group of polyketide-nonribosomal peptide hybrids is constructed via a remarkable example of natural combinatorial biosynthesis, requiring a highly conserved apparatus for pharmacophore assembly to interface with diverse machinery for variable peptidyl cap incorporation. Although sequencing of the BGC for a depsipeptide HDAC inhibitor (romidepsin) was reported almost two decades ago^30^, little progress has been made in understanding mechanisms for assembly of this valuable lymphoma drug. Indeed, until our 2019 discovery of the widespread prevalence of βHD domains and associated SLiMs in diverse and important NRPS and hybrid PKS-NRPS assembly lines^25^, a key mediator between the conserved pharmacophore and variable peptidyl cap biosynthetic machineries remained hidden in plain sight.

Our detailed genetic, biochemical, and structural investigations of the roles played by the SLiM and βHD domain in mediating productive engagement of the pharmacophore and peptidyl cap biosynthetic machineries in FR-901375 assembly have uncovered significant differences from other systems studied to date^23–28,51^. For example, both the SLiM fused to the C-terminus of Bamb_5917 and the βHD domain appended to the N-terminus of Bamb_5915 play important roles in mediating subunit interaction in enacyloxin IIa biosynthesis^25^. In contrast, while the βHD domain at the N-terminus of the peptidyl cap machinery is critical for FR-901375 biosynthesis, the SLiM at the C-terminus of the pharmacophore machinery makes only a modest contribution to subunit association in vitro and is dispensable in vivo. A structural model of the complex between the ACP-SLiM and the βHD-C di-domains, validated by carbene footprinting MS data and mutagenesis, explains these observations. The βHD domain employs a previously uncharacterised epitope to directly grasp the globular region of the ACP domain, positioning it to engage productively with the C domain. Comparison with structural models of the corresponding complexes from the romidepsin, burkholdac and spiruchostatin systems reveals that the interactions between the ACP domain and the epitope on the βHD domain are highly conserved (Supplementary Fig. 12). Although the canonical binding of the SLiM to the second β-sheet of the βHD domain is predicted by these models, the interactions mediating this are less well conserved and involve primarily hydrophobic contacts. This may explain why the SLiM is dispensable for FR-901375 biosynthesis in vivo and appears to play a weak to moderate role in promoting subunit association in the FR-901375, burkholdac, and romidepsin systems in vitro.

The observation that ACP-SLiM and βHD-C di-domains from noncognate depsipeptide HDAC inhibitor assembly lines engage productively supports the view that the interaction epitope between βHD and ACP domains we have identified plays a key role in the biosynthesis of all members of this clinically important family of anticancer agents. Moreover, this observation provides a cornerstone for developing meaningful insight into the evolutionary mechanisms for combinatorial biosynthesis of polyketide-nonribosomal peptide hybrids. In the case of depsipeptide HDAC inhibitors, a highly conserved network of interactions across the interface between the PKS involved in the final stages of pharmacophore assembly and the NRPS catalysing the initial stages of peptidyl cap assembly clearly plays a crucial role in promoting structural diversification.

Focusing specifically on the question of how the FR-901375 BGC has evolved from its spiruchostatin counterpart, it is notable that the first five domains of the NRPS assembling the variable cap of the burkholdacs are exceptionally similar in sequence to the corresponding domains involved in FR-901375 biosynthesis (69% identity over 1574 amino acid residues). The only dip in sequence similarity occurs in the core substrate binding region of the A domain, which is responsible for incorporation of the Met residue into the burkholdacs and the first Val residue into FR-901375 (Supplementary Fig. 20a). Moreover, BhcDE is the only NRPS encoded by previously known depsipeptide HDAC inhibitor BGCs to contain a bifunctional E/C domain (Fig. 2e), which is present in the second, third, and fourth modules of the NRPS responsible for assembly of the FR-901375 peptide cap (Fig. 3a).

**Fig. 8:**
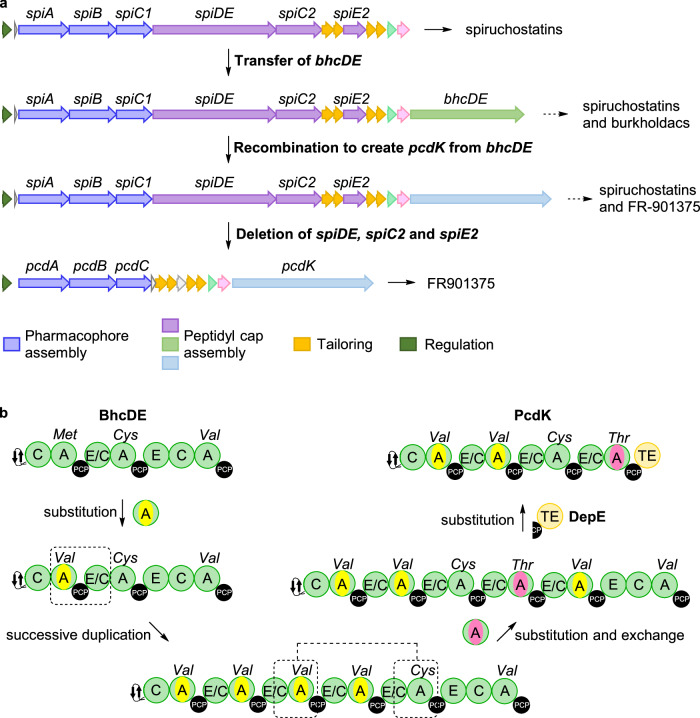
Illustration of the hypothesised evolutionary path from the spiruchostatins BGC to the FR-901375 BGC. **a** A BGC containing NRPS genes encoding the biosynthesis of both products is proposed to be an evolutionary stepping stone. **b** Several homologous recombination and duplication events are hypothesised to convert the burkholdac NRPS into an NRPS capable of assembling the FR-901375 peptidyl cap.

We therefore propose that the evolution of the FR-901375 BGC commenced with horizontal transfer of *bhcDE* from the burkholdac BGC to the C-terminal flank of an ancestral spiruchostatin BGC in *P. chlororaphis* subsp. *piscium*. Based on our observation of productive crosstalk between conserved pharmacophore and variable peptide cap biosynthetic machineries, we hypothesise that the resulting strain would be capable of producing both spiruchostatins and burkholdacs (Fig. 8a). Subsequent substitution of the region encoding the core of the first A domain in BhcDE with a sequence of unknown origin encoding a l-Val-incorporating A domain, is proposed to be the first step in the evolution of an NRPS capable of assembling the peptidyl cap of FR-901375 (Fig. 8b). Three successive duplications of the region encoding the resulting A-PCP-E/C tridomain would create an NRPS with E/C domains in the second, third, fourth, and fifth modules (Fig. 8b). High sequence similarity between putative progeny and progenitor regions in PcdK supports this hypothesis (99% identity over 1048 amino acid residues between the first and second A-PCP-E/C tridomains; 68% identity over 1055 amino acid residues between the second and third A-PCP-E/C tridomains; and 76% identity over 561 amino acids between the first and last A-PCP di-domains). A dip in sequence identity to 34% over 486 amino acids is observed in the region corresponding to the A domain of the third A-PCP-E/C tridomain (Supplementary Fig. S20b). This is consistent with the incorporation of Cys as the third residue in the tetrapeptide cap of FR-901375 (Fig. 3a). Comparison of the sequence in this region to the sequence of the Cys-incorporating A domain in BhcDE shows they share 80% sequence identity over 470 amino acids. We thus propose that recombination between the regions encoding the third and fifth A domain results in exchange (Fig. 8b). A dip in sequence similarity in the core substrate binding region of the A domain between the first and last A-PCP di-domains of PcdK indicates that substitution of this region with a sequence of unknown origin encoding a l-Thr-incorporating A domain is the penultimate step in conversion of BhcDE to PcdK (Supplementary Fig. S22b). The final step requires the fifth and sixth modules to be replaced by a TE domain. Based on the observation that the TE domains in PcdK and the second subunit involved in assembly of the peptidyl cap of romidepsin (Fig. 2e) are 61% identical over 243 amino acids, we propose this occurs via recombination with DepE (Fig. 8b).

Overall, this defines a plausible set of events for the evolution of PcdK from BhcDE, although the order of these may differ from that outlined above. The resulting strain would be expected to be capable of producing both spiruchostatin and FR-901375. Presumably, the ability of *P. chlororaphis* subsp. *piscium* to produce FR-901375 rendered spiruchostatin production dispensable, leading over time to the accumulation of several deletions in *bhcDE*, *bhcE2* and *bhcC2*.

In conclusion, our work delivers deep insight into evolutionary mechanisms underpinning the combinatorial biosynthesis of depsipeptide HDAC inhibitors. This insight could be leveraged to identify the BGC for largazole, which remains elusive. Moreover, it provides a rational basis for developing approaches to the creation of analogues of depsipeptide HDAC inhibitors and other hybrid polyketide-nonribosomal peptides via evolution-guided biosynthetic engineering.

## Methods

### Software implementation and availability

The clusterTools toolkit is implemented in Python 3 and is available under the AGPL. A graphical user interface to access some of the core clusterTools functions is implemented using PyQt5 and is available for Windows, Macintosh and Linux. A clusterTools database constructed from MiBIG entries, and a precomputed clusterTools search of the antiSMASH database with NRPS and PKS modules is also provided. Source code for all the tools described is available at https://www.github.com/emzodls/clusterArch.

### clusterTools searching for putative HDACis and bioinformatics analysis

A clusterTools database was constructed using the genomes in the antiSMASH v 2.0 database using the ncbiGenomeFastaParser function in the clusterTools python toolkit^40,52^. Hidden Markov model (HMM) searches were run on this database with the antiSMASH 5 nrpspksdomains HMMs and the previously reported βHD domain HMM^25,53^. A clusterTools search was conducted with the search terms specified in Table 1 using the processSearchListHmmParser function. DNA sequences containing a 100 kb window centred around the results of the hits of the search were downloaded using the fetchGbksWithAcc function and run through the antiSMASH v5 pipeline.

### Metabolite production, extraction and UHPLC-ESI-Q-TOF-MS analysis

*Pseudomonas chlororaphis* subsp. *piscium* DSM 21509 was first inoculated in 5 ml of Luria-Bertani (LB) medium at 30 °C shaker (180 rpm) overnight. About 1 ml of the overnight culture was inoculated in 50 ml LB medium supplemented with 0.5% (w/v) HP-20 and XAD-16 resins at 30 °C on a shaker (180 rpm) for 72 h. After growing, the cells and resins were harvested by centrifuging (4000×*g* for 15 min) and then freeze-dried. The dried cells and resins were extracted with 5 ml ethyl acetate, and the supernatant was transferred and dried. The crude extract was then dissolved in 1 ml methanol and filtered using a 0.2-μm nylon centrifuge filter (Thermo Scientific). The extracted metabolites were analysed along with a synthetic authentic standard of FR903175 kindly provided by Dr Tadashi Katoh (Tohoku Medical and Pharmaceutical University, Japan) using a Dionex UltiMate 3000 UHPLC connected to a Zorbax Eclipse Plus column (C18, 100 Å, ~2.1 mm, 1.8 μm) coupled to a Bruker MaXis IMPACT ESI-Q-TOF mass spectrometer. A gradient elution of 5 to 100% acetonitrile containing 0.1% formic acid at flow rate of 0.2 ml/min over 35 min was performed. The mass spectrometer was operated in positive-ion mode with a scan range of 50–3000 m/z. Source conditions were: end-plate offset at −500 V; capillary at −4500 V; nebuliser gas (N2) at 1.6 bar; dry gas (N2) at 8 L min^−1^; dry temperature at 180 °C. Ion transfer conditions were: ion funnel RF at 200 Vpp; multiple RF at 200 Vpp; quadrupole low mass at 55 m/z; collision energy at 5.0 eV; collision RF at 600 Vpp; ion cooler RF at 50–350 Vpp; transfer time at 121 s; pre-pulse storage time at 1 s. Calibration was performed with 1 mM sodium formate through a loop injection of 20 μL at the start of each run. For comparison with the synthetic standard of FR910375, culture extract was mixed with 25 μg/ml standard at a volume ratio of 1:1.

### In-frame deletion of *pcdK* and βHD-encoding DNA region in *Pseudomonas chlororaphis* subsp. *piscium* DSM 21509

For in-frame deletion of *pcdK*, two ~1000 bp DNA fragments that flank *pcdK* were amplified by PCR using primers listed in Table S5. The fragments were assembled into the shuttle vector pK18mobsacB (digested with *Xba*I and *Hin*dIII) by GeneArt Cloning (GeneArt™ Seamless Cloning and Assembly Kit, Thermo Fisher Scientific) in *E. coli* S17-1 (*λ pir*) to give the deletion construct pK18mobsacB-*pcdK*, which was verified by sequencing and mobilised from *E. coli* S17-1 (*λ pir*) into *P. chlororaphis* subsp. *piscium* DSM 21509 by bi-parental mating. Overnight cultures of S17 cells containing the deletion plasmid (5 mL LB, 50 mg/mL kanamycin) and *P. chlororaphis* subsp. *piscium* DSM 21509 (5 mL LB) were grown at 30 °C for 16 h, centrifuged and resuspended in LB media (5 mL) to wash. Washing was repeated once more, and resultant solutions were mixed and 100 μL was plated onto a sterile nitrocellulose filter placed onto an LBA plate. The plate was incubated overnight at 30 °C. Cells were washed from the filter using 0.9% NaCl solution (1 mL), and serial dilutions were plated onto LBA plates with double selection (50 mg/mL kanamycin, 100 mg/mL ampicillin) and grown at 30 °C for 72 h. The exconjugant colonies were selected and confirmed as single cross-over mutants by colony PCR using checking primers. The confirmed single cross-over mutant was then grown on LBA plates containing 15% sucrose, and colonies were picked and screened for double cross-over mutants by colony PCR using the checking primers to obtain a 503 bp DNA fragment. To finally confirm the removal of the gene deletion plasmid, the double cross-over mutant candidates were grown in LB (5 mL and 50 mg/ml kanamycin) to check the loss of kanamycin resistance.

Deletion of the βHD-encoding DNA region following the similar procedures as above, apart from the use of sequencing instead of colony PCR for the screening and confirmation of the double cross-over mutant.

### Synthesis of *N*-hexanoyl-amino acid standards

To synthesise *N*-Hexanoyl-l-valine (**15**), hexanoyl chloride (1.03 mg, 7.70 mmol, 1.2 eq) was added dropwise to a stirred solution of l-valine (750 mg, 6.40 mmol, and 1 eq) in aqueous sodium hydroxide (0.5 M, 15%) at 0 °C. The solution was allowed to warm to room temperature and stirred for 16 h. Aqueous hydrochloric acid (20%) was added dropwise until the mixture reached pH 2, and the organic layer was extracted with DCM (3 × 40 mL). Combined organic layers were washed with brine solution (50 mL), dried over MgSO_4_, filtered, and concentrated under reduced pressure to give a white solid (1.125 g, 5.2 mmol, 81.6%). NMR spectra were consistent with reported spectra (Supplementary Fig. 21)^54^.

δ_H_ (400 MHz, CDCl_3_) 4.57–4.64 (m, 1H, COC**H**NH), 2.31-2.37 (t, *J* = 3.5 Hz, 2H, COC**H**_**2**_CH_2_)_,_ 2.18–2.30 (m, 1H, C**H**(CH_3_)_2_), 1.57–1.69 (m, 2H, COCH_2_C**H**_**2**_CH_2_), 1.24–1.37 (m, 4H, CH_2_C**H**_**2**_C**H**_**2**_CH_3_), 0.85–1.00 (m, H9, C**H**_**3**_)

δ_C_ (100 MHz, CDCl_3_) 176.2 (COOH), 174.1 (NHCO), 56.9 (CO**C**HNH), 36.6 (CO**C**H_2_), 31.2 (**C**H_2_CH_2_CH_3_), 31.0 (**C**H(CH_3_)_2_), 25.4 (COCH_2_**C**H_2_), 19.0 ((CH**C**H_3_)_2_), 13.9 (CH_2_**C**H_3_)

HR-MS (*m/z*): [C_11_H_22_NO_3_]+ calculated 216.1594, found 216.1604.

*N*-Hexanoyl-l-methionine (**16**) was synthesised using the same procedure as for *N*-hexanoyl-l-valine, using l-methionine (950 mg, 6.40 mmol, 1 eq) to give a colourless oil (1.18 g, 4.77 mmol, 75%).

δ_H_ (400 MHz, CDCl_3_) (Supplementary Fig. 22) 4.73-4.81 (quar, *J* = 5 Hz, 1H, COC**H**NH), 2.55-2.62 (t, *J* = 6.5 Hz, 2H, C**H**_**2**_SCH_3_), 2.19–2.31 (m, 3H, SC**H**_**3**_), 2.02–2.15 (m, 6H, C**H**_**2**_CH_2_SCH_3_ & NHCOC**H**_**2**_), 1.61–1.70 (quin, *J* = 7.5 Hz, 2H, COCH_2_C**H**_**2**_**)**, 1.25–1.40 (m, 4H, COCH_2_CH_2_C**H**_**2**_C**H**_**2**_), 0.87–0.97 (m, 3H, COCH_2_CH_2_CH_2_CH_2_C**H**_**3**_)

δ_C_ (100 MHz, CDCl_3_) 176.3 (**C**OOH), 174.1 (NH**C**O), 51.7 (CO**C**HNH), 36.5 (CO**C**H_2_), 31.3 (COCH_2_CH_2_**C**H_2_), 31.2 (**C**H_2_CH_2_S), 30.0 (**C**H_2_S), 25.3(COCH_2_**C**H_2_), 22.3 (**C**H_2_CH_3_), 15.4 (S**C**H_3_), 13.9 (CH_2_**C**H_3_)

HR-MS (*m/z*): [C_11_H_22_NO_3_S]^+^ calculated 248.1315, found 248.1325. (Supplementary Fig. 23)

### Cloning and mutagenesis of ACP-SLiM di-domains and βHD-C-A-PCP tetra-domains overexpression constructs

ACP-SLiM di-domain, βHD-C-A-PCP tetra-domain and βHD-C di-domain encoding DNA region were cloned from their respective gDNA into pHis8_G2K vectors using restriction digestion (Table S5) and ligation. Deletion of docking domain regions was achieved using a Q5 site-directed mutagenesis kit (NEB). Primers for cloning and mutagenesis are provided in Table S5. Plasmids were miniprepped using a plasmid miniprep kit (Thermo Fisher Scientific) and sequenced by Sanger sequencing to confirm their identity.

### Protein overproduction and purification

Plasmids were transformed into chemically competent BL21 (DE3) Star cells (Invitrogen) and seeded into LB media with kanamycin (25 μg/mL). Seed cultures were used to inoculate LB medium (1 L) supplemented with kanamycin (25 μg/mL), and cultures were grown at 37 °C to 0.6 OD_600_. Protein overexpression was induced with IPTG (0.25 mM), and cultures were shaken overnight at 15 °C. Cells were harvested by centrifugation (4000×*g*, 20 min), resuspended in loading buffer (20 mM imidazole, 20 mM Tris base, 100 mM NaCl, pH 8.0), lysed using a cell disruptor (Constant Systems) and centrifuged (37,000×*g*, 40 min). Proteins were purified from the lysate by nickel affinity chromatography, eluting with stepwise increases of imidazole concentration. Purified proteins were initially characterised by SDS-PAGE. Upon purification, proteins were buffer exchanged into storage buffer (20 mM Tris base, 100 mM NaCl, pH 8.0) using an appropriate molecular weight cut-off concentrator (Vivaspin), aliquoted, and flash frozen in liquid nitrogen and stored at −80 °C.

### In vitro reconstitution of amide bond formation across the PKS-NRPS interface

*Apo-*carrier proteins (200 μM) were incubated with Sfp (2 μM), MgCl_2_ (10 mM) and hexanoyl-CoA (600 μM) in buffer (20 mM Tris base, 300 mM NaCl, 50 mM HEPES, pH 7.5) for 60 min. *apo-* βHD-C-A-PCPs (200 μM) were incubated with Sfp (2 μM), MgCl_2_ (10 mM) and coenzyme A (600 μM), ATP (1 mM) and either l-valine or l-methionine (1 mM) in buffer (20 mM Tris base, 300 mM NaCl, 50 mM HEPES, pH 7.5) for 60 min. Loaded proteins were mixed in equal volumes and incubated at room temperature for 2 h before quenching with the addition of 0.1% formic acid. For MS analysis, reaction mixtures were buffer exchanged into 0.1% formic acid in H_2_O using an appropriate molecular weight cut-off concentrator (Vivaspin). For enzymatic offloading, the type II TE domain Bamb5926 (10 μM) was added to each reaction mixture and left for 30 min. Proteins were precipitated by the addition of 1% formic acid, followed by the addition of MeOH (2.5x reaction volume). Supernatant was extracted and submitted for LC-MS analysis.

To enhance the detection of mutation-induced effects in the PcdC ACP-SLiM on the condensation reaction, the condensation reaction time was reduced to 20 min.

### UHPLC-ESI–Q-TOF–MS analysis of intact proteins

Intact protein mass spectrometry was undertaken via UHPLC-ESI-Q-TOF-MS using a Bruker MaXis II coupled to Dionex Ultimate 3000 HPLC fitted with an Avantor ACE C4-300 reverse phase column (5 μM, 2.1 × 100 mm, 30 °C). The LC-column was eluted with a linear gradient of 5–100% acetonitrile in water over 30 min, each solvent containing 0.1% formic acid. The mass spectrometer was operated in positive mode with a mass range of 200–3000 m/z. The following source conditions were used for all experiments: end-plate offset −500 V, capillary −4500 V, with 1.4 bar N_2_ nebuliser gas, dry gas N_2_ at 9.0 L/min, 200 °C. Ion transfer conditions used were: ion funnel RF at 400 Vpp, multiple RF at 200 Vpp, quadrupole low mass at 200 m/z, collision energy at 8.0 eV, collision RF at 2000 Vpp, transfer time 110 μs, pre-pulse storage time 10 μs. Spectra were processed in Bruker Compass DataAnalysis 4.1 using standard deconvolution algorithms.

### UHPLC-ESI–Q-TOF–MS analysis of thioesterase off-loaded products and small molecules

High resolution UHPLC-ESI-Q-TOF-MS analyses of small molecules were performed using a Dionex UltiMate 3000 UHPLC connected to a Zorbax Eclipse Plus C18 column (100 × 2.1 mm, 1.8 μm) coupled to a Bruker Compact mass spectrometer. The mobile phases was water and acetonitrile, each supplemented with 0.1% formic acid with a flow rate of 0.2 mL/min. The gradient profile was as follows: 0–5 mins 5% acetonitrile; 5–17 min 5–100% acetonitrile; 17–22 min 100% acetonitrile; 22–25 min 100–5% acetonitrile; 25–34 min 5% acetonitrile. The mass spectrometer was operated in positive-ion mode with a scan range of 50–3000 m/z. Source conditions were: end-plate offset at −500 V, capillary at −4500 V, nebuliser gas (N_2_) at 1.6 bar, dry gas (N_2_) at 81 min^−1^ and dry temperature at 180 °C. Ion transfer conditions were: ion funnel radio frequency (RF) at 200 Vpp, multiple RF at 200 Vpp, quadrupole low mass at 55 *m/z*, collision energy at 5.0 eV, collision RF at 600 Vpp, ion cooler RF at 50–350 Vpp, transfer time at 121 μs and pre-pulse storage time at 1 μs. Calibration was performed with 1 mM sodium formate through a loop injection of 15 μL at the start of each run. Spectra were processed using Bruker Compass DataAnalysis 4.1.

### Carbene footprinting

PcdC ACP-SLiM di-domain and PcdK βHD-C di-domain were overexpressed and purified as previously described, followed by His-tag cleavage by addition of thrombin and incubation overnight at 4 °C. Cleaved protein solutions were further purified by size exclusion chromatography using a 200 pg HiLoad Superdex 16/600 column connected to an AKTA PURE system (GE Healthcare). PcdC ACP-SLiM was converted to holo-form using the previously described loading assay, and loading enzymes were removed by incubation with nickel sepharose beads for 10 min, followed by concentration using a 10 kDa cutoff filter. Solutions were prepared of the holo-PcdC ACP-SLiM di-domain (100 μM, 18 μL), PcdK βHD-C di-domain (50 μM, 18 μL), and a mixture of both proteins at a 2:1 ratio (100 μM:50 μM, 18 μL). Proteins were mixed with sodium 4-(3-(trifluoromethyl)-3H-diazirin-3-yl)benzoate labelling agent (20 mM, 2 μL) in storage buffer solution. This was left to equilibrate at room temperature for 5 min, aliquoted into 6 μL aliquots in crystal-clear vials and snap-frozen in liquid nitrogen. Samples were irradiated at 347 nm (30 s, 1 kHz, pulse energy 130 μJ) with a Nd:YLF laser (Spetra-Physics) for 30 s. Samples were then reduced with DTT solution (10 mM in 10 mM ammonium bicarbonate), alkylated with iodoacetamide (55 mM in 10 mM ammonium bicarbonate) and digested with Glu (1 μL of 0.04 mg/mL stock in water) and AspN (1 μL of 0.04 mg/mL stock in water) overnight at 37 °C. Peptides were analysed by UHPLC-ESI-Q-TOF-MS after 1:5 dilution in water.

LC-MS analysis was carried out using Bruker DataAnalysis software. Each labelled and unlabelled peptide in each sample was identified by extracted ion chromatograms (±0.02 *m/z*) and confirmation of expected charge state. Fractional modification of each peptide was calculated as the labelled peak intensity over the sum of labelled and unlabelled intensities. Labelling differences were considered to be significant if the difference was greater than the sum of two standard deviations of both the labelled and unlabelled peptide fractional modification across three repeats.

### AlphaFold modelling of the PcdC:PcdK interface

Monomer and multimer models of the PcdC ACP-SLiM di-domain and PcdK βHD-C di-domain, individually and in complex, were generated using a local installation of AlphaFold 2.1 with the run relaxed parameter selected. Five ranked relaxed models were produced and visualised in ChimeraX, and the most representative model was selected manually in each case for further comparison and analysis.

### Molecular dynamics simulations of the PcdC:PcdK interface

The AlphaFold 2.1 model of the complex between the PcdC ACP-SLiM and PcdK βHD-C di-domains was prepared using the H + + webserver to add hydrogens and calculate the charge of histidine residues at pH 7.0 in a solvent box of 10 Å^3^ ^55^. A modified serine residue with Ppant and tethered hexanoyl substrate was generated in Chimera, and an appropriate forcefield was produced in ANTECHAMBER^56^. Substrate-appended serine was positioned in place of the functionalised serine of the ACP domain in each model manually, followed by two rounds of steepest descent structure minimisation in Chimera 1.16.1. Na^+^ ions were added to neutralise the overall charge, and additional salt ions were added to simulate a 150 mM salt concentration. The model was solvated with OPCBOX water, such that no atom belonging to the complex was less than 10 Å from any box edge, by TLEaP, and initial MD heating, equilibration and production steps conducted using Amber version 20 with Amber ff19SB force field and OPC water model^57^. The solvated protein system was heated at constant volume, using Langevin dynamics, to 300 K over the course of 50 ps^58^. The system was then equilibrated for 500 ps at constant pressure prior to the production simulation. To do this, the solvated system was heated to 300 K over 0.1 ns in a canonical ensemble (NVT) simulation and then equilibrated for 2 ns by performing isothermal–isobaric (NPT) simulations using a Berendsen barostat. Protein bonds involving hydrogen atoms were treated with the SHAKE algorithm, and the smooth Particle-Mesh Ewald method was employed for long-range electrostatics^59^. Equilibration was confirmed by plotting the total energy of the system during the equilibration phase (Supplementary Fig. 24). Three independent repeats of classical MD simulations were run for 100 ns with a timestep of 0.002 ps to stabilise each system and generate parameters for longer accelerated MD (aMD) simulations. The classical MD simulations were then run for a further 400 ns.

Accelerated molecular dynamics simulations were then performed using CUDA-accelerated Amber version 20, using the results of the classical MD simulations as input. Each simulation was run for 500 ns using a 0.002 ps timestep, recording frames every 50 ps for analysis, using the Amber ff19SB force field and OPC water model. The aMD modification of the potential were defined as:

1 $${V\left(r\right)}^{*}=V\left(r\right)+\triangle V\left(r\right)$$

2 $$\triangle V\left(r\right)=\frac{{({E}_{P}-V\left(r\right))}^{2}}{({\alpha }_{P}+{E}_{P}-V\left(r\right))}+\frac{{({E}_{D}-V\left(r\right))}^{2}}{({\alpha }_{D}+{E}_{D}-{V}_{D}\left(r\right))}$$

where *V*(r) is the normal potential and *V*_D_(r) is the normal torsion potential, with *E*_P,_ a_P,_ E_D,_ and a_D_ calculated separated for each repeat (estimated from the classical MD simulations, see Amber manual). No significant changes in structure were observed during aMD simulations (Supplementary Fig. 25).

Trajectories and topologies were stripped of extraneous water molecules to reduce file size, and RMSD and distance analysis was conducted in CPPTRAJ to aid subsequent visualisation in ChimeraX.

### Reporting summary

Further information on research design is available in the Nature Portfolio Reporting Summary linked to this article.

## Supplementary information

Supplementary Information

Reporting Summary

Transparent Peer Review file

## Source data

Source Data

## Data Availability

The data that support the findings of this study are provided within the manuscript and supplementary information. Source data underlying Supplementary Figs. 6, 13, 14, 15, 20 and 24 are available from the Zenodo repository under the accession code 18682377 [10.5281/zenodo.18682376], the Source Data file and the corresponding authors upon request. The NMR data, MD trajectories, forcefields and AlphaFold models are also available from the Zenodo repository. Source data are provided with this paper.

## Code availability

Source code for the clusterTools toolkit described is available at [https://www.github.com/emzodls/clusterArch]. Stand-alone versions of clusterTools are also available for Macintosh, Windows and Linux upon registration (https://goo.gl/forms/QRKTkpqiA0g31IWp1).
