# Supplementary material for: Molecular basis for depsipeptide HDAC inhibitor combinatorial biosynthesis
Source: Nat Commun. 2026 Jul 1;17:5508. doi: 10.1038/s41467-026-74383-4 (PMC13324335; doi:10.1038/s41467-026-74383-4)
Supplement: Supplementary file 1 — Supplementary Information [file 41467_2026_74383_MOESM1_ESM.pdf]

Supplementary Information

Supplementary Table 1: ClusterTools search terms used to identify the FR901375 BGC

Feature	ClusterTools search terms
1. Gene encoding NRPS for initiating conserved pharmacophore biosynthesis	Acyl CoA-ligase domain, Cy domain, and AMP-binding domain
2. Gene encoding PKS for assembly of conserved pharmacophore	PKS_KS domain and (NOT PKS_AT domain) and ACP domain
3. Gene encoding NRPS with N-terminal βHD domain for variable peptide cap assembly	βHD domain, and (Condensation_$^{D}C_{L}$, or Condensation_$^{L}C_{L}$, or Condensation_Dual, or Condensation_Starter, or Cglyc) domain, and AMP-binding domain

Supplementary Table 2: Putative bicyclic depsipeptide HDAC inhibitor BGCs identified using clusterTools

Accession no.	Strain	Start	End	Pharmacophore NRPS hit	Pharmacophore PKS hit	Variable peptide cap NRPS hit
NZ_LDUI01000030.1	*Chromobacterium* sp. LK1	169382	197567	WP_082151066	WP_048410322;WP_048410323	WP_048410324;WP_048410380
NZ_CP013381.1	*Burkholderia* sp. Bp5365 MSMB43	222054	250290	WP_006029651	WP_006029652;WP_043283373	WP_082262297;WP_006029657
NZ_JONK01000024.1	*Chromobacterium haemolyticum* DSM 19808	2	20875	WP_081862670	WP_043638441;WP_043638445	BR71_RS13775
NZ_LDUR01000004.1	*Chromobacterium* sp. LK11	210565	226850	WP_082158729	WP_048412589; WP_048412590	VK98_RS02965
NZ_CP013458.1	*Burkholderia* sp. MSMB617	1468457	1492611	WP_082759493	WP_038744878;WP_060356659	WP_060356658
NZ_LOWB01000002.1	*Burkholderia* sp. TSV86	6225	30340	WP_082709718	WP_059568894;WP_059568895	WP_059568896
NZ_CP013425.1	*Burkholderia* sp. MSMB0852	1434354	1458478	WP_082745045	WP_059643860;WP_059643862	WP_059643857
NZ_LNJR01000001.1	*Burkholderia* sp. BDU19	819831	843985	WP_082761353	WP_060356659;WP_060819971	WP_060819972
NZ_LOYJ01000089.1	*Burkholderia* sp. MSMB1498	16357	40481	WP_082758240	WP_059670810;WP_059670811	WP_059670809
NZ_CP013407.1	*Burkholderia thailandensis* MSMB59	2038821	2062990	WP_009909391	WP_009909390;WP_009891082	WP_009909386
NZ_CP013418.1	*Burkholderia* sp. MSMB0266	772842	796966	WP_082717419	WP_059582785;WP_059582782	WP_059582790

NZ_JPWT01000047.1	*Burkholderia* sp. ABCPW 111	37178	61332	WP_081989387	WP_052145018;WP_038744878	WP_038744879
NZ_CP013409.1	*Burkholderia thailandensis* 2002721121	1954295	1978463	WP_080554683	WP_019254366	WP_043300534
NZ_LT629761.1	*Pseudomonas chlororaphis* DSM 21509	3817694	3852691	WP_081001480	WP_053278624	WP_081364225

Supplementary Table 3: Proposed functions of proteins encoded by the FR901375 BGC and comparison to proteins encoded by the spiruchostatin BGC

**FR901375 BGC**	**Spiruchostatin BGC**	**Identity/ Similarity (%)**	**Predicted Function**
**Gene/protein**	**Gene/protein**		
pcdA/PcdA	*spiA*/SpiA	86.8/92.2	NRPS (Pcd/Spi module 1)
pcdB/PcdB	*spiB*/SpiB	91.0/94.7	PKS (Pcd/Spi module 2)
pcdC/PcdC	*spiC1*/SpiC	87.9/91.6	PKS (Pcd/Spi module 3)
pcdK/PcdK	-		NRPS (Pcd modules 4-7)
-	*spiDE1*/SpiDE		NRPS (Spi modules 4-5)
-	*spiC2*/SpiC2		PKS (Spi module 7)
pcdE2/PcdE2	*spiE2*/SpiE2	77.3/81.8	NRPS (Spi module 8)
pcdF/PcdF	*spiF*/SpiF	98.6/98.9	FadE2-like acyl-CoA dehydrogenase
pcdG/PcdG	*spiG*/SpiG	89.1/93.5	Phosphotransferase
pcdH/PcdH	*spiH*/SpiH	92.7/95.7	FAD-dependent disulphide oxidoreductae
pcdI/PcdI	*spiI*/SpiI	89.5/92.1	Esterase/Lipase
pcdJ/PcdJ	*spiJ*/SpiJ	91.7/95.8	Type II thioesterase
pcdP/PcdP	*spiP*/SpiP	86.2/90.9	Malonyl-CoA specific acyltrasferase
pcdR/PcdR	*spiR*/SpiR	82.9/90.9	OxyR-type transcriptional regulator

Supplementary Table 4: Predicted substrate specificities of A domains in the NRPS proposed to assemble the variable peptidyl cap of FR901375

Module 4		Module 5		Module 6		Module 7	
residues	closest match	residues	closest match	residues	closest match	residues	closest match
DAWWLGGT	TycC-M4 (L-Val)	DAWWLGGT	TycC-M4 (L-Val)	DLFEMSLI	PchE-M1 (L-Cys)	DFWNIGMI	ApdB-M3 (L-Thr)

The putative specificity conferring residues and the A domain of known function with the closest match for the A domain in each module of the NRPS were extracted using PKS/NRPS Analysis (http://nrps.igs.umaryland.edu). TycC-M4-Val: L-Val-incorporating A domain from the fourth module of tyrocidine A NRPS TycC; PhE-M1-Cys: L-Cys-incorporating A domain from the first module of the pyochelin NRPS PchE; ApdB-M3-Thr: L-Thr-incorporating A domain from the third module of the cyanopeptolin NRPS ApdB.

Supplementary Table 5: Sequences of PCR primer pairs used for generation of plasmids.

Construct (& restriction sites used)	Primers	Template
pET28a-pHis8-G2K	FOR: ATATACCATGAAACACCACCATCATC<br>REV: CTCCTTCTTAAAGTTAAACAAAATTATTTC<br>REV: TGATGATGATGATGATGGTGGTGTTTC	pET28a-pHis8
pET28a-pHis8_ PcdC_ACP-SLiM (NdeI/XhoI)	FOR: ATACATATGCCGATGCGAAGACCCGTATTT<br>REV: ATACTCGAGTCATAGGGTAATTTTCTCTGTGCTAGTA	*Pseudomonas chlororaphis* DSM 21509 gDNA
pET28a-pHis8_ PcdK_βHD-C-A-PCP (NdeI/XhoI)	FOR: ATACATATGCATGCGGTGCCGCT<br>REV: ATACTCGAGTCAGGGGATAAGGTTGTCGGGGA	*Pseudomonas chlororaphis* DSM 21509 gDNA
pET28a-pHis8-G2K_ PcdC_ACP-SLiM	FOR: ATATACCATGAAACACCACCATCATC<br>REV: CTCCTTCTTAAAGTTAAACAAAATTATTTC	pET28a-pHis8_ PcdC_ACP-SLiM
pET28a-pHis8-G2K_ PcdK_βHD-C-A-PCP	FOR: ATATACCATGAAACACCACCATCATC<br>REV: CTCCTTCTTAAAGTTAAACAAAATTATTTC	pET28a-pHis8_ PcdK_βHD-C-A-PCP
pET28a-pHis8-G2K_ PcdK_βHD-C	FOR: ATACATATGAATATCGAGCGGCTCATGACCGAT<br>REV: ATACTCGAGTCACGGGAAATCGCTCTGG	pET28a-pHis8_ PcdK_βHD-C-A-PCP
pET28a-pHis8-G2K_ DepC_ACP-SLiM (NdeI/XhoI)	FOR: ATACATATGAGCGCGCGGACTCTGTCTTT<br>REV: ATACTCGAGTCATAGCGTGATTTCCTCCGTGT	*Chromobacterium violaceum* FERM-BP1968 gDNA
pET28a-pHis8G2K_ DepD_βHD-C-A-PCP (NdeI/HindII)	FOR: ATACATATGATGACCATGGCACGGCTCAT<br>REV: ATAAAGCTTTCACTGCTCCGCCTG<br>REV: ATAGAATTCTCATTGCTCGGCAAGCACC	*Chromobacterium violaceum* FERM-BP1968 gDNA
pET28a-pHis8-G2K-L14Y_BhcC_ACP-SLiM (NdeI/XhoI)	FOR: ATACATATGCGCGCACGCGCCTT<br>REV: ATACTCGAGTCATAGCGTGATTTCCTCCGTCT	*Burkholderia thailandensis* DSM13276 gDNA
pET28a-pHis8 G2K_ BhcDE_βHD-C-A-PCP (NdeI/XhoI)	FOR: ATACATATGAATATCGTTCGGCTCATGGCCGATTT<br>REV: ATACTCGAGTCAGGGCGGCACCGCAAAA	*Burkholderia thailandensis* DSM13276 gDNA
pET28a-pHis8-G2K SpiC1_ACP-SLiM (NdeI/XhoI)	FOR: ATACATATGATGCGAAGACCCGTGTTTGATTT<br>REV: ATACTCGAGTCATAAGGTCATCTTCTCTGTGCTATT	*Pseudomonas sp.* Q71576 gDNA
pET28a-pHis8_ PcdC_ACP(ΔSLiM)	FOR: TGACTCGAGCACCACCAC<br>REV: GCTCACCGACTGGGTAGT	pET28a-pHis8_ PcdC_ACP-SLiM

pET28a-pHis8_ DepC_ACP(ΔSLiM)	FOR: TGACTCGAGCACCACCACCAC	pET28a-pHis8_DepC_ACP-SLiM
pET28a-pHis8_ PcdC_ACP(I50A)	REV: GCCGACGGCCGAGTGTT	pET28a-pHis8_ PcdC_ACP-SLiM
pET28a-pHis8_ BhcC_L14Y_ACP(ΔSLiM)	FOR: TGACTCGAGCACCACCACCACCAC	pET28a-pHis8_ PcdC_BhcC_L14Y_ACP-SLiM
pET28a-pHis8_ PcdC_ACP(R21K)	FOR: CGCTGATGCTAAACGAGCATTGATCG<br>REV: CTGTGCCCACGAAGTTG	pET28a-pHis8_ PcdC_ACP-SLiM
pET28a-pHis8_ PcdC_ACP(E97A)	FOR:GGGCCAACTTgctCGCCGGCTCG<br>REV:ACAATCACTTCAACCACGGTGTTC	pET28a-pHis8_ PcdC_ACP-SLiM
pET28a-pHis8_ PcdC_ACP(V101D)	FOR: GCGCCGGCTCGATACTACCCAGT<br>REV: TCAAGTTGGCCCACAATCACTTC	pET28a-pHis8_ PcdC_ACP-SLiM
pET28a-pHis8_ PcdC_ACP(T102A)	FOR:CCGGCTCGTGgctACCCAGTCGG<br>REV:CGCTCAAGTTGGCCCACAATCACTTC	pET28a-pHis8_ PcdC_ACP-SLiM
pET28a-pHis8-G2K_ PcdK_C-A-PCP (ΔβHD)	FOR: CATGCGGTGCCGCTGCCC	pET28a-pHis8-G2K_ PcdK_βHD-C-A-PCP
	REV: CATCCATGGTATATCTCCTTCTTAAAGTTAAACAAAATTATTTCTAGAGGGG	
	FOR: ATACTCGAGCGGCGCCTGATAACCACGAGTTGCCA	
	REV: ATACTCGAGTCAGGGGATAAGGTTGTCGGGGA	
pK18mobsacB_PcdKΔβHD	5' ARM FOR: ATATCTAGAAGCGGACTCAATGACGTTGGTA	*Pseudomonas chlororaphis* DSM 21509 gDNA
	5' ARM REV: CACCGCATGCATAGGTTGATATCTCCA	
	3' ARM FOR: CAACCTATGCATGCGGTGCCG	
	3' ARM REV: ATAAAGCTTCTCAGCAATAGACTCAATCGGTACG	
	REV: ATACTCGAGCTAGAGCAACACAGACCGCAG	
pK18mobsacB-pcdK	5' ARM FOR: AGCTCGGTACCCGGGGATCCTCTAGAGTTTTTGCAGCGTTTCTG	
	5' ARM REV: CATGATGCCAATCATCTGCAACAATGTTTCTT	
	3' ARM FOR: TGTTGCAGATGATTGGCATCATGCGCTAT	
	3' ARM REV: GTAAAACGACGGCCAGTGCCAAGCTTAACGCCAATTACAGCAAT	

CHECK FOR: GATCGTCGCTCGATAGGGTG
CHECK REV: CCGCAGCAGGATCAATCACA

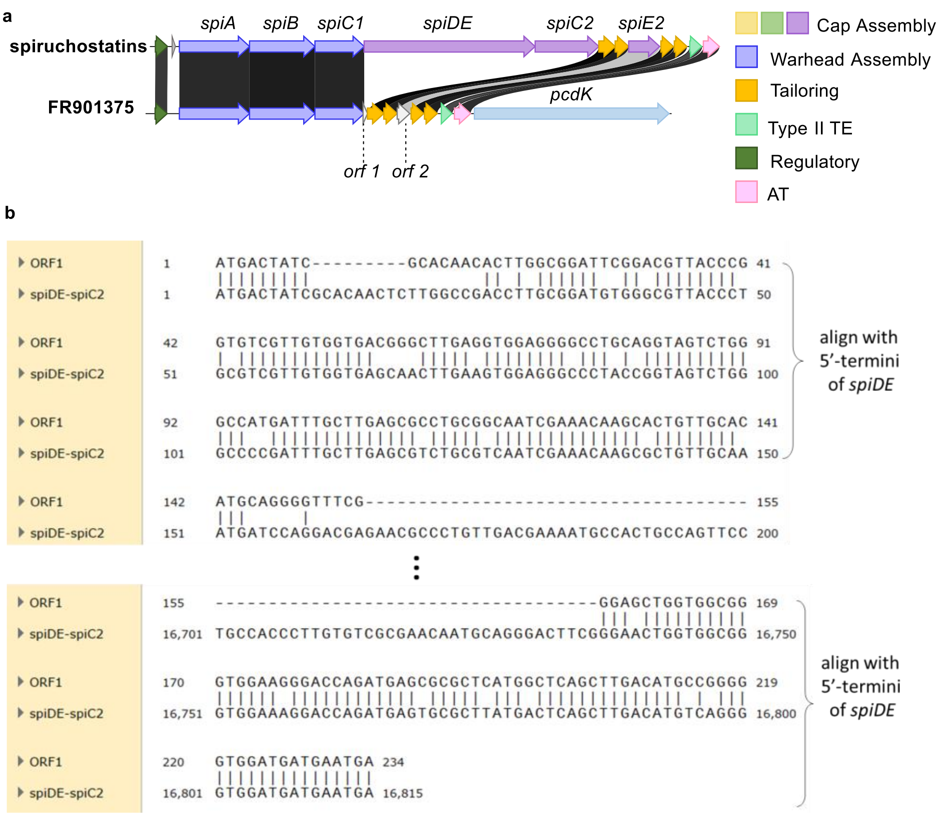
a
spiruchostatins
FR901375
spiA
spiB
spiC1
spiDE
spiC2
spiE2
pcdK
orf 1
orf 2
Cap Assembly
Warhead Assembly
Tailoring
Type II TE
Regulatory
AT
b
ORF1 1 ATGACTATC---------GCACAACACTTGGCGGATTCGGACGTTACCCG 41
spiDE-spiC2 1 ATGACTATCGCACAACTCTTGGCCGACCTTGCGGATGTGGGCGTTACCCT 50
ORF1 42 GTGTCGTTGTGGTGACGGGCTTGAGGTGGAGGGGCCTGCAGGTAGTCTGG 91
spiDE-spiC2 51 GCGTCGTTGTGGTGAGCAACTTGAAGTGGAGGGCCCTACCGGTAGTCTGG 100
ORF1 92 GCCATGATTTGCTTGAGCGCCTGCGGCAATCGAAACAAGCACTGTTGCAC 141
spiDE-spiC2 101 GCCCCGATTTGCTTGAGCGTCTGCGTCAATCGAAACAAGCGCTGTTGCAA 150
ORF1 142 ATGCAGGGGTTTCG------------------------------------ 155
spiDE-spiC2 151 ATGATCCAGGACGAGAACGCCCTGTTGACGAAAATGCCACTGCCAGTTCC 200
align with 5′-termini of spiDE
ORF1 155 -----------------------------------GGAGCTGGTGGCGG 169
spiDE-spiC2 16,701 TGCCACCCTTGTGTCGCGAACAATGCAGGGACTTCGGGAACTGGTGGCGG 16,750
ORF1 170 GTGGAAGGGACCAGATGAGCGCGCTCATGGCTCAGCTTGACATGCCGGGG 219
spiDE-spiC2 16,751 GTGGAAAGGACCAGATGAGTGCGCTTATGACTCAGCTTGACATGTCAGGG 16,800
ORF1 220 GTGGATGATGAATGA 234
spiDE-spiC2 16,801 GTGGATGATGAATGA 16,815
align with 5′-termini of spiDE

c

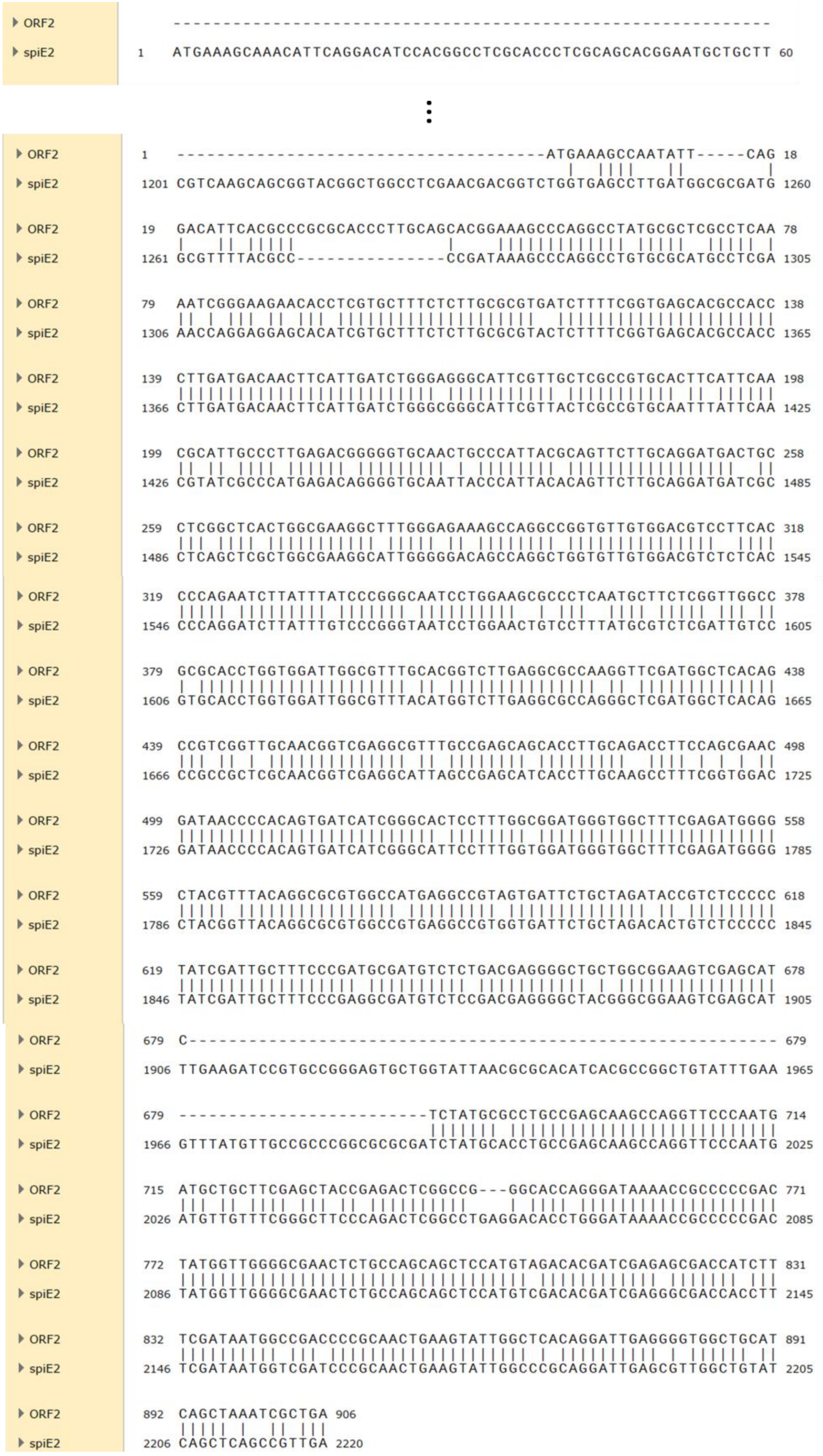

Supplementary Figure 1: Sequence alignment of *orf1* and *orf2* in the FR901375 BGC with the *spiDE* / *spiC2* genes and *spiE2* genes, respectively. a, Organisation of the FR901375 BGC, highlighting the positions of *orf1* and *orf2*. **b,** The 1-155 nt region and 156-234 nt region of *orf1* are highly similar in sequence to the 5′ and 3′ ends of *spiDE* and *spiC2*, respectively. **c,** Alignment of *orf2* with *spiE2*, showing that although they are very similar in sequence, the former contains four multi-base deletions and one insertion, relative to the latter.

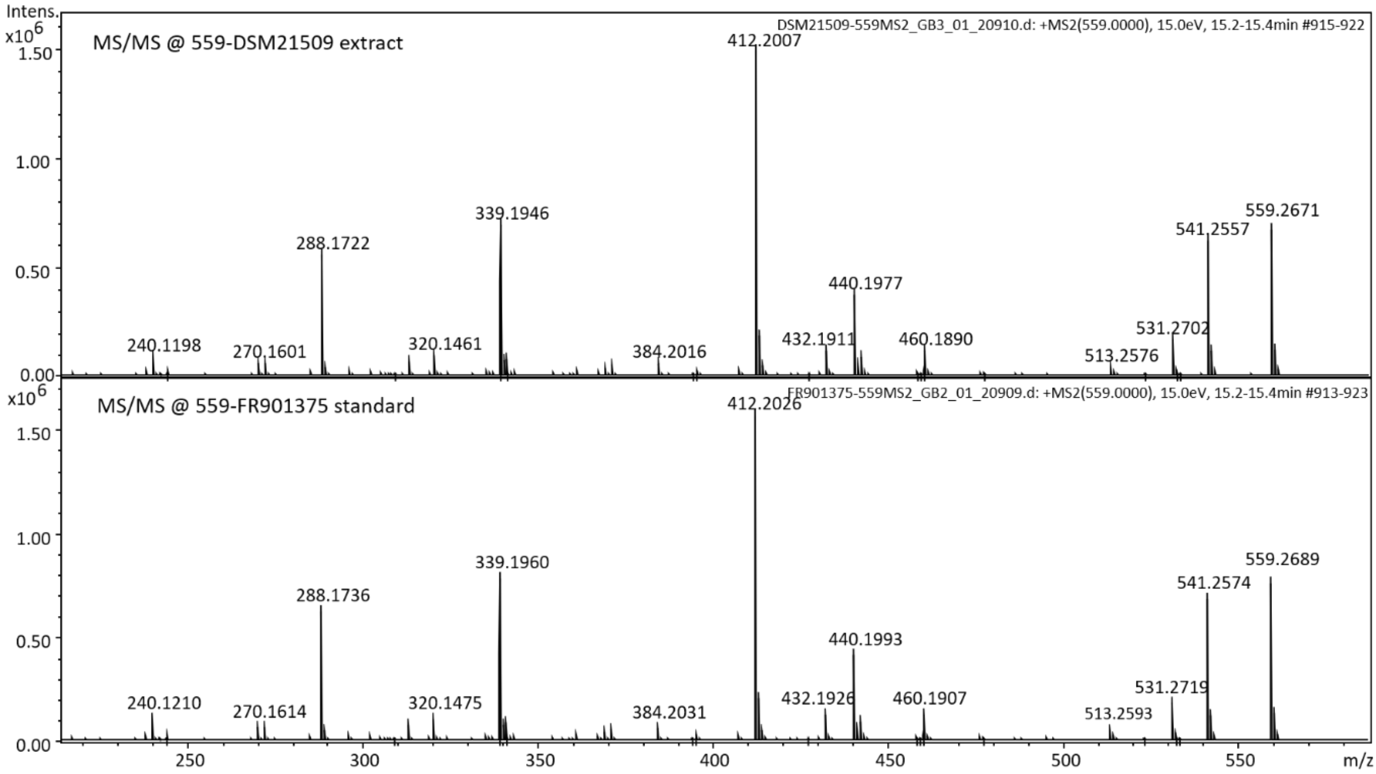

Supplementary Figure 2: UHPLC-ESI-QTOF-MS/MS comparison of FR901375 from the culture extract of *P. chlororaphis* DSM 21509 with a synthetic standard. MS/MS spectra of the species with *m/z* = 559.0 ± 1 Da, corresponding to the $[M+H]^+$ ion of FR901375, in the culture extract (top) and the FR901375 synthetic standard (bottom).

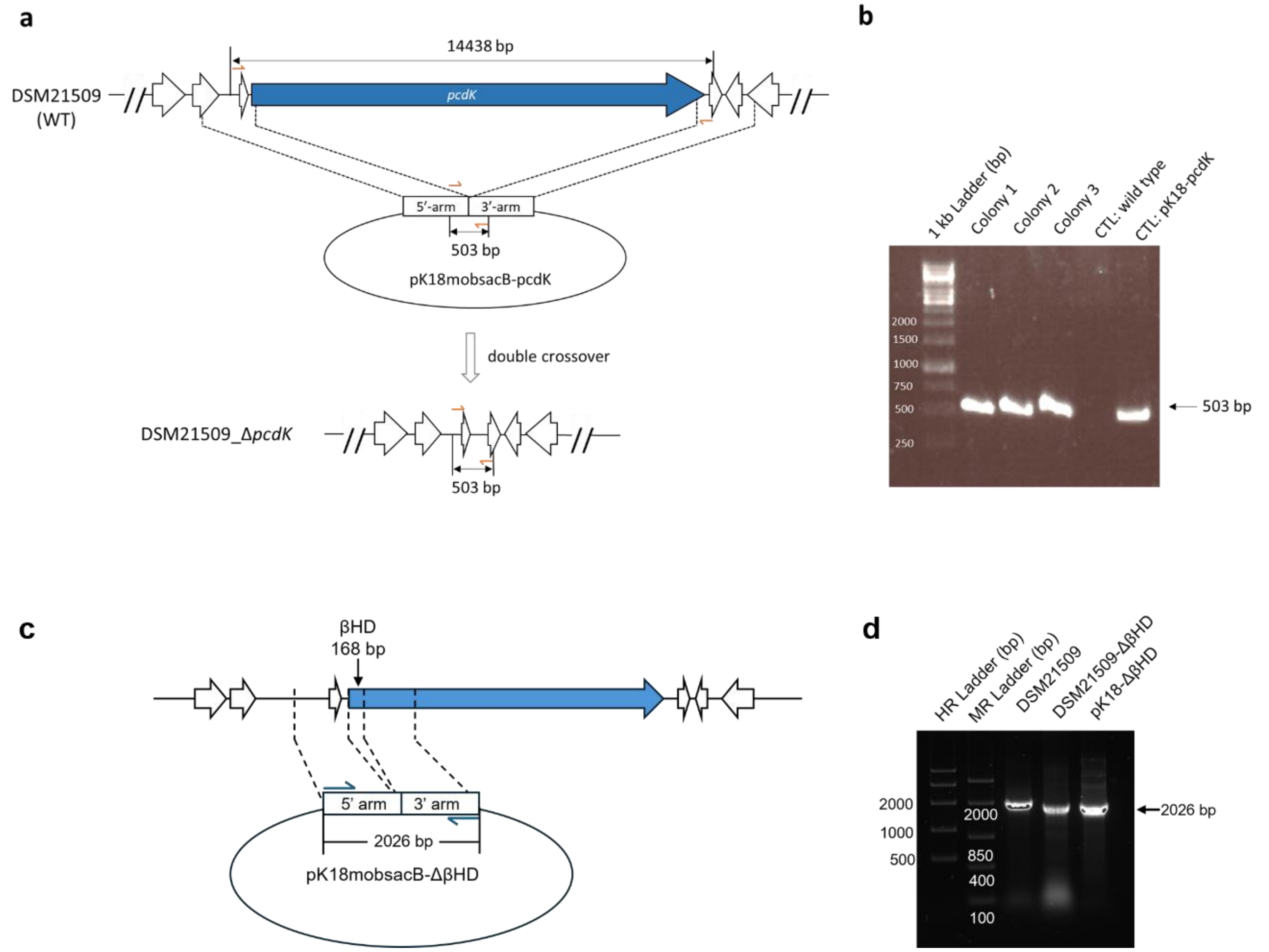

Supplementary Figure 3: Schematic representation of construct design and PCR confirmation for the *pcdK* whole gene and βHD domain encoding in-frame deletions in *P. chlororaphis* subsp. *piscium* DSM21509. a. Construct design for in-frame deletion of the whole *pcdK* gene. **b,** PCR confirmation of the *pcdK* whole gene in-frame deletion. **c,** Construct design for in-frame deletion of the βHD domain-encoding region of *pcdK*. **d,** PCR confirmation of in-frame deletion of the βHD domain-encoding region of *pcdK*.

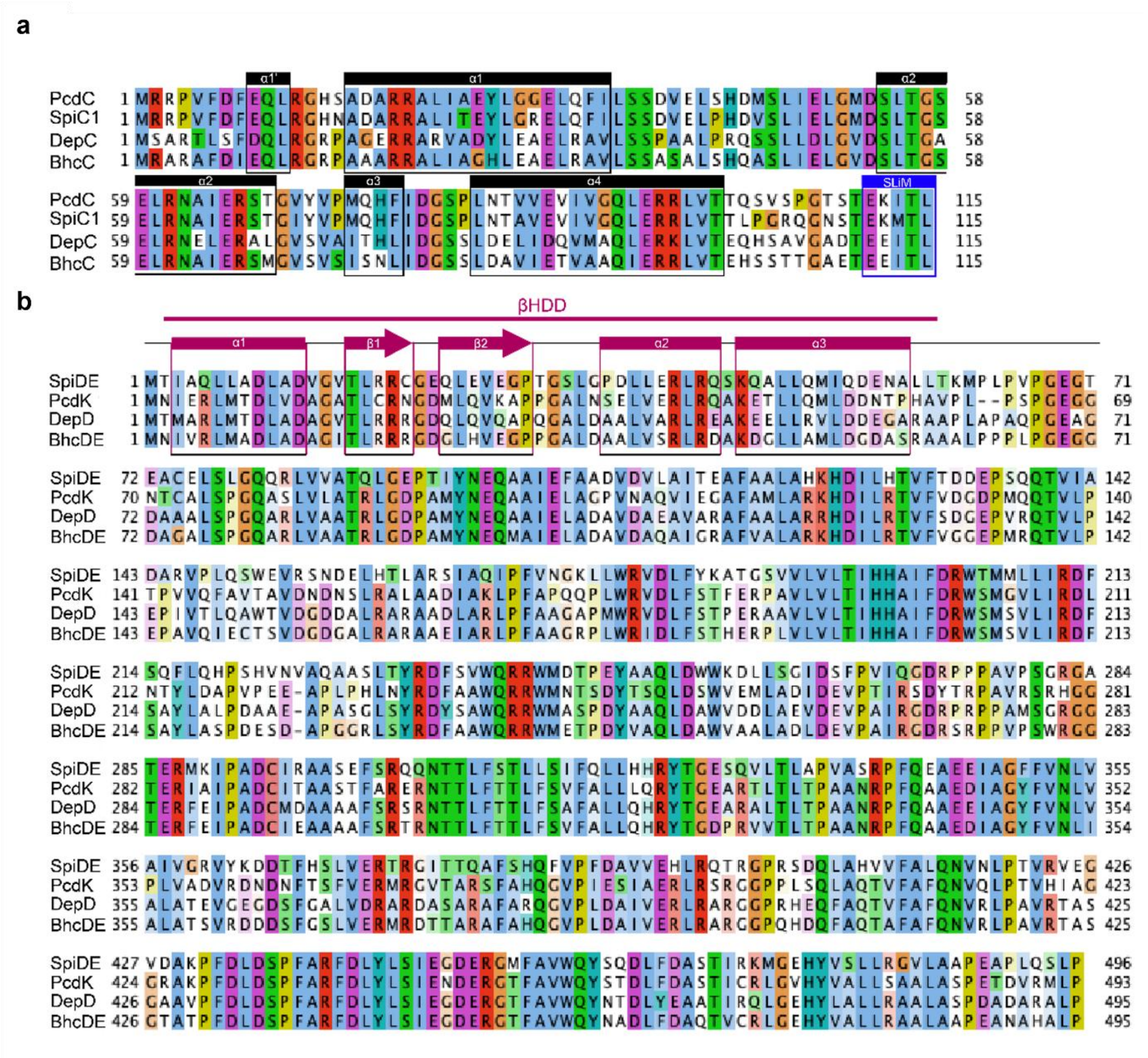

Supplementary Figure 4: Sequence alignments of ACP-SLiM and βHD-C didomains involved in bicyclic depsipeptide HDAC inhibitor biosynthesis. **a,** Sequence alignment of the ACP-SLiM didomains from PcdC, SpiC1, DepC, and BhcC. α-Helical regions are indicated by black boxes, and the SLiM is highlighted by a blue box. **b,** Sequence alignment of the βHD-C didomains from PcdK, SpiDE, DepD, and BhcDE. The region corresponding to the βHD domain is indicated by the pink bar and α-helical / β-sheet secondary structure elements within this are highlighted by pink boxes.

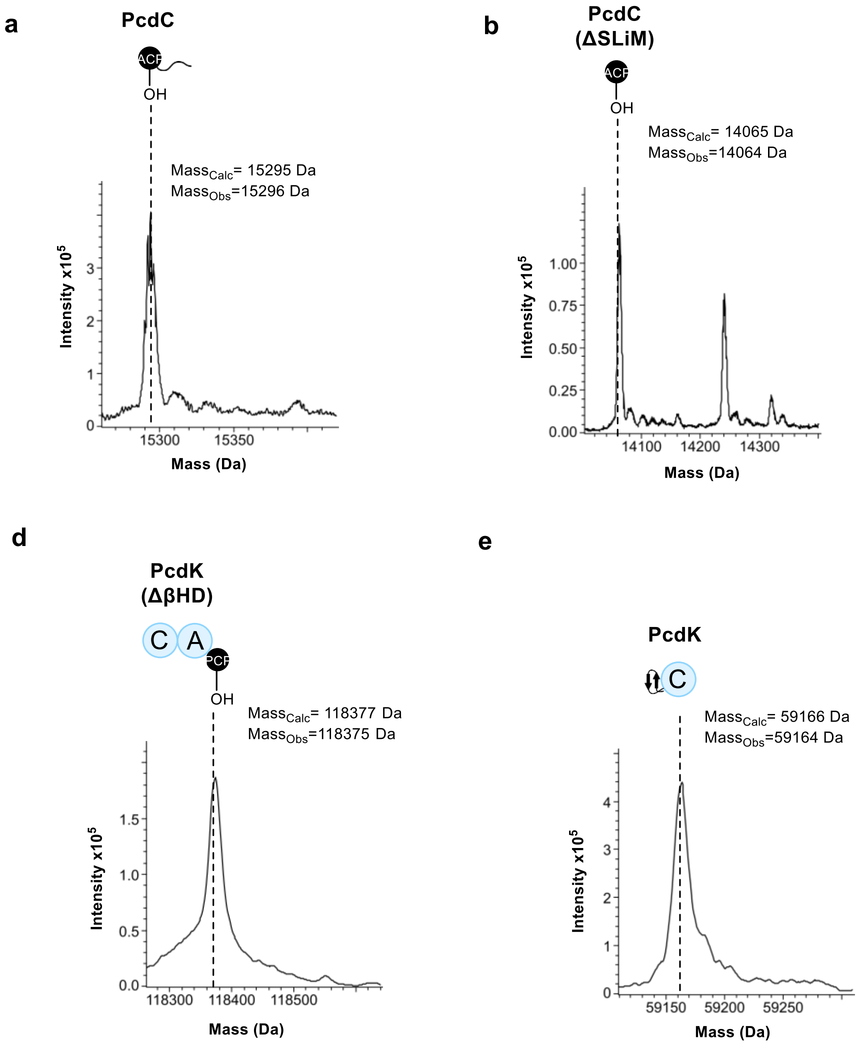

Supplementary Figure 5: Characterisation of wild type and mutant PcdC ACP-SLiM didomain, and βHD-C-A-PCP tetradomain and βHD-C didomains excised from PcdK. a, Intact mass spectrum of the PcdC *apo*-ACP-SLiM didomain. **b,** Intact mass spectrum of the PcdK *apo*-βHD-C-A-PCP tetradomain. **c,** Intact protein mass spectrum of the PcdC *apo*-ACP(ΔSLiM) domain. **d,** Intact protein mass spectrum of the PcdK *apo*-C-A-PCP(ΔβHD) tridomain. **e,** Intact protein mass spectrum of PcdK βHD-C didomain.

a

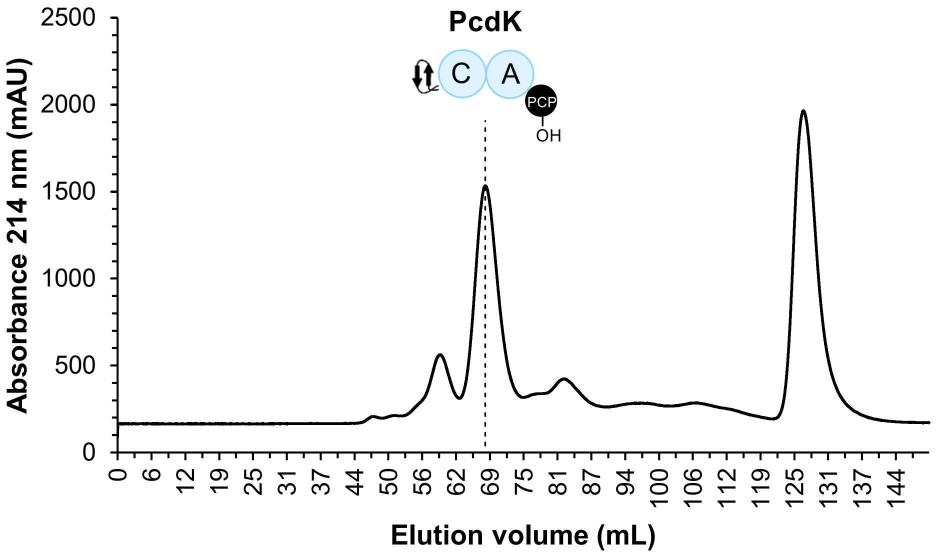

b

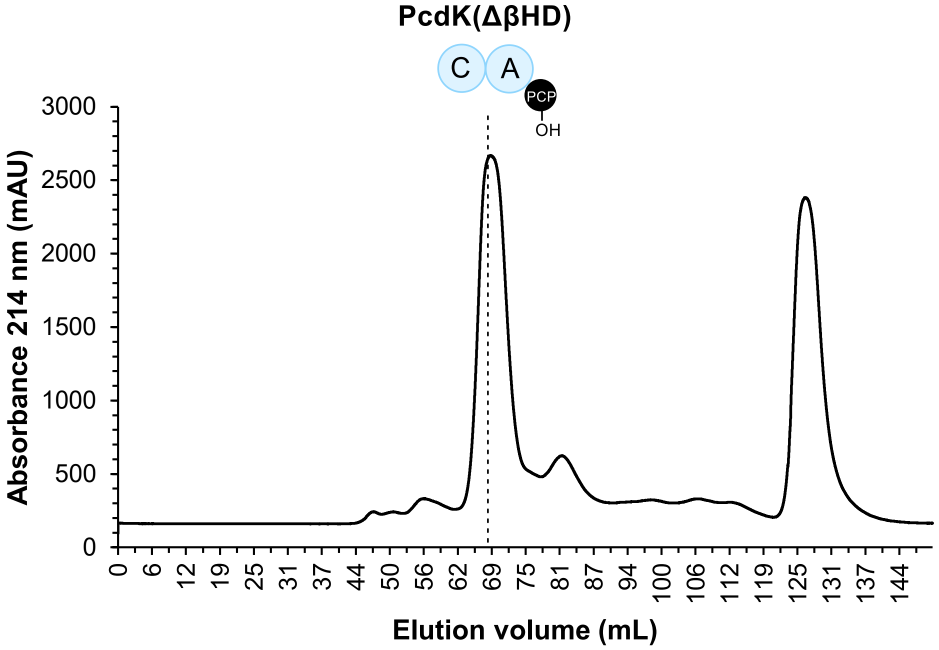

Supplementary Figure 6: Size exclusion chromatography (SEC) of PcdK βHD-C-A-PCP tetradomain, and PcdK C-A-PCP tridomain. a, Chromatogram from SEC of PcdK βHD-C-A-PCP tetradomain. **b,** Chromatogram from SEC of PcdK C-A-PCP tridomain. These analyses show no change in multimerization state between WT and ΔβHD PcdK. Both elute as monomers. Calculations based on a calibration curve gave predicted masses from the elution times as follows: PcdK 159.92 kDa (expected mass: 124.37 kDa), PcdKΔβHD = 149.44 kDa (expected mass: 118.38 kDa). Source data are provided in the Source Data file**.**

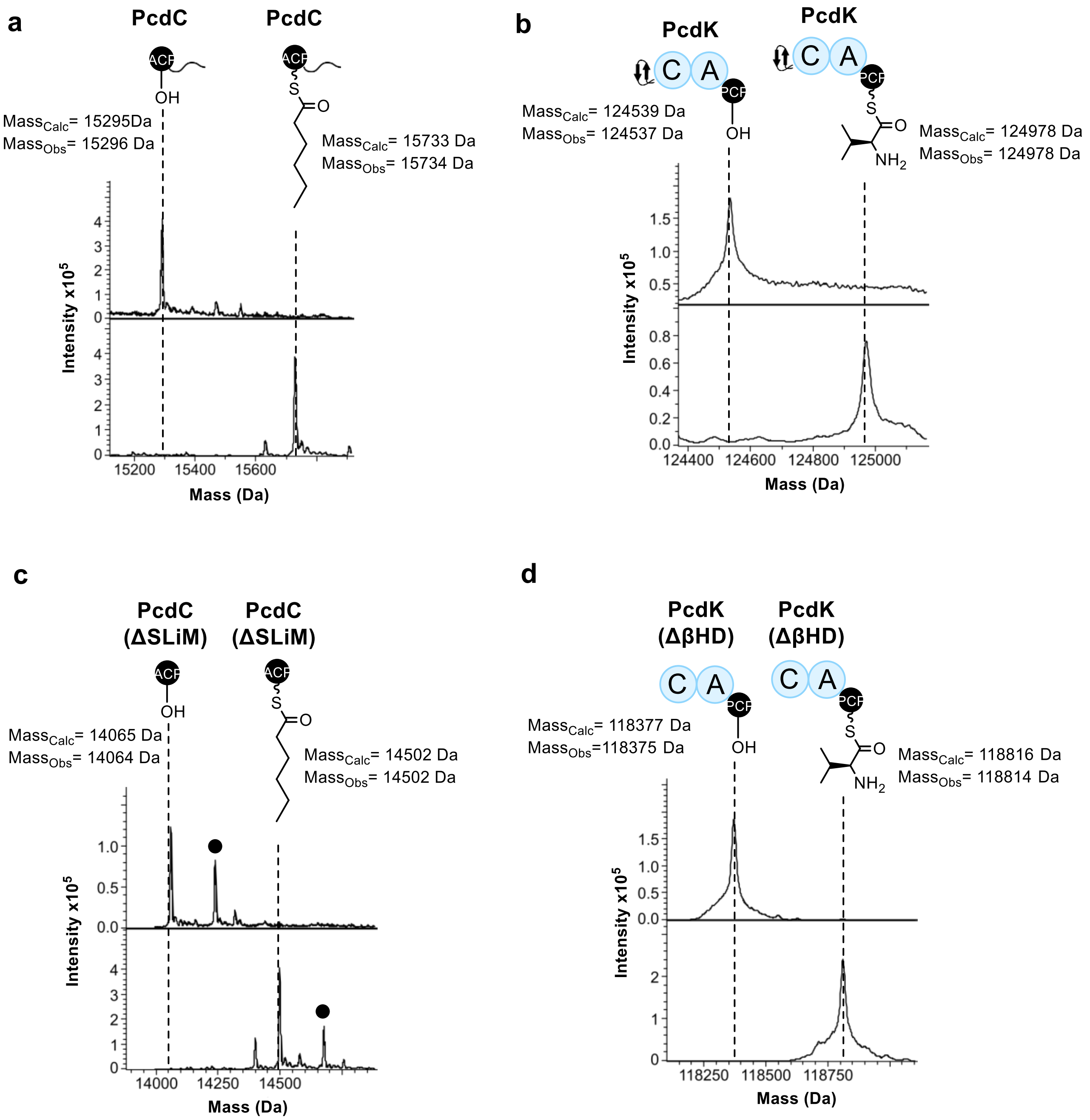

Supplementary Figure 7: Posttranslational phosphopantetheinylation and (amino)acylation of the PcdC *apo*-ACP-SLiM didomain, PcdC *apo*-ACP(ΔSLiM) domain, PcdK *apo*-βHD-C-A-PCP tetradomain and PcdK *apo*-C-A-PCP(ΔβHD) tridomain. a, Intact protein mass spectra of *apo* and hexanoyl-ACP-SLiM didomain from PcdC. **b,** Intact protein mass spectra of *apo* and L-valinyl-βHD-C-A-PCP tetradomain from PcdK. **c,** Intact protein mass spectra of the *apo* and hexanoyl-ACP(ΔSLiM) domain from PcdC. Gluconoylation peak (+178) of both species is denoted by a circle. **d,** Intact protein mass spectra of *apo* and L-valinyl C-A-PCP(ΔβHD) tridomain from PcdK.

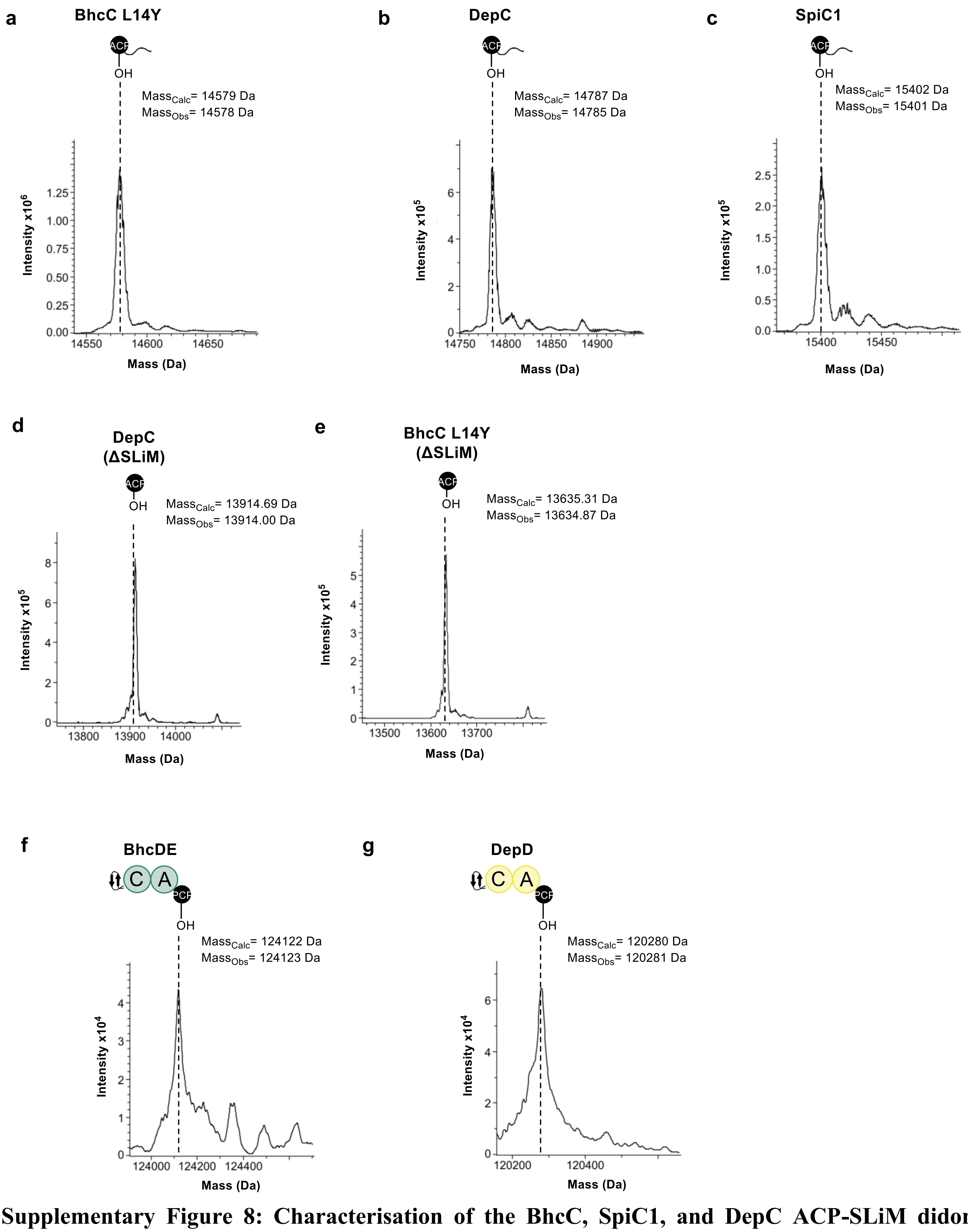

Supplementary Figure 8: Characterisation of the BhcC, SpiC1, and DepC ACP-SLiM didomains, DepC, BhcC *apo*-ACP (ΔSLiM) domains, and the βHD-C-A-PCP tetradomains excised from BhcDE and DepD. a, Intact protein mass spectra of the *apo* ACP-SLiM didomain from BhcC L14Y. The L14Y mutation was introduced into the His-tag to aid protein quantification **b**, Intact protein mass spectra of the *apo* ACP-SLiM didomain from DepC. **c**, Intact protein mass spectra of the *apo* ACP-SLiM didomain from SpiC1. **d**, Intact protein mass spectra of the *apo* ACP domain from DepC(ΔSLiM). **e**, Intact protein mass spectra of the *apo* ACP domain from BhcC L14Y(ΔSLiM). **f,** Intact protein mass spectra of the *apo* βHD-C-A-PCP tetradomain from BhcDE. **g,** Intact protein mass spectra of the *apo* βHD-C-A-PCP tetradomain from DepD.

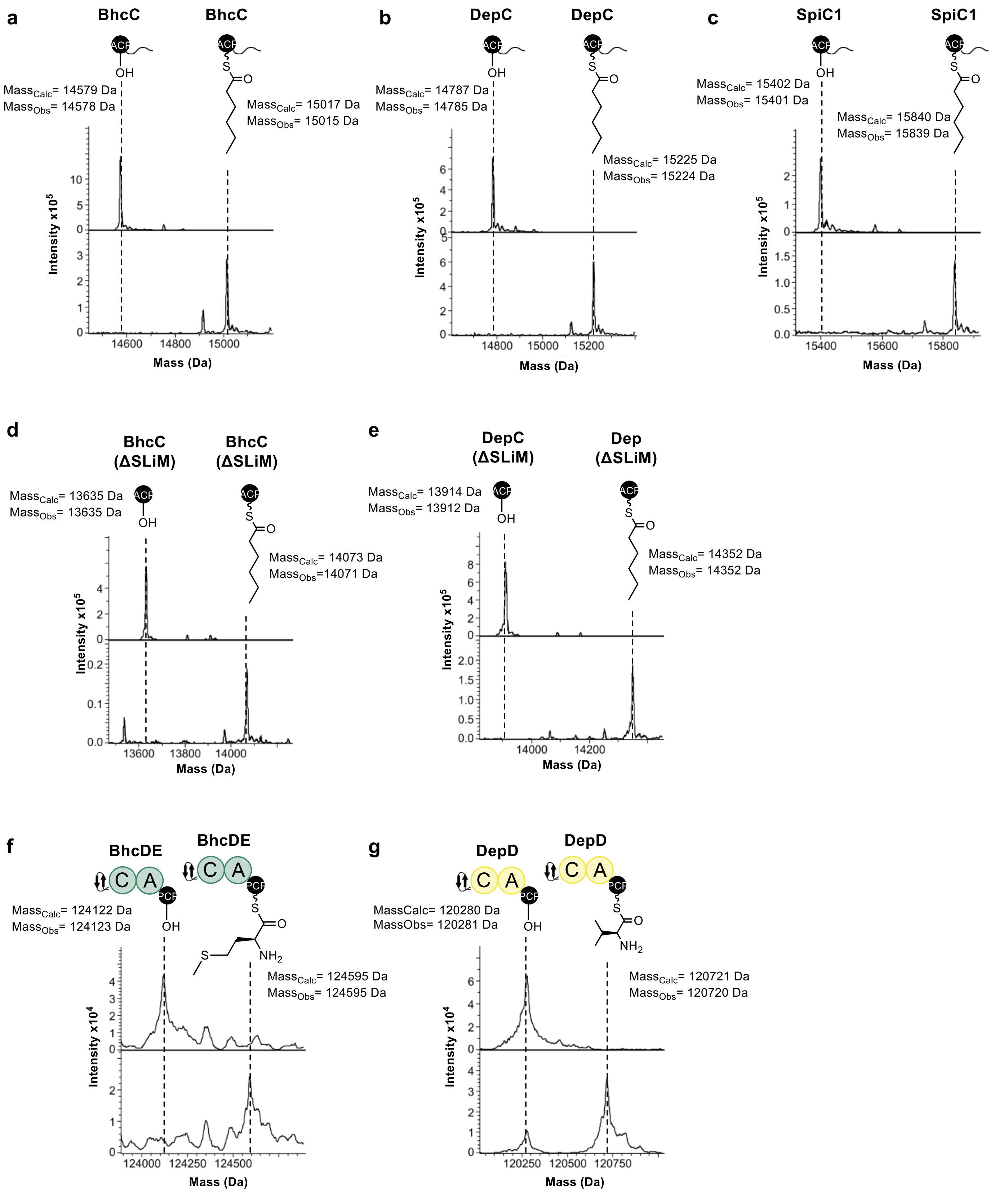

Supplementary Figure 9: Posttranslational phosphopantetheinylation and (amino)acylation of the DepC, BhcC, and SpiC1 *apo*-ACP-SLiM didomains, DepC, BhcC *apo*-ACP (ΔSLiM) domains, and DepD and BhcDE *apo*-βHD-C-A-PCP tetradomains. a, Intact protein mass spectra of the *apo* and hexanoyl-ACP-SLiM didomain from BhcC. **b**, Intact protein mass spectra of the *apo* and hexanoyl-ACP-SLiM didomain from DepC. **c**, Intact protein mass spectra of the *apo* and hexanoyl-ACP-SLiM didomain from SpiC1. **d**, Intact protein mass spectra of the *apo* and hexanoyl-ACP domain from BhcC(ΔSLiM). **e**, Intact protein mass spectra of the *apo* and hexanoyl-ACP domain from DepC(ΔSLiM). **f,** Intact protein mass spectra of the *apo* and L-methioninyl-βHD-C-A-PCP tetradomain from BhcDE. **g,** Intact protein mass spectra of the *apo* and L-valinyl-βHD-C-A-PCP tetradomain from DepD.

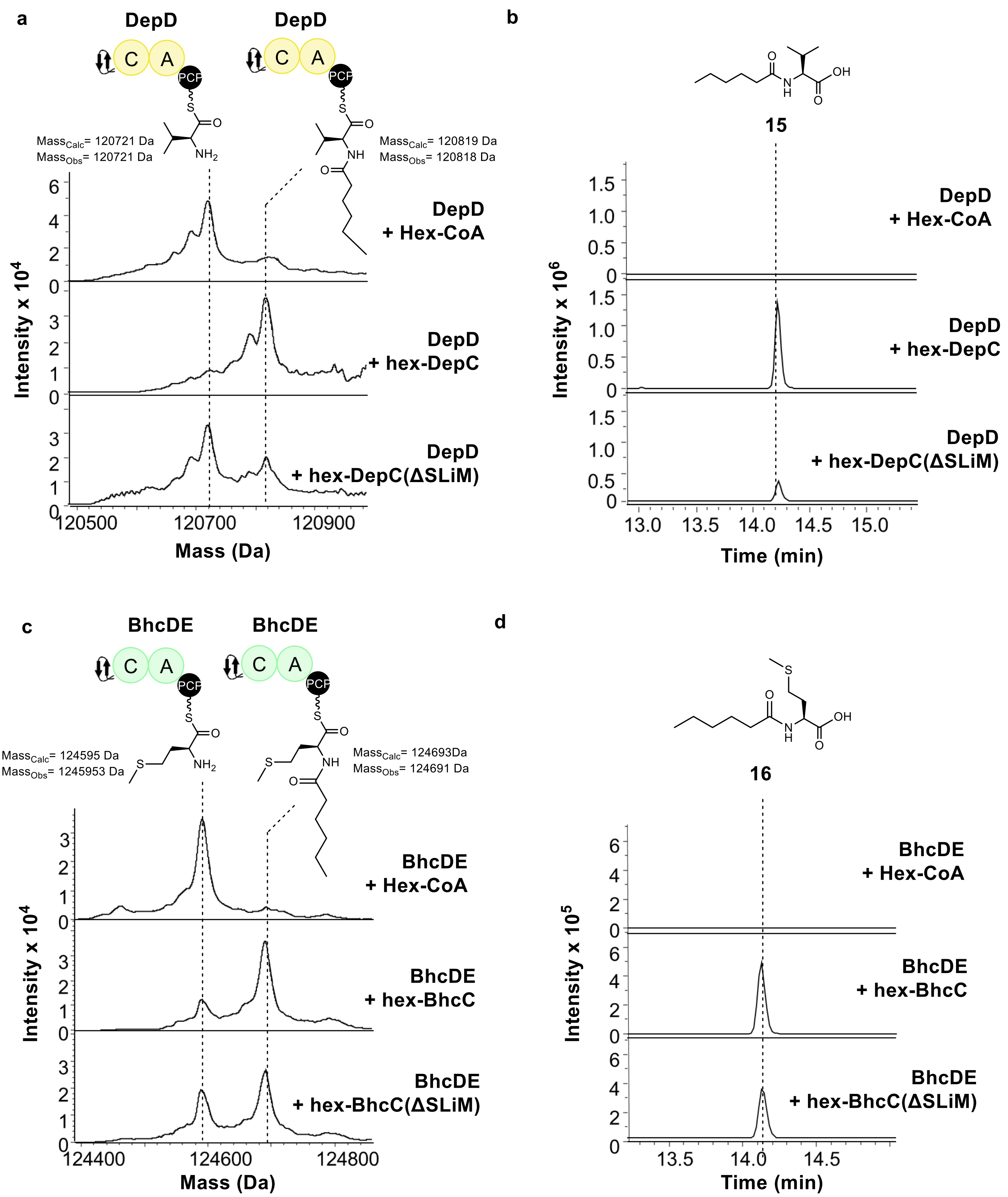

Supplementary Figure 10: Evaluation of SLiM importance for mediating the DepC-DepD and BhcC-BhcDE interface. a, Intact protein mass spectra of the DepD valinyl-βHD-C-A-PCP tetradomain after incubation with hexanoyl-CoA, the DepC hexanoyl-ACP-SLiM didomain and the DepC hexanoyl-ACP-ΔSLiM construct. **b**, Extracted ion chromatograms at m/z = 216.1590 ± 0.002 (corresponding to $[M+H]^+$ for **15**) from UHPLC-Q-ToF-MS analyses of hydrolytic release products resulting from incubation of the valinyl-βHD-C-A-PCP DepD tetradomain with hexanoyl-CoA, the DepC hexanoyl-ACP-SLiM didomain, or the DepC hexanoyl-ACP-ΔSLiM construct. **c**, Intact protein mass spectra of the methionyl-βHD-C-A-PCP BhcDE tetradomain after incubation with hexanoyl-CoA, the BhcC hexanoyl-ACP-SLiM didomain and the BhcC hexanoyl-ACP-ΔSLiM construct. **d**, Extracted ion chromatograms at m/z = 248.1315 ± 0.002 (corresponding to $[M+H]^+$ for **16**) from UHPLC-Q-ToF-MS analyses of hydrolytic release products resulting from incubation of the methionyl-βHD-C-A-PCP BhcDE tetradomain with hexanoyl-CoA, the BhcC hexanoyl-ACP-SLiM didomain, or the BhcC hexanoyl-ACP-ΔSLiM construct.

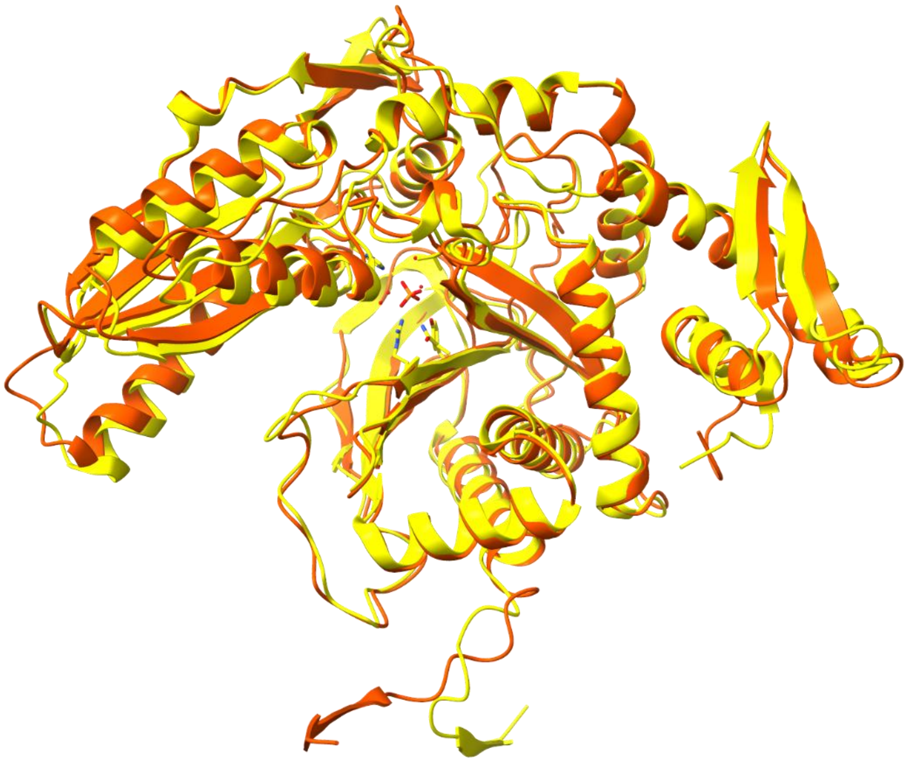

Supplementary Figure 11: Overlay of X-ray crystal structure and AlphaFold 3 model of Bamb_5915 βHD-C didomain. The crystal structure (PDB ID: 6CGO) is yellow and AlphaFold model is orange.

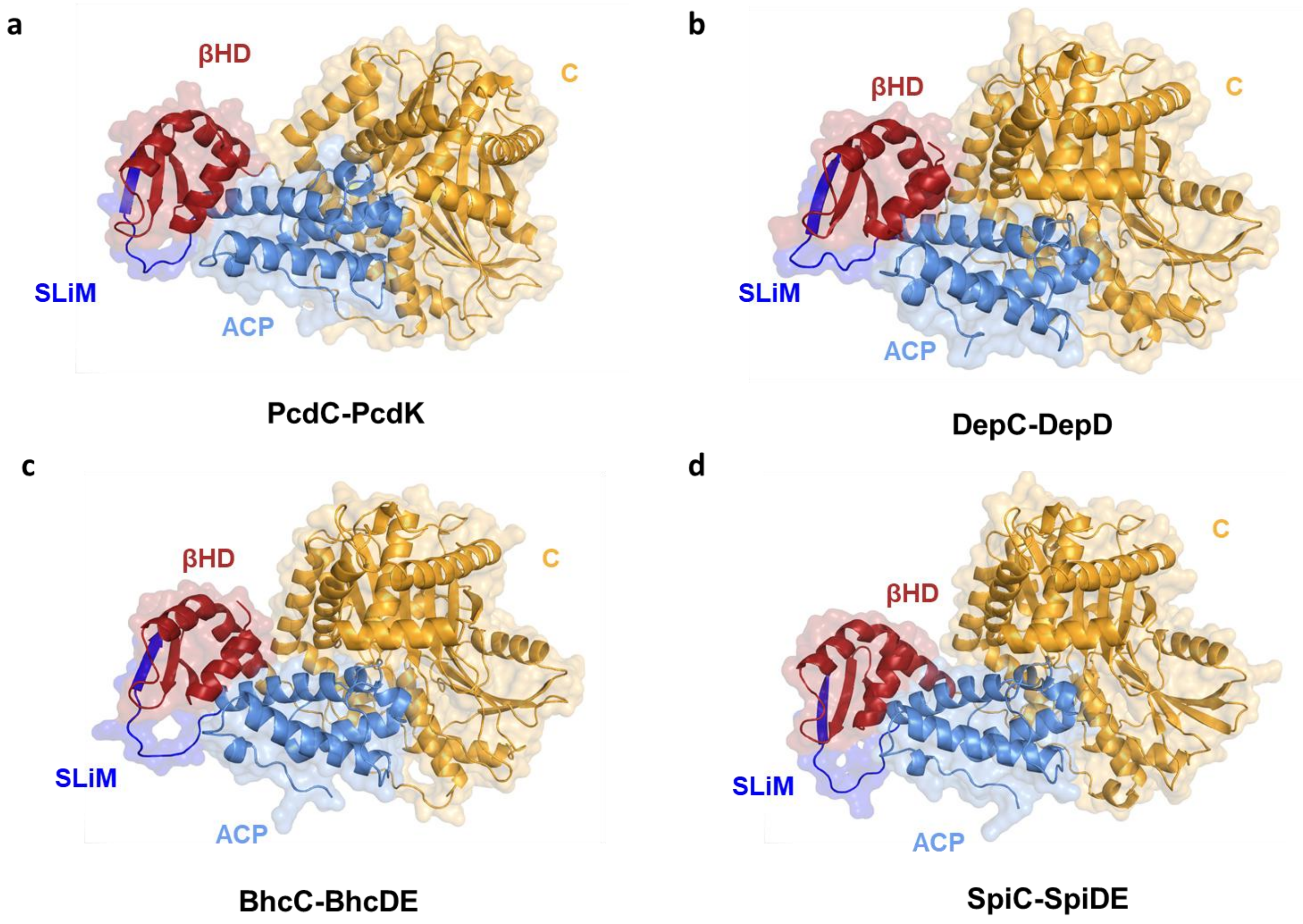

Supplementary Figure 12: Comparison of structural models for complexes of ACP-SLiM (light blue / dark blue) and βHD-C (red / yellow) didomains from depsipeptide HDAC inhibitor assembly lines showing a conserved interaction epitope between ACP and βHD domains. **a**, AlphaFold model of PcdC ACP-SLiM and PcdK βHD-C didomains from FR901375 assembly line. **b**, AlphaFold of DepD ACP-SLiM and DepD βHD-C didomains from romidepsin assembly line. **c**, AlphaFold of BhcC ACP-SLiM and BhcDE βHD-C didomains from burkholdacs assembly line. **d**, AlphaFold of SpiC ACP-SLiM and SpiDE βHD-C didomains from burkholdacs assembly line

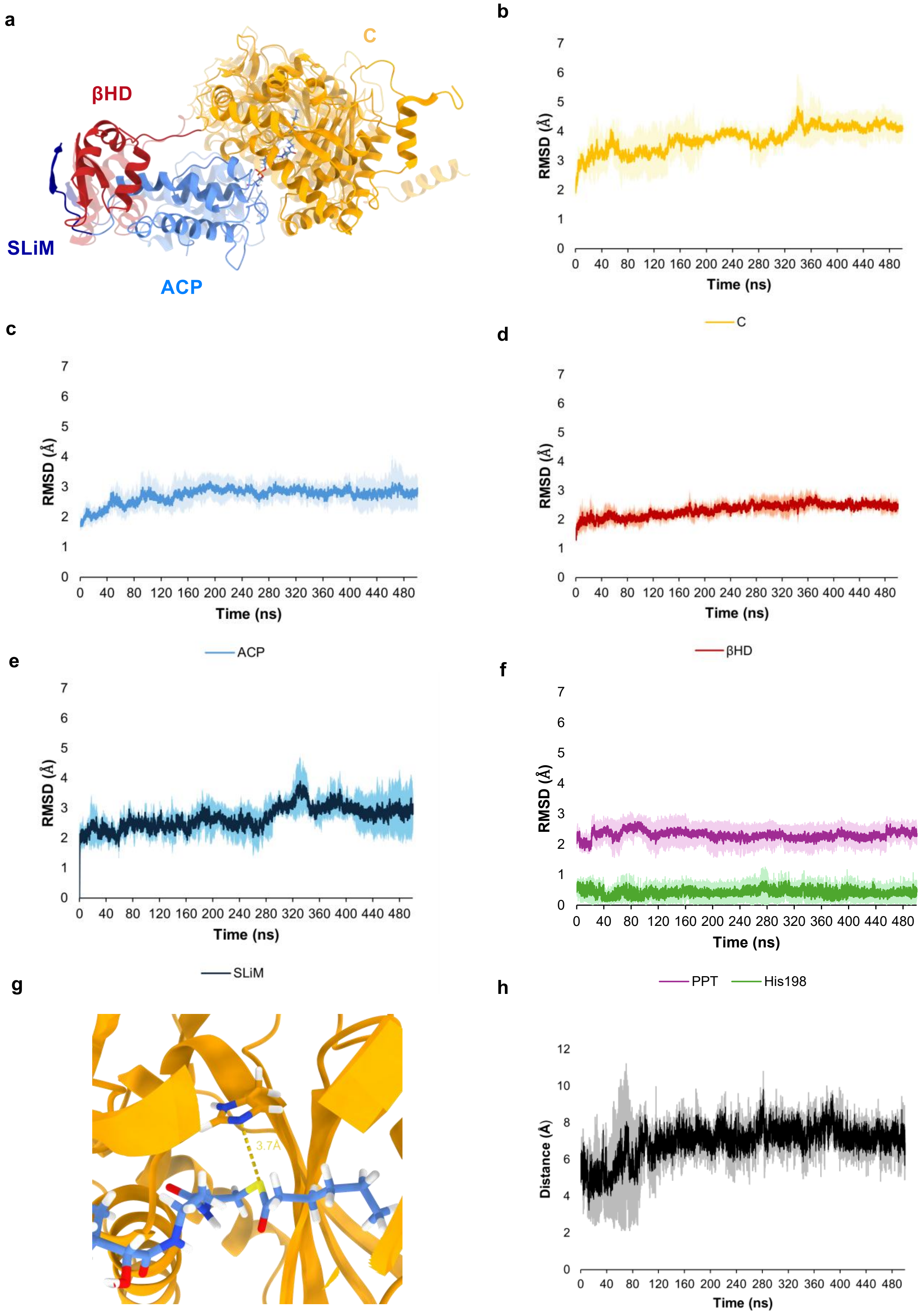

Supplementary Figure 13: Molecular dynamics simulations of the complex between the PcdC ACP-SLIM and PcdK βHD-C didomains. **a**, Overlay of a representative frame from the 500 ns MD simulation of the complex (opaque) against the initial complex model (transparent). The hexanoyl mimic of the pharmacophore was attached via a thioester linkage to the phosphopantetheine arm of the PcdC ACP-SLiM didomain (blue). Stable association with the PcdK βHD-C didomain (red-yellow respectively) was observed throughout the simulation. RMSD analysis of the C (**b**), ACP (**c**), βHD (**d**) and SLiM (**e**) domains, as well as the phosphopantetheinyl group and PcdK active site H198 residue (**f**), across the three independent 500 ns

MD simulations showed little variation throughout. RMSD was calculated relative to the starting model used for the simulation. **g**, View of the C domain active site in a randomly selected frame from the simulation, highlighting the proximity of the His198 residue, known to play an important catalytic role in other C domains, to the thioester linkage of the phosphopantetheine-bound hexanoyl group. **h**, Plot of the distance between the sulfur atom linking the phosphopanthetheine arm to the pharmacophore and the ε2 nitrogen atom in the His198 side chain of (S:N distance) versus frame during the course of the simulation. Initially the mean distance between the sulfur and nitrogen atoms sits at approximately 5Å. This increases to approximately 7-8 Å after 70 ns due to movement of the substrate within the pocket, where it stays for the remainder of the simulation. Due to the choice of the Bernedsen barostat for the NPT runs, fluctuations may be artificially suppressed and stability overestimated. Source data are provided in the Source Data file.

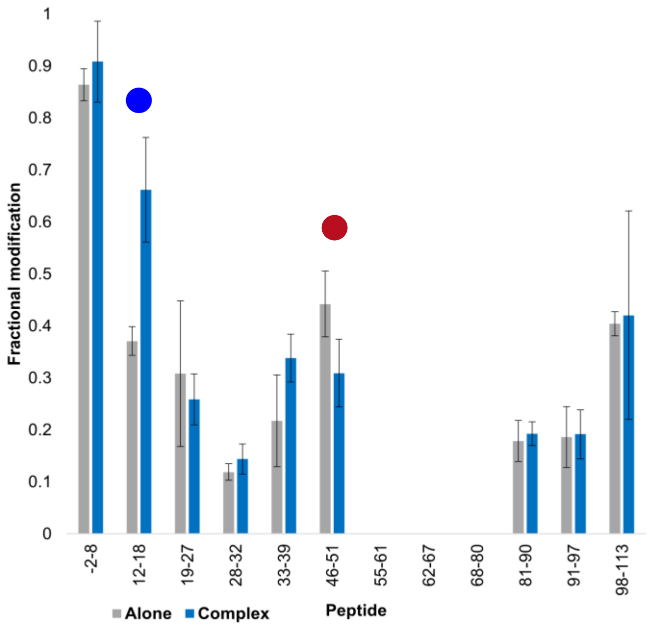

Supplementary Figure 14: ACP peptide modification results of carbene footprinting MS analysis of the interaction between the PcdC ACP-SLiM, PcdC ΔSLiM ACP, and PcdK βHD-C didomains. Fractional modification of His-cleaved PcdC ACP-SLiM didomain peptides (n=3). Masked and unmasked peptides are highlighted with red and blue circles, respectively. Paired bars not highlighted with circles are assigned as "no change". Peptides for which no labelled and/or unlabelled species were detected are designated "no coverage". Error bars are +/- two standard deviation of the mean. Each measurement was conducted in triplicate. Residue numbering starts at the Gly (-2) appended to the N-terminus of the native protein sequence following thrombin cleavage of the His-tag. Source data are provided in the Source Data file.

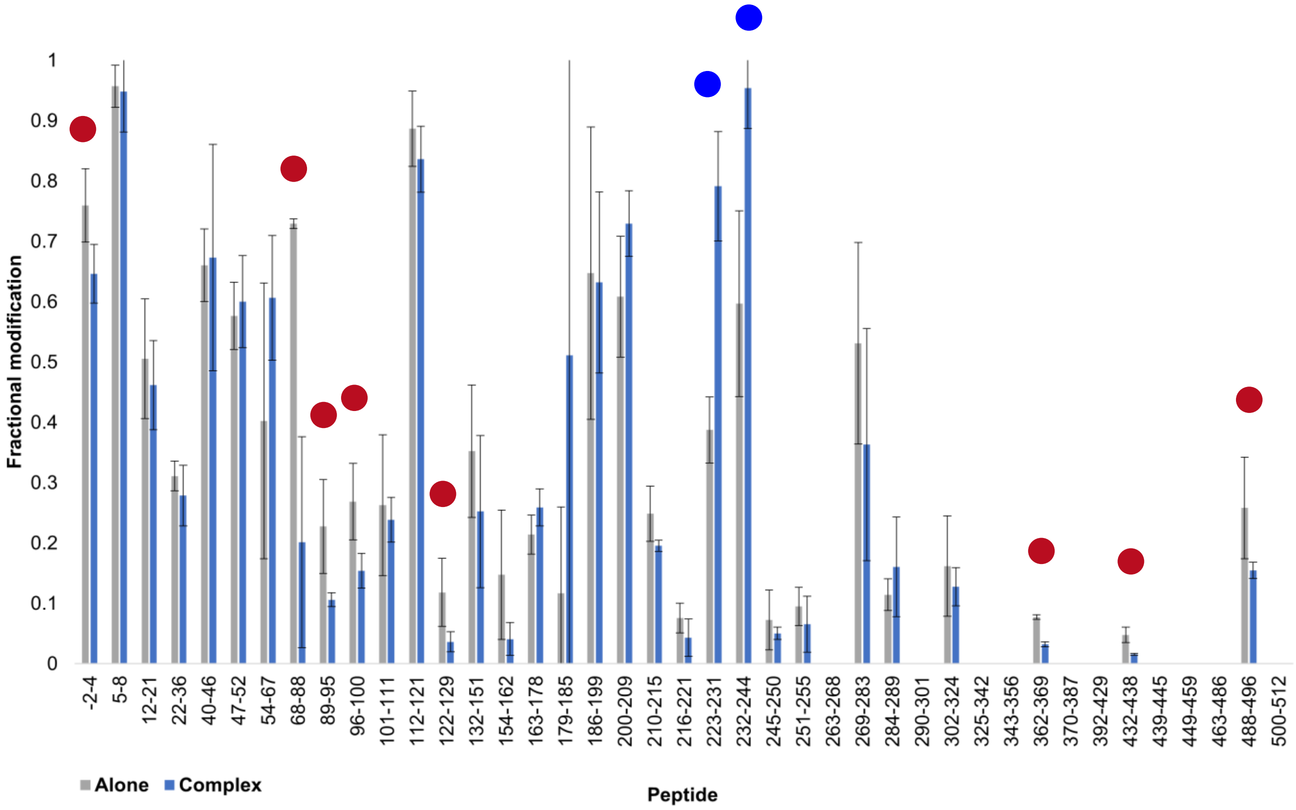

Supplementary Figure 15: PcdK βHD-C peptide modification results of carbene footprinting MS analysis of the interaction between the PcdC ACP-SLiM, and PcdK βHD-C didomains. Fractional modification of His-tag-cleaved PcdK βHD-C didomains upon incubation with PcdC ACP-SLiM didomain (n=3). Masked and unmasked peptides are highlighted with red and blue circles, respectively. Paired bars not highlighted with circles are assigned as "no change". Peptides for which no labelled and/or unlabelled species were detected are designated "no coverage". Error bars are +/- two standard deviation of the mean. Each measurement was conducted in triplicate. Residue numbering starts at the Gly (-2) appended to the N-terminus of the native protein sequence following thrombin cleavage of the His-tag. Source data are provided in the Source Data file.

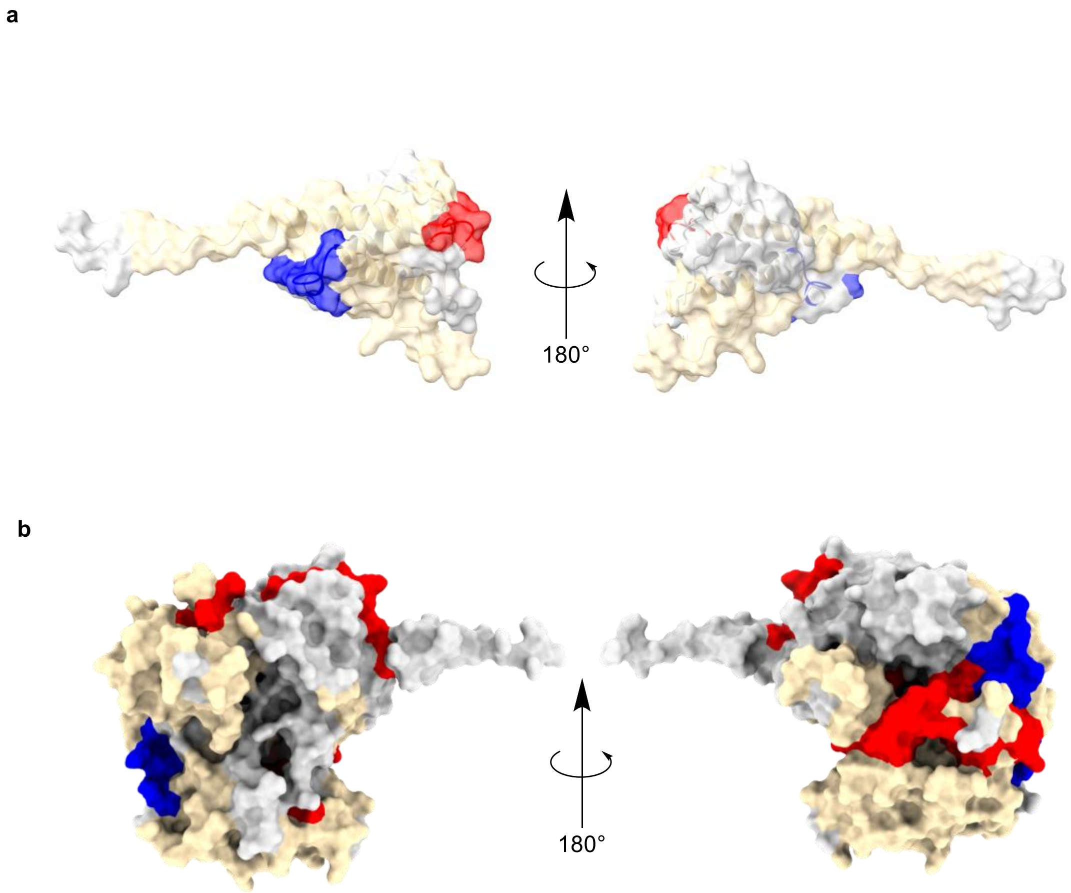

Supplementary Figure 16: Mapping of WT PcdC ACP-SLiM-PcdK βHD-C carbene footprinting results onto PcdC ACP-SLiM and PcdK βHD-C didomain AlphaFold models. a, AlphaFold 2.1 model of the PcdC ACP-SLiM didomain. **b,** AlphaFold 2.1 model of the PcdK βHD-C didomain. The locations of peptides that are masked (red), unmasked (blue), unaffected (beige) and undetected (grey) in the analysis are highlighted.

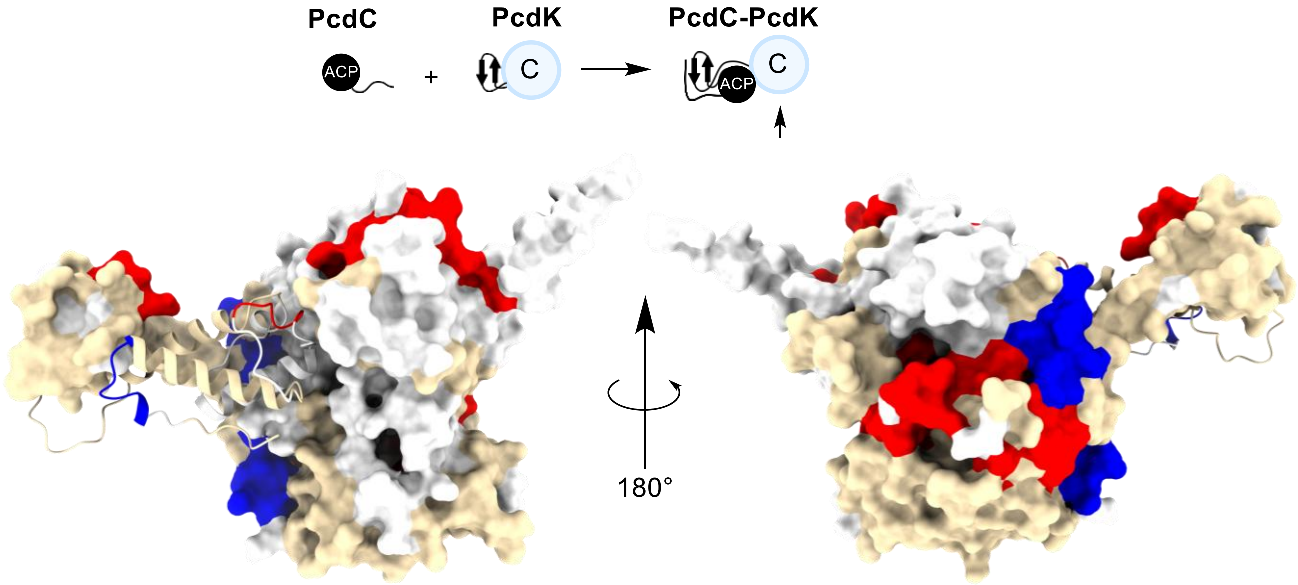

Supplementary Figure 17: Interpretation of carbene footprinting results with PcdC-PcdK AlphaFold multimer models. AlphaFold 2.1 model of PcdC ACP-SLiM-PcdK βHD-C didomain complex. The PcdC ACP-SLiM didomain is shown as cartoon, with transparent surface, and the PcdK βHD-C didomain is depicted as opaque surface representation. The masking of the ACP-SLiM region containing the peptide DMSLIE corresponding to residues 46-51, is consistent with binding of the ACP-SLiM didomain to the C domain in a conformation suitable for catalytic activity, as these residues occur on the same face of the ACP as the phosphopantetheinylated Ser. Unmasking of the peptide QLRGHSA corresponding to residues 12-18 may be a result of conformational changes resulting from SLiM binding, as when unbound the SLiM could sequester to the ACP itself. On the PcdK side, unmasking and complimentary masking in several regions of the C domain suggest it undergoes significant conformational change upon association with the ACP domain, consistent with observations in related systems.[25]

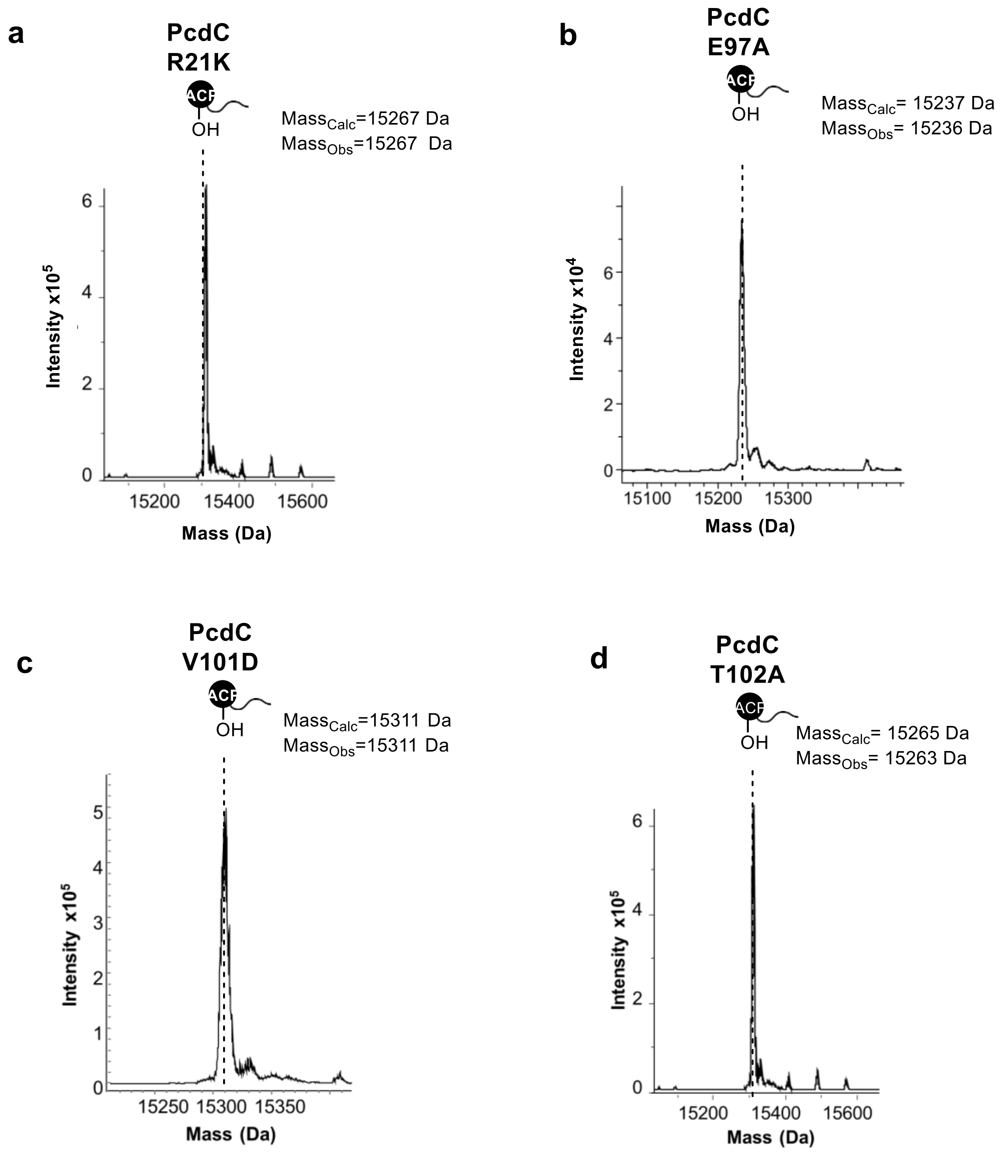

Supplementary Figure 18: Intact protein MS characterisation of PcdC ACP-SLiM mutants: a, R21K mutant. **b,** E97A mutant. **c,** V101D mutant. **d,** T102A mutant.

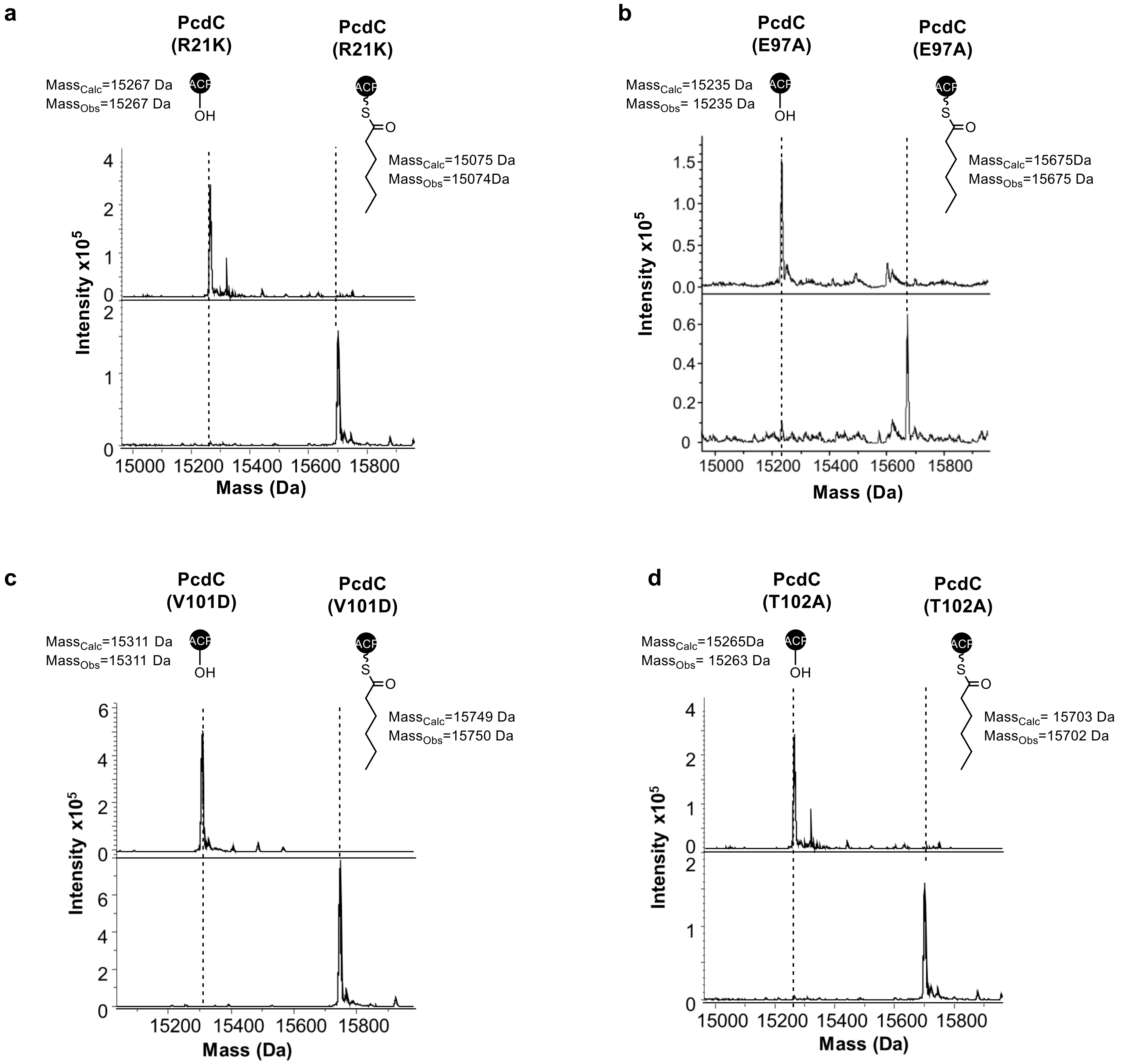

Supplementary Figure 19. Posttranslational phosphopantetheinylation and acylation of the PcdC ACP-SLiM mutants: a, R21K mutant. **b,** E97A mutant. **c,** V101D mutant. **d,** T102A mutant.

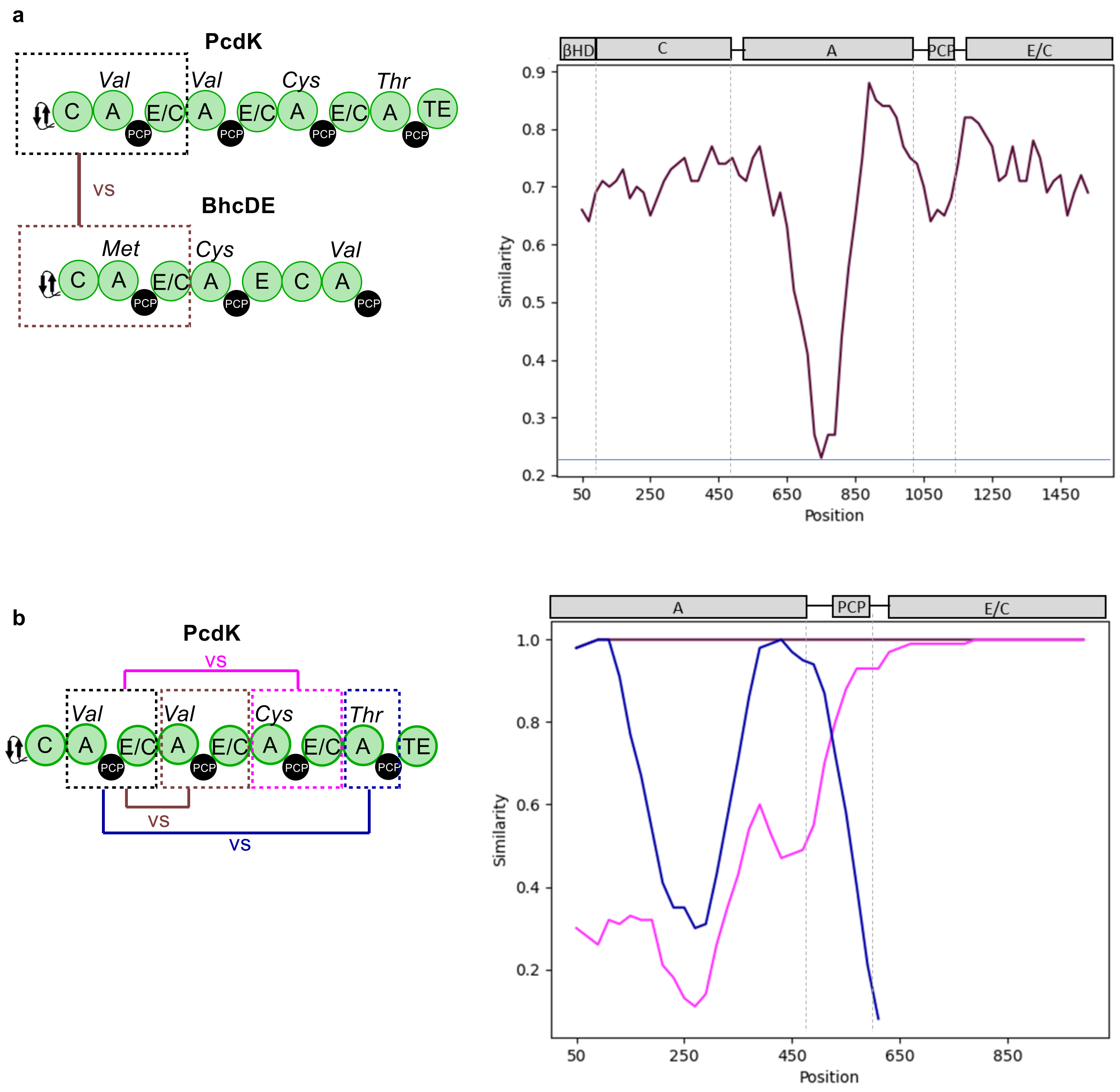

Supplementary Figure 20: Simplot analysis of sequence identities between N-terminal tetradomains of PcdK and BhcDE and the first, second and third set of PcdK A-PCP-E/C tridomains: a, Comparison of sequence identities between the N-terminal tetradomains of PcdK and BhcDE. **b**, Comparison of sequence identities between the first, second and third set of PcdK A-PCP-E/C tridomains. Regions compared are shown on the left with sequence identity plots shown on the right in the corresponding colour. Domain boundaries are indicated on top of the plot. Simplot settings: Distance model: identity; window length: 100; step: 20; Strip gap: 20; Plot refresh rate: every window.

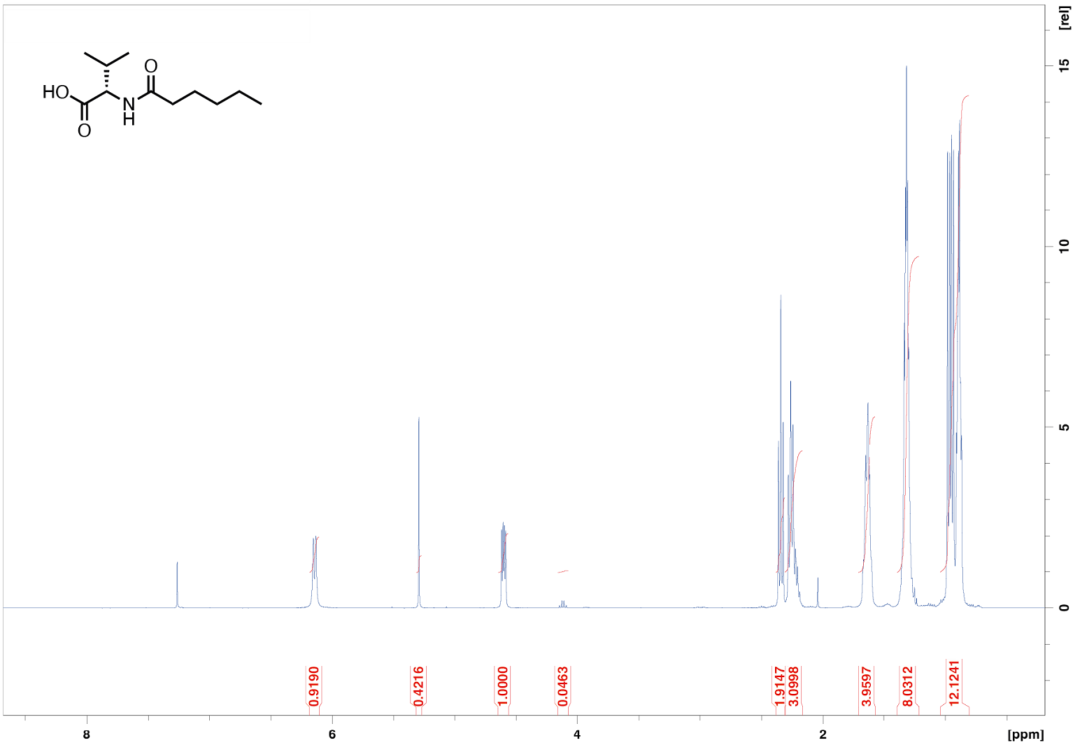

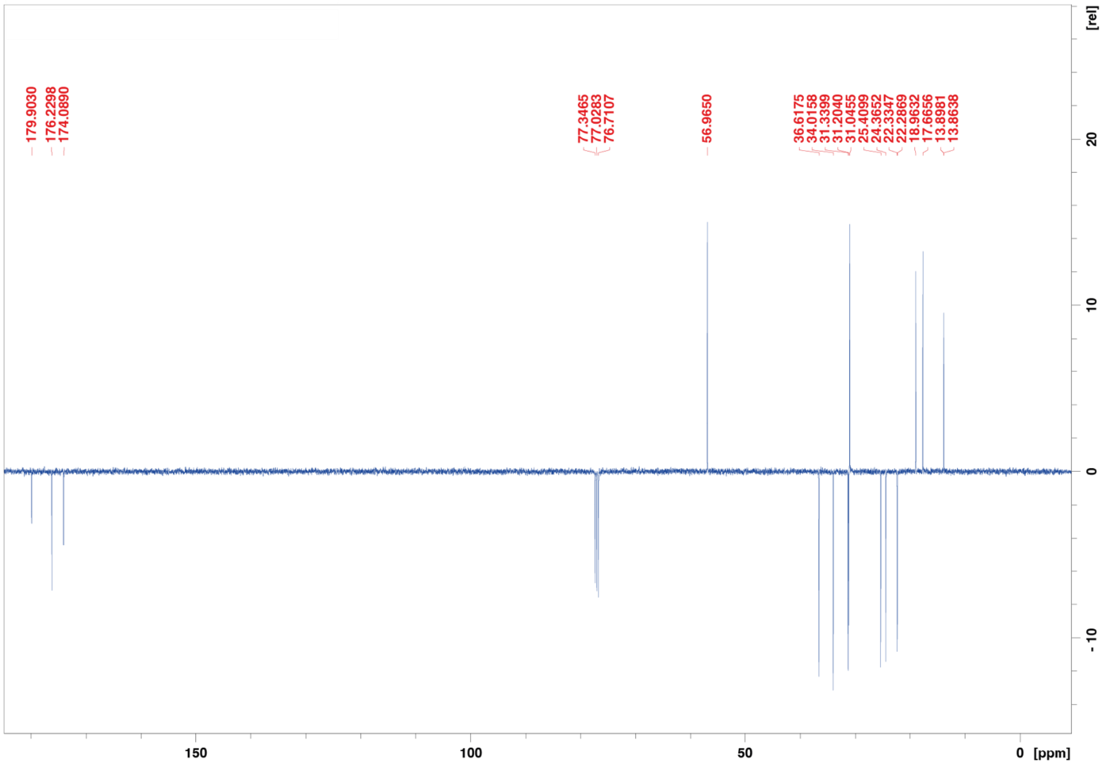

Supplementary Figure 21: ^{1}H and ^{13}C NMR spectra of *N*-hexanoyl-L-valine **15** recorded in $CDCl_3$

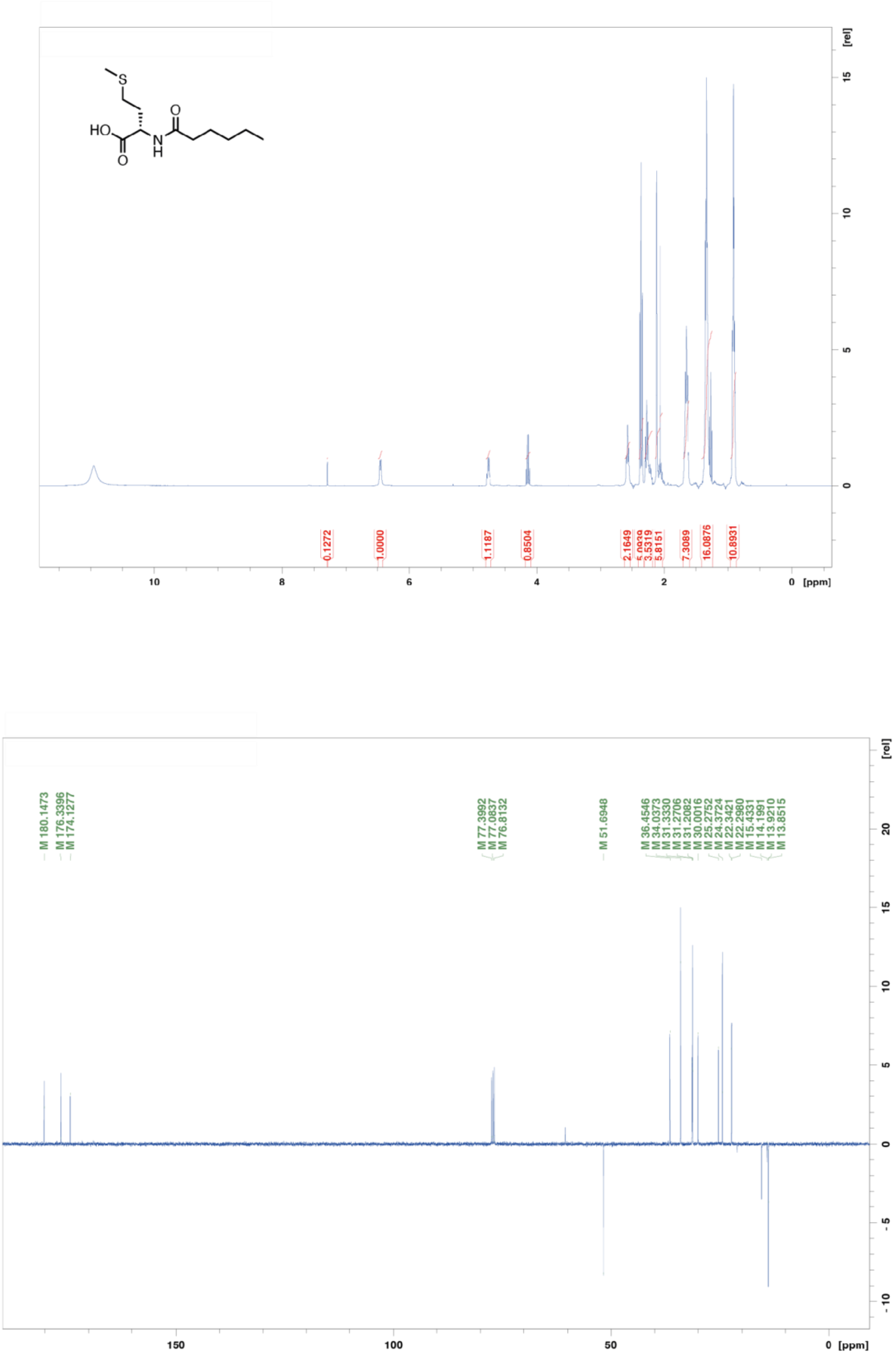

Supplementary Figure 22: ^{1}H and ^{13}C NMR spectra of *N*-hexanoyl-L-methionine **16** recorded in $CDCl_3$.

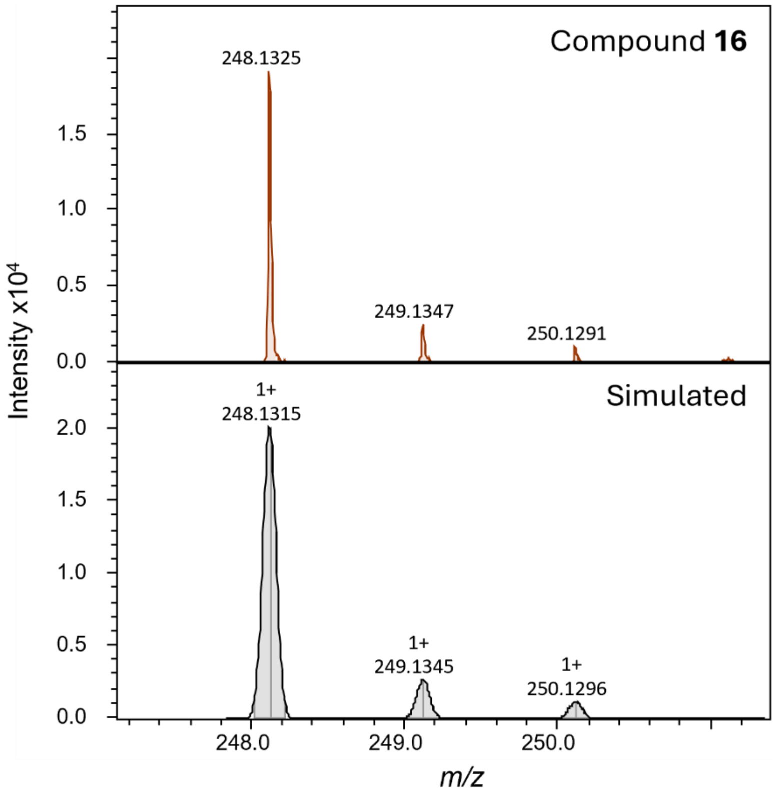

Supplementary Figure 23: High Resolution mass spectrum of the [M+H]$^+$ ion of *N*-hexanoyl-L-methionine 16 compared to simulated spectrum.

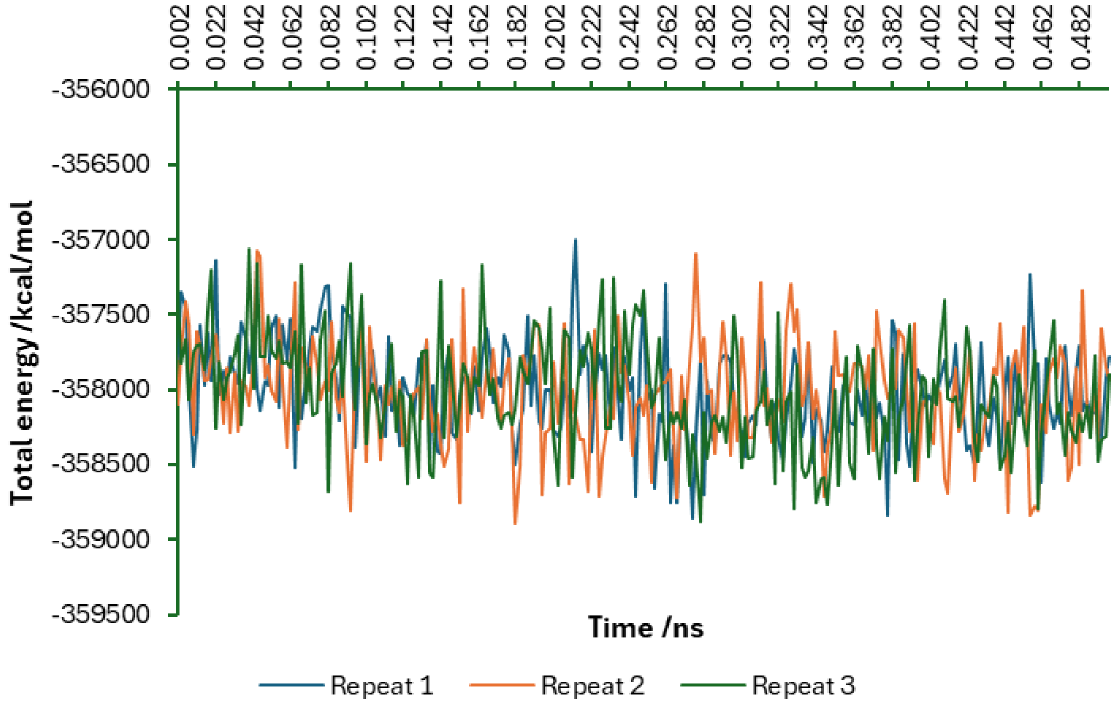

Supplementary Figure 24: Total energy of PcdC:PcdK molecular dynamics simulations during equilibration phase (n=3). Source data are provided in the Source Data file.

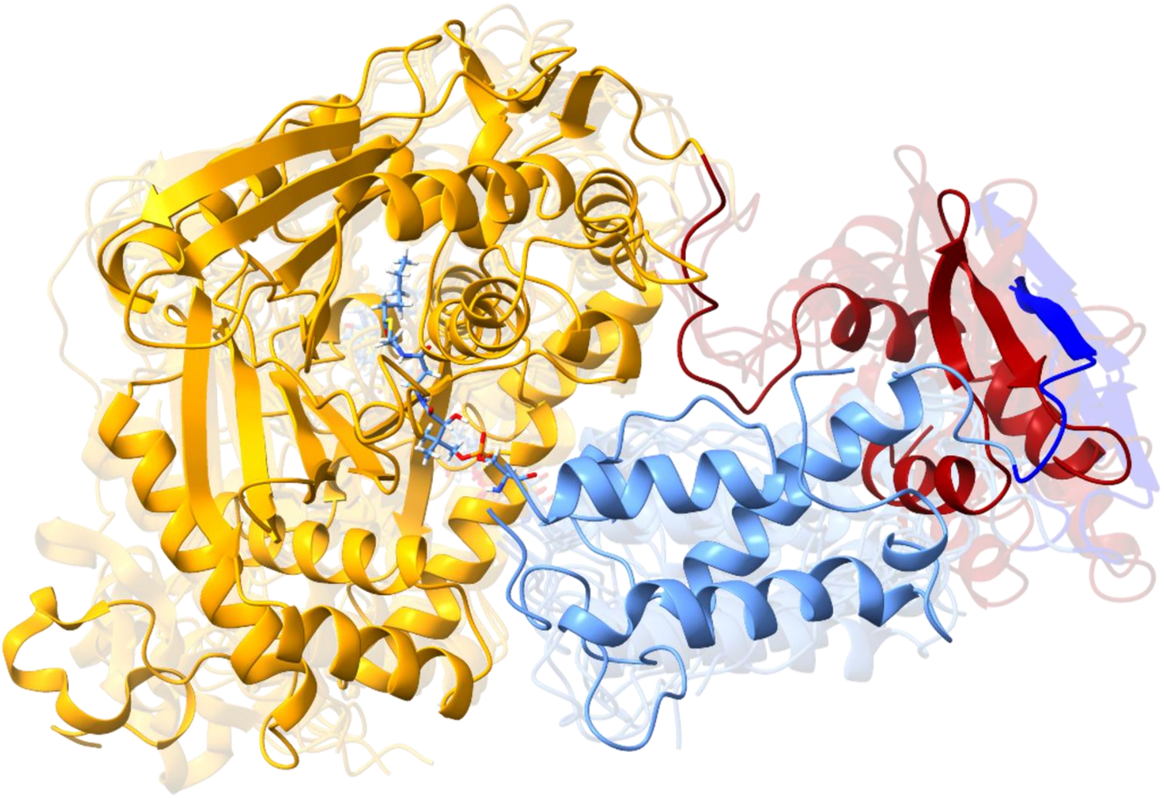

Supplementary Figure 25: Overlay of PcdC:PcdK accelerated MD simulation frames after 100, 200, 300, 400 and 500 ns (transparent). Final structure after 500 ns of simulation time is shown as opaque.

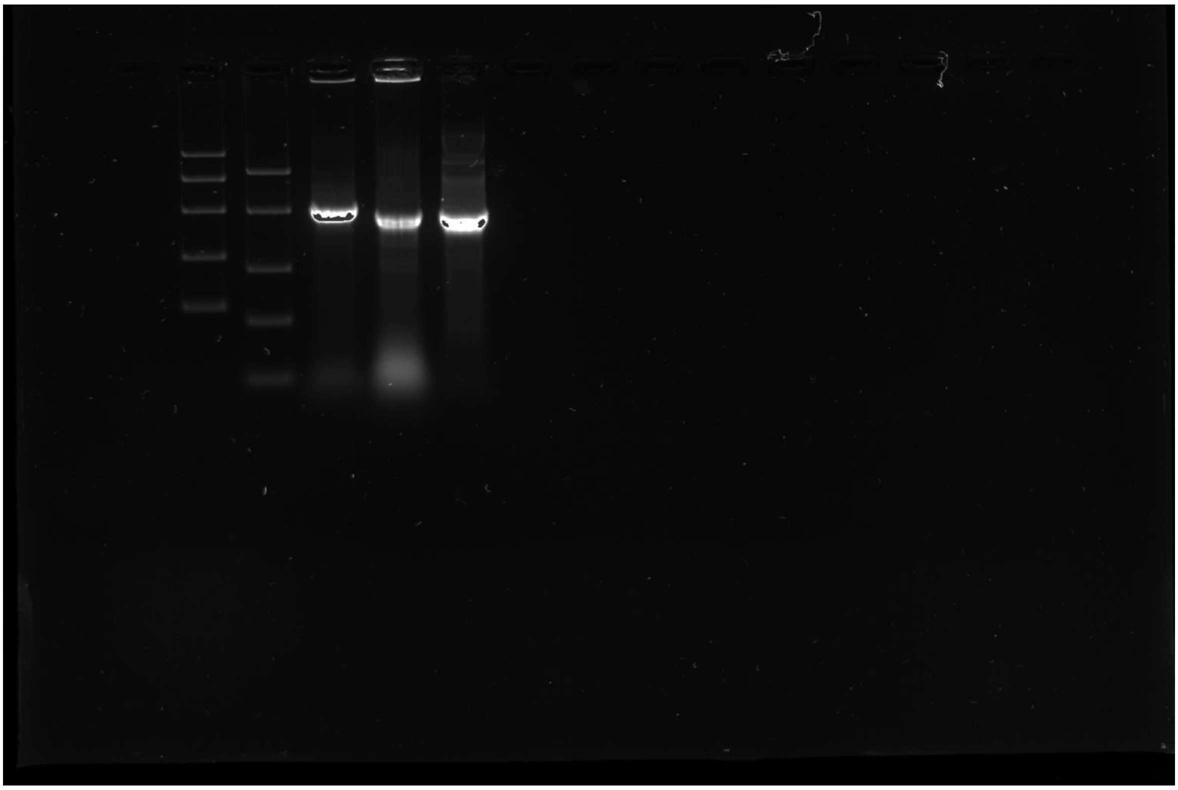

Uncropped gel of PcdK βHD deletion shown in Supplementary Figure 3.

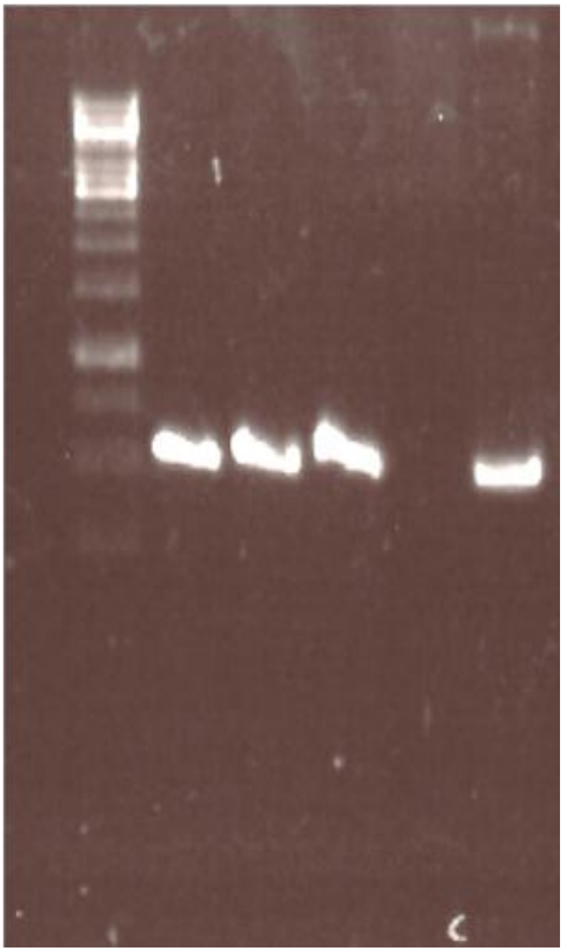

Uncropped gel of PcdK deletion shown in Supplementary Figure 3.

Protein sequences (N-terminal affinity purification tag highlighted in yellow):
PcdC ACP-SLiM didomain
MKHHHHHHHHSSGLVPRGSHMPMRRPVFDFEQLRGHSADARRALIAEYLGGELQFILSSDVELSH
DMSLIELGMDSLTGSELRNAIERSTGVYVPMQHFIDGSPLNTVVEVIVGQLERRLVTTQSVSPGTST
EKITL

PcdK βHD-C-A-PCP tetradomain
MKHHHHHHHHSSGLVPRGSHMNIERLMTDLVDAGATLCRNGDMLQVKAPPGALNSELVERLRQA
KETLLQMLDDNTPHAVPLPSPGEGGNTCALSPGQASLVLATRLGDPAMYNEQAAIELAGPVNAQVI
EGAFAMLARKHDILRTVFVDGDPMQQTVLPTPVVQFAVTAVDNDNSLRALAADIAKLPFAPQQPL
WRVDLFSTFERPAVLVLTIHHAIFDRWSMGVLIRDLNTYLDAPVPEEAPLPHLNYRDFAAWQRRW
MNTSDYTSQLDSWVEMLADIDEVPTIRSDYTRPAVRSRHGGTERIAIPADCITAASTFARERNTTLFT
TLFSVFALLLQRYTGEARTLTLTPAANRPFQAAEDIAGYFVNLVPLVADVRDNDNFTSFVERMRGV
TARSFAHQGVPIESIAERLRSRGGPPLSQLAQTVFAFQNVQLPTVHIAGGRAKPFDLDSPFARFDLYL
SIENDERGTFAVWQYSTDLFDASTICRLGVHYVALLSAALASPETDVRMLPVLSDTEQAQLLYGFN
ATQSDFPQDALIHQLFEAQAQRSPTATALVFGQQTLSYGELNRRANRLAHHLIALGVRPDDRVALC
VERSPEMVVGLLAILKAGGAYVPLDPDYPAERRAYMLADAAPVALLTQRSLIDESGPTLPTVLLDV
QNPAIEELADSNPDAGAMGLTARHLAYVIYTSGSTGQPKGVMVEHLNVNRLVINNTYADIGPNDC
VAHCANIAFDASTWEIWSALLNGGRLYLISQSVLLDPQRFRDALIHGQVTALWLTAGLFNQYVDGL
IPVFGQLRYLLVGGDVLDARKIGQLLAAESQPEHLLNGYGPTETTTFAATHAITAPLDVTRSIPIGRPI
ANTRIYILDSHGQPAPLGVAGELHIAGAGVARGYLNRPELTAERFINDPFCADPHARMYKTGDLGR
WLPDGTIEYLGRNDFQVKLRGFRIELGEIEAALARCEGVRDAVVIPREDVPGDKRLVAYVLPQTGV
EIAPAELRQQLARQLAEYMLPGAFVTLDTFPLTPNGKLDRQALPVPDLTALATRGYQAPVGEMETT

LAQIWQDLLGLARVGRYDNFFELGGHSLLGVSLIERLRELGLTLAVRTVFASPALADMAQAISAHQ
DHASAFVVPDNLIP

PcdCΔSLiM ACP domain
MKHHHHHHHHHSSGLVPRGSHMPMRRPVFDFEQLRGHSADARRALIAEYLGGELQFILSSDVELSH
DMSLIELGMDSLTGSELRNAIERSTGVYVPMQHFIDGSPLNTVVEVIVGQLERRLVTTQSVS

PcdKΔβHD C-A-PCP tridomain
MKHHHHHHHHHSSGLVPRGSHMHAVPLPSPGEGGNTCALSPGQASLVLATRLGDPAMYNEQAAIEL
AGPVNAQVIEGAFAMLARKHDILRTVFVDGDPMQQTVLPTPVVQFAVTAVDNDNSLRALAADIAK
LPFAPQQPLWRVDLFSTFERPAVLVLTIHHAIFDRWSMGVLIRDLNTYLDAPVPEEAPLPHLNYRDF
AAWQRRWMNTSDYTSQLDSWVEMLADIDEVPTIRSDYTRPAVRSRHGGTERIAIPADCITAASTFA
RERNTTLFTTLFSVFALLLQRYTGEARTLTLTPAANRPFQAAEDIAGYFVNLVPLVADVRDNDNFTS
FVERMRGVTARSFAHQGVPIESIAERLRSRGGPPLSQLAQTVFAFQNVQLPTVHIAGGRAKPFDLDS
PFARFDLYLSIENDERGTFAVWQYSTDLFDASTICRLGVHYVALLSAALASPETDVRMLPVLSDTEQ
AQLLYGFNATQSDFPQDALIHQLFEAQAQRSPTATALVFGQQTLSYGELNRRANRLAHHLIALGVR
PDDRVALCVERSPEMVVGLLAILKAGGAYVPLDPDYPAERRAYMLADAAPVALLTQRSLIDESGPT
LPTVLLDVQNPAIEELADSNPDAGAMGLTARHLAYVIYTSGSTGQPKGVMVEHLNVNRLVINNTY
ADIGPNDCVAHCANIAFDASTWEIWSALLNGGRLYLISQSVLLDPQRFRDALIHGQVTALWLTAGL
FNQYVDGLIPVFGQLRYLLVGGDVLDARKIGQLLAAESQPEHLLNGYGPTETTTFAATHAITAPLD
VTRSIPIGRPIANTRIYILDSHGQPAPLGVAGELHIAGAGVARGYLNRPELTAERFINDPFCADPHAR
MYKTGDLGRWLPDGTIEYLGRNDFQVKLRGFRIELGEIEAALARCEGVRDAVVIPREDVPGDKRLV
AYVLPQTGVEIAPAELRQQLARQLAEYMLPGAFVTLDTFPLTPNGKLDRQALPVPDLTALATRGYQ
APVGEMETTLAQIWQDLLGLARVGRYDNFFELGGHSLLGVSLIERLRELGLTLAVRTVFASPALAD
MAQAISAHQDHASAFVVPDNLIP

DepC ACP-SLiM didomain
MKHHHHHHHHHSSGLVPRGSHMSARTLSFDQLRGRPAGERRARVADYLEAELRAVLSSPAALPRQS
SLLDLGVDSLTGAELRNELERALGVSVAITHLIDGSSLDELIDQVMAQLERKLVTEQHSAVGADTEE
ITL

DepCΔSLiM ACP domain
MKHHHHHHHHHSSGLVPRGSHMSARTLSFDQLRGRPAGERRARVADYLEAELRAVLSSPAALPRQS
SLLDLGVDSLTGAELRNELERALGVSVAITHLIDGSSLDELIDQVMAQLERKLVTEQHSAVG

BhcC ACP-SLiM didomain
MKHHHHHHHHHSSGYVPRGSHMRARAFDIEQLRGRPAAARRALIAGHLEAELRAVLSSASALSHQA
SLIELGVDSLTGSELRNAIERSMGVSVSISNLIDGSSLDAVIETVAAQIERRLVTEHSSTTGAETEEITL

BhcCΔSLiM ACP domain
MKHHHHHHHHHSSGYVPRGSHMRARAFDIEQLRGRPAAARRALIAGHLEAELRAVLSSASALSHQA
SLIELGVDSLTGSELRNAIERSMGVSVSISNLIDGSSLDAVIETVAAQIERRLVTEHSSTT

SpiC1 ACP-SLiM didomain
MKHHHHHHHHHSSGLVPRGSHMMRRPVFDFEQLRGHNADARRALITEYLGRELQFILSSDVELPHD
VSLIELGMDSLTGSELRNAIERSTGIYVPMQHFIDGSPLNTAVEVIVGQLERRLVTTLPGRQGNSTEK
MTL

DepD βHD-C-A-PCP tetradomain

MKHHHHHHHHHSSGLVPRGSHMMTMARLMTDLADAGVTLRRRGDQLQVQAPQGALDAALVARL
REAKEELLRVLDDEGARAAPLAPAQPGEAGDAAALSPGQARLVAATRLGDPAMYNEQAAIELAD
AVDAEAVARAFAALARRHDILRTVFSDGEPVRQTVLPEPIVTLQAWTVDGDDALRARAADLARLP
FAAGAPMWRVDLFSTPERAAVLVLTIHHAIFDRWSMSVLIRDFSAYLALPDAAEAPASGLSYRDYS
AWQRRWMASPDYAAQLDAWVDDLAEVDEVPAIRGDRPRPPAMSGRGGTERFEIPADCMDAAAA
FSRSRNTTLFTTLFSAFALLQHRYTGEARALTLTPAANRPFQAAEEIAGYFVNLVALATEVGEGDSF
GALVDRARDASARAFARQGVPLDAIVERLRARGGPRHEQFAQTVFAFQNVRLPAVRTASGAAVPF
DLDSPFARFDLYLSIEGDERGTFAVWQYNTDLYEAATIRQLGEHYLALLRAALASPDADARALPILS
AEEEARLRGWGRHELPYRADAAIDRLFRERAADHPGRVALEQGGVRWTYAELDQWSDRAAGAL
RAAGVEAGAVVGVAGERSPRLLAAFLAVLKAGAAYLPLDPTYPAARLRAMTADAAPALMIIADG
LDAGWLGDYAGPVLSLADCEAGVARPLQSEARPAEAESLAYVMYTSGSTGQPKGVAVPHRAVAR
LATGGGYARLDASTVMLQQSPLGFDASTFEIWGCWLNGGRLVVAEPGMPFLDAASRDGVTTMWL
TADLFRMAVEEEPAALGGLRELLTGGDALPVASCRAFLEACPGVALINGYGPTENTTFTCSHRVTA
GDARRGSIPIGRPIGNTEVRVVDAGGRLVPVGVPGELWAGGDGLALGYLGRADLTAERFVAAPPP
DGGRWYRTGDRVRWRRDGVLEFLGRIDEQIKLRGYRIELGEIEATLGHYPGLSGCAVALRRSAADE
KQLVGYLVARPDSGEAADSAAVQAWLEARLPGYMVPRVWVWLDALPQSANGKVDRKRLPDPVV
ETGAAAAETEAEAALVEIWQGLLGLERVGVRDNFFALGGDSILSIQMASRAAERGLRLSPQQVFRY
PTIAELAAEGCAAEEAGAQAEQ

BhcDE βHD-C-A-PCP tetradomain

MKHHHHHHHHHSSGLVPRGSHMNIVRLMADLADAGITLRRRGDGLHVEGPPGALDAALVSRLRDA
KDGLLAMLDGDASRAAALPPPLPGEGGDAGALSPGQARLVAATRLGDPAMYNEQMAIELADAVD
AQAIGRAFVALARKHDILRTVFVGGEPMRQTVLPEPAVQIECTSVDGDGALRARAAEIARLPFAAG
RPLWRIDLFSTHERPLVLVLTIHHAIFDRWSMSVLIRDFSAYLASPDESDAPGGRLSYRDFAAWQRR
WMETPDYVAQLDAWVAALADLDEVPAIRGDRSRPPVPSWRGGTERFEIPADCIEAAAAFSRTRNT
TLFTTLFSVFALLQHRYTGDPRVVTLTPAANRPFQAAEDIAGYFVNLIALATSVRDDDSFGSLVERM
RDTTARAFAHQGVPLDAIVERLRARGGPQHDQFAQTAFAFQNVRLPAVRTASGTATPFDLDSPFAR
FDLYLSIEGDERGTFAVWQYNADLFDAQTVCRLGEHYVALLRAALAAPEANAHALPMLSDTERA
QLLADFNATRADFSHDAPIHQLFEAQAQRTPDATAAVFEERALSYAELNRRANRLAHHLIARGVRP
DDRVAICTGRGLDAVVGLLAVLKAGGAYVPLDPAYPAARLAYMLDDAAPAAVLTTAALADELAF
KLPTILLDAQNPSFESQPNDNPDPAALGLTSRHLAYVIYTSGSTGQPKGVMVEHGNLVNLIQSNRQH
FSGVGQARTCCWTSFGFDVCVFEIFMSLAMGGTVHVVPDRLRISADGFFQWLIAQRIEVAYLPPFL
VRRLREYSDELVASLSLRRILVGVEPLREKDLYRLERLLPELIVVNGYGPTETTVFSTSYLDMRDYD
RAAPIGRPIANTRIYILDSHGQPVPIGVAGEIHIAGAGVARGYLNRPELTAERFVSDSFAAAPNARMY
RTGDLGRWLPDGTIEYLGRNDFQVKIRGLRIELGEIEARLARCDGVRDAVVIAREDTPGDKRLVAY
VLPQSGVALVPAELRRQLAGQLAEHMLPSAFVMLDALPLTPNGKLDRKALPAPDQTAVVSRGYEA
PTGEVETALARIWQDLLGLEQVGRHDHFFELGGHSMLVVSMIERLRDLGWSLDVRSMFVAPVLAD
LAQAIDTRRGDAPAFAVPP

PcdK βHD-C didomain

MKHHHHHHHHHSSGLVPRGSHMNIERLMTDLVDAGATLCRNGDMLQVKAPPGALNSELVERLRQA
KETLLQMLDDNTPHAVPLPSPGEGGNTCALSPGQASLVLATRLGDPAMYNEQAAIELAGPVNAQVI
EGAFAMLARKHDILRTVFVDGDPMQQTVLPTPVVQFAVTAVDNDNSLRALAADIAKLPFAPQQPL
WRVDLFSTFERPAVLVLTIHHAIFDRWSMGVLIRDLNTYLDAPVPEEAPLPHLNYRDFAAWQRRW
MNTSDYTSQLDSWVEMLADIDEVPTIRSDYTRPAVRSRHGGTERIAIPADCITAASTFARERNTTLFT
TLFSVFALLLQRYTGEARTLTLTPAANRPFQAAEDIAGYFVNLVPLVADVRDNDNFTSFVERMRGV
TARSFAHQGVPIESIAERLRSRGGPPLSQLAQTVFAFQNVQLPTVHIAGGRAKPFDLDSPFARFDLYL

SIENDERGTFAVWQYSTDLFDASTICRLGVHYVALLSAALASPETDVRMLPVLSDTEQAQLLYGFN
ATQSDFP
