# Supplementary material for: Molecular basis for depsipeptide HDAC inhibitor combinatorial biosynthesis
Source: Nat Commun. 2026 Jul 1;17:5508. doi: 10.1038/s41467-026-74383-4 (PMC13324335; doi:10.1038/s41467-026-74383-4)
Supplement: Supplementary file 3 — Transparent Peer Review file [file 41467_2026_74383_MOESM3_ESM.pdf]

nature portfolio

Peer Review File

Molecular basis for depsipeptide HDAC inhibitor combinatorial biosynthesis

Corresponding Author: Professor Gregory Challis

This file contains all reviewer reports in order by version, followed by all author rebuttals in order by version.

Version 0:

Reviewer comments:

Reviewer #1

(Remarks to the Author)
This report by Passmore et al. uncovers the biosynthetic gene cluster for the HDAC inhibitor FR-901375 and reveals a novel mechanism enabling modular crosstalk between conserved NRPS and PKS biosynthetic machineries. Using mutagenesis, AlphaFold modeling, and mass spectrometry, the authors show that a conserved β-hairpin docking (βHD) domain directly interacts with the ACP domain, enabling productive noncognate subunit interactions. These findings highlight how nature achieves combinatorial biosynthesis in polyketide–nonribosomal peptide hybrids and suggest an evolutionary pathway for FR-901375 biosynthesis. The work provides new insights into modular enzyme assembly and opens avenues for engineering novel bioactive natural products.
Overall, this is a nice article, well-organized, and the experimental results are comprehensive and thoughtfully interpreted. It would be nice to see some engineering of these pathways but perhaps this could wait for a subsequent publication. I would suggest the following minor comments to be addressed before accepting this manuscript.
1. Some of the figure captions for Fig 1, 2, and 5 are long and too descriptive. Please edit these and make more concise.
2. It is unclear why the terms variable cap and pharmacaphore biosynthetic machinery are used. Please refer to these as the NRPS/peptide portion and PKS portion, respectively.
3. Is largazole the only example of a the drug bound to HDAC. It would be better to use one of the disulfide containing molecules as an example in Fig 2c.
4. In Figure 2, compound 14 refers to largazole, whereas in Figure 4, compound 14 denotes N-hexanoyl-L-valine. It is recommended that the authors adopt distinct number for different compounds to avoid potential confusion.
5. In Figure 4C, what is the rationale for the detection of compound 14 in the Val-Pck (ΔβHD) + hex-PcdC (ΔSLiM) combination? A brief explanation or hypothesis would help clarify this observation.
6. Although the SLiM domain appears to be dispensable for FR-901375 biosynthesis, has its presence or absence been evaluated in terms of its impact on the catalytic efficiency of the enzyme?
7. Is there any experimental evidence supporting a direct physical interaction between PcdK and PcdC (ΔSLiM)? For instance, can PcdC (ΔSLiM) be co-purified with PcdK in a pull-down assay or similar protein-protein interaction assay?
8. The crosstalk experiments are important. I would suggest that a figure be added to the main text to show data for these experiments. If necessary, move the current Fig 6 to supporting information because this is more speculative.
Figure 4b. Is it possible to label PcdC.
Figure 4c. The chromatogram on the bottom shows the presence of 14. Please explain.
Figure 5 title is too long. Make more concise.
Line 216, It is not clear what genes are being referred to.
Line 280, Should be ‘homogeneity’
Line 367, Should be ‘at the C-terminus’

Reviewer #2

(Remarks to the Author)
I co-reviewed this manuscript with one of the reviewers who provided the listed reports. This is part of the Nature Communications initiative to facilitate training in peer review and to provide appropriate recognition for Early Career Researchers who co-review manuscripts.

Reviewer #3

(Remarks to the Author)
Passmore et al. identified the biosynthetic gene cluster of HDAC inhibitor FR-901375 in Pseudomonas chlororaphis subsp. piscium by gene deletion experiments. The comparison between the other gene clusters responsible for the production of the compounds related to FR-901375 clarified the existence of new protein docking modality, in which βHD and the protein region followed with SliMs connects two modules. The way of docking modules and domains were validated by the crosstalk assays, mutagenesis, Alphafold predictions, and carbene footprinting. This is an excellent work based on the high quality biochemical, analytical, genetic, and chemical methodologies. Though the same authors reported that βHD and SliM are important as docking domains between PCP and the terminal C domains (though the deletion of βHD was not accomplished) in Nat. Chem. 2019, 11, 913-923 by the same authors, the impact of this study can be evaluated high, considering the high general interest on the PKS and NRPS which produces bioactive substances. The reviewer recommends the publication after the authors have addressed the points stated below.
1. (Figure 4c) The binding affinities (Kd) between PcdK and (hex-)PcdC, PcdK and (hex-)PcdC (ΔSLiM), PcdK (ΔβHD) and (hex-)PcdC, and PcdK (ΔβHD) and (hex-)PcdC (ΔSLiM) should be compared using ITC or an equivalent method.
2. (Figure 4c) The authors should explain why Val-PcdK(ΔβHD) and hex-PcdC(ΔSLiM) can still generate the product even in the small amount.
3. Does the deletion of βHD affect the multimerization state of PcdK? The authors should show any data on it.
4. The reviewer is interested in the generality of the observation that SLiM is dispensable. The authors should also try to assay the other system which contains βHD and SLiM, including SpiC1 and SpiDE, BhcC and BhcDE, and DepC and DepD, and deletion experiments for βHD and SLiM as shown in Figure 4c, to show the generality of this observation.
5. (Figure 4e) The deletion mutant which lacks SLiM in PcdC should be also constructed, and the production of 13 should be compared.
6. (Figure 5abc) The SLiM should be depicted in the different color.
7. (Line 339) The interaction between βHD and SLiM in Bamb5917/Bamb5915 should be shown in an enlarged view and compared with Figure 5c.
8. (Fig.5d) The conserved residues important for the interaction should be mutated, and their Kd values between PcdC and PcdK or reactions should be assayed.
9. (Line 342)“a network of hydrophobic and polar contacts” should be explained concretely by describing each residue in the text.
10. (Line 386) “phosphanthetheine” should be replaced with “phosphopantetheine”.
11. (Line 604-607) The agarose gel pictures which prove the deletion of the genome should be presented in SI.

Reviewer #4

(Remarks to the Author)
The authors should clearly motivate why they chose to employ accelerated MD in this study to explore the stability of the models. It is more reasonable to run multiple standard MD at room temperature and then carefully perform RMSD analysis on different regions of the system to see how each part is moving within each domain and between domains (within the same protein and across protein interfaces). Judicious clustering could also be useful here. This is all done with the understanding that simply showing stability does not actually prove that a model is stable. For instance, if the simulations are too short it most initial model builds will appear stable.

Extended Data Figure 2 should include RMSD values of the model over time with reference to the initial build at time zero (not the relaxed simulations or some point along the trajectory). This is really needed to explore the stability of the entire complex. Also, it should be done in replicate to determine if the drifts are stochastic or if they are consistent. Also, in panels a and b, the initial model should be shown as ghost with the final snapshot as solid. This will provide information regarding the stability over time from the initial build. Don’t use frame numbers on your time series data, us the actual simulation time in nanoseconds. Would you indicate in panel b what the exact distance is that is being measured in panel c? Perhaps it is best to explore the stability (RMSD compared to the starting configuration) for the licorice elements in panels a/b? The proximity of the atom distance in panel c is potentially interesting, but it is hard to put into context for the overall stability and orientation of this region over time and its distortions from the beginning.

On lines 336-338, why are the authors referring to Supplemental Figure 7? What does this figure support, and how does it relate to Figure 5b? A superposition of an AF model with a solved X-ray structure is shown in the supplement, and the AF prediction is quite good through much of the protein, as it often is. Are the authors suggesting that AF was able to do a good job of predicting the conformation of the previously solved structure? Whatever their goal, they should improve the writing in this section for clarity. Clearly I am missing something here.

Minor comments

When describing the accelerated MD in the Methods, the authors provide the technical terms used in the scripts. Please indicate what each term refers to as well, as this is not common knowledge, even for MD experts.

What barostat and thermostat were used? What was your time step? The Methods section for the MD is lacking some key details.

The authors use the nomenclature of classic MD with the cMD abbreviation on line 745 – this should be defined. Most abbreviate this simply as MD, but we do realize that the Shaw group sometimes uses the abbreviation cMD.

Version 1:

Reviewer comments:

Reviewer #1

(Remarks to the Author)
The revised manuscript have adequately addressed the comments by this reviewer. It is now suitable for publication.

Reviewer #2

(Remarks to the Author)
I co-reviewed this manuscript with one of the reviewers who provided the listed reports. This is part of the Nature Communications initiative to facilitate training in peer review and to provide appropriate recognition for Early Career Researchers who co-review manuscripts.

Reviewer #3

(Remarks to the Author)
In this revision, the authors have adequately addressed most of the concerns raised by this reviewer. Therefore, this reviewer now supports the publication of the current manuscript as it is.

Reviewer #4

(Remarks to the Author)
Overall, the authors have addressed my concerns regarding the MD.

The choice of the Berendsen barostat for the NPT runs was a poor one as it does not reproduce the fluctuations of the true ensemble. The authors should put a note in their manuscript that the fluctuations (RMSDs, distances, etc.) may be artificially suppressed and the stability over estimated from this choice.

A more minor comment is that the potential equation for the aMD in the Methods is missing some parentheses in the denominator.

Open Access This Peer Review File is licensed under a Creative Commons Attribution 4.0 International License, which permits use, sharing, adaptation, distribution and reproduction in any medium or format, as long as you give appropriate credit to the original author(s) and the source, provide a link to the Creative Commons license, and indicate if changes were

made.
In cases where reviewers are anonymous, credit should be given to 'Anonymous Referee' and the source.
The images or other third party material in this Peer Review File are included in the article’s Creative Commons license, unless indicated otherwise in a credit line to the material. If material is not included in the article’s Creative Commons license and your intended use is not permitted by statutory regulation or exceeds the permitted use, you will need to obtain permission directly from the copyright holder.
To view a copy of this license, visit https://creativecommons.org/licenses/by/4.0/

Response to Reviewer Comments

Reviewer 1

1. **Some of the figure captions for Fig 1, 2, and 5 are long and too descriptive. Please edit these and make more concise.**

   Response: Thanks for the suggestion. We have shortened the figure captions accordingly.

2. **It is unclear why the terms variable cap and pharmacophore biosynthetic machinery are used. Please refer to these as the NRPS/peptide portion and PKS portion, respectively.**

   Response: We cannot do this without creating confusion. The conserved pharmacophore machinery is a hybrid NRPS-PKS (NRPS chain initiation and first chain extension module, followed by PKS second and third chain extension module). Similarly, the variable peptidyl cap machinery for the burkholdacs and spiruchostatins is a hybrid NRPS (3 modules)-PKS (1 module). We thought very carefully about this terminology before deciding to use it and we believe it is the clearest way we can describe it.

3. **Is largazole the only example of the drug bound to HDAC. It would be better to use one of the disulfide containing molecules as an example in Fig 2c.**

   Response: Yes, largazole is the only example of this family of compounds for which a crystal structure in complex with an HDAC has been solved.

4. **In Figure 2, compound 14 refers to largazole, whereas in Figure 4, compound 14 denotes N-hexanoyl-L-valine. It is recommended that the authors adopt distinct number for different compounds to avoid potential confusion.**

   Response: We are grateful to the reviewer for highlighting this typographical error. It has been corrected.

5. **In Figure 4C, what is the rationale for the detection of compound 14 in the Val-Pck (ΔβHD) + hex-PcdC (ΔSLiM) combination? A brief explanation or hypothesis would help clarify this observation.**

   Response: We have repeated these assays and trace amounts of product are observed with both the hexanoyl-ACP-SLiM + valinyl-C-A-PCP(ΔβHD) and hexanoyl-ACP(ΔSLiM) + valinyl-C-A-PCP(ΔβHD) combinations. This is likely due to the fact that the ACP and C domains must form a complex for peptide bond formation to occur. Although this is a comparatively weak complex in the absence of the βHD domain, at the high protein concentrations used for the in vitro assays, there is sufficient affinity to support a small amount of product formation. We have clarified this point in the revised manuscript.

6. **Although the SLiM domain appears to be dispensable for FR-901375 biosynthesis, has its presence or absence been evaluated in terms of its impact on the catalytic efficiency of the enzyme?**

   Response: Yes, these data are included in Fig. 6, panel b of the revised manuscript. They show that deletion of the SLiM has a modest effect on the kinetics of amide bond formation, similar to the V101D mutation at the predicted interface between the ACP and βHD domains.

7. **Is there any experimental evidence supporting a direct physical interaction between PcdK and PcdC (ΔSLiM)? For instance, can PcdC (ΔSLiM) be co-purified with PcdK in a pull-down assay or similar protein-protein interaction assay?**

   Response: Unfortunately, the interaction between the PcdC and PcdK subunits is too weak to be detected by a pull down or similar protein-protein interaction assay. The fact that significant amide bond-forming activity is still observed with ΔSLiM constructs (Fig. 4b) but is almost completely abolished with ΔβHD constructs (Fig, 4d) is consistent with a direct physical interaction between the PcdC ACP domain and the PcdK βHD domain. To obtain direct experimental evidence for this, we would need to assess the effect on amide bond formation of a mutation at the predicted ACP-βHD domain interface (e.g. V101D) in the ΔSLiM construct. Such multiple mutagenesis experiments would be interesting to conduct in future research but fall beyond the scope of the present paper.

8. **The crosstalk experiments are important. I would suggest that a figure be added to the main text to show data for these experiments. If necessary, move the current Fig 6 to supporting information because this is more speculative.**

   Response: We agree and have included these data in Fig. 7 of the revised manuscript.

9. **Figure 4b. Is it possible to label PcdC.**

   Response: PcdC does not feature in Fig. 4b. We therefore do not understand what this comment relates to.

10. **Figure 4c. The chromatogram on the bottom shows the presence of 14. Please explain.**

    Response: Please see our response to point 5.

11. **Figure 5 title is too long. Make more concise.**

    Response: Thanks for the suggestion. We have shortened it accordingly.

12. **Line 216, It is not clear what genes are being referred to.**

    Response: These have now been specified.

13. **Line 280, Should be 'homogeneity'**

    Response: We are grateful to the reviewer for spotting this typographical error. It has been corrected.

14. **Line 367, Should be 'at the C-terminus'**

    Response: We are grateful to the reviewer for spotting this typographical error. It has been corrected.

Reviewer 2

1. **Written in conjunction with Reviewer 1**

Reviewer 3

1. **(Figure 4c) The binding affinities (Kd) between PcdK and (hex-)PcdC, PcdK and (hex-)PcdC (ΔSLiM), PcdK (ΔβHD) and (hex-)PcdC, and PcdK (ΔβHD) and (hex-)PcdC (ΔSLiM) should be compared using ITC or an equivalent method.**

   Response: We have attempted to do this using ITC, but unfortunately the binding affinities were too weak to obtain meaningful data.

2. **(Figure 4c) The authors should explain why Val-PcdK(ΔβHD) and hex-PcdC(ΔSLiM) can still generate the product even in the small amount.**

   Response: Please see our response to point 5 of reviewer 1.

3. **Does the deletion of βHD affect the multimerization state of PcdK? The authors should show any data on it.**

   Response: The multimerisation state of the PcdK βHD-C-A-PCP tetra domain and the PcdK C-A-PCP tridomain has been analysed using size exclusion chromatography. The data (included in Supplementary Figure 6 of the revised manuscript) indicate that both proteins are monomers.

4. **The reviewer is interested in the generality of the observation that SLiM is dispensable. The authors should also try to assay the other system which contains βHD and SLiM, including SpiC1 and SpiDE, BhcC and BhcDE, and DepC and DepD, and deletion experiments for βHD and SLiM as shown in Figure 4c, to show the generality of this observation.**

   Response: ΔSLiM mutants of both the DepC and BhcC ACP-SLiM di-domains have been overproduced and assayed. Due to SpiDE being insoluble, we could not test the ΔSLiM mutant of the SpiC1 ACP-SLiM di-domain. Amide bond formation was observed for both constructs with their cognate amino acid-loaded βHD-C-A-PCP tetra-domain partners, indicating that, in general, the

SLiM is not essential for peptide bond formation. These data are included in Supplementary Figures 10 & 11 of the revised manuscript.

We also attempted to construct ΔβHD mutants of the DepD and BhcDE βHD-C-A-PCP tetra-domains. However, due to the very high GC content in this region of the encoding genes, these experiments were unsuccessful.

5. **(Figure 4e) The deletion mutant which lacks SLiM in PcdC should be also constructed, and the production of 13 should be compared.**

   Response: It is not feasible to construct a clean in-frame deletion in the region of *pcdC* encoding the SLiM. The stop codon of *pcdC* overlaps with the start codon of *pcdF*. The SLiM is encoded by a very short region at the 3′ end of *pcdC*. This region forms part of the ribosome binding site for *pcdF*. Deletion of the SLiM-encoding region of *pcdC* would therefore disrupt the expression of *pcdF*, likely abrogating FR-901375 production.

6. **(Figure 5abc) The SLiM should be depicted in the different color.**

   Response: The SLiMs in Figure 5 are depicted in dark blue, which is different from the ACP (light blue), C (yellow) and βHD (red) domains. Each domain has now been labelled in all panels of this figure to provide further clarification.

7. **(Line 339) The interaction between βHD and SLiM in Bamb5917/Bamb5915 should be shown in an enlarged view and compared with Figure 5c.**

   Response: Fig. 5a and 5b show the different overall interaction modalities of PcdK/PcdC and Bamb_5917/Bamb_5915, where the βHD domain interacts with both the SLiM and the ACP domain in the former, but only the SLiM in the latter. Panel c shows a zoomed-in view of the novel proposed interaction epitope between the βHD and ACP domains, which is not observed in the Bamb5917/Bamb5915 system. We therefore do not feel the additional enlarged view suggested by the reviewer is necessary.

8. **(Fig.5d) The conserved residues important for the interaction should be mutated, and their Kd values between PcdC and PcdK or reactions should be assayed.**

   Response: R21K, E97A, V101D, and T102A mutants of the PcdC ACP-SLiM di-domain have been constructed, overproduced, purified, and hexanoyl-phosphopantetheinylated. In assays with the PcdK valinyl-βHD-C-A-PCP tetra-domain, the rate of all amide bond formation was significantly reduced relative to a positive control employing the wild type PcdC ACP-SLiM di-domain. These data have been included in Fig. 6b of the revised manuscript.

9. **(Line 342)“a network of hydrophobic and polar contacts” should be explained concretely by describing each residue in the text.**

Response: The proposed role played by each residue in the network of hydrophobic and polar contacts has been describe in the main text of the revised manuscript.

10. **(Line 386) "phosphanthetheine" should be replaced with "phosphopantetheine".**

    Response: We are grateful to the reviewer for spotting this typographical error. It has been corrected.

11. **(Line 604-607) The agarose gel pictures which prove the deletion of the genome should be presented in SI.**

    Response: These have been included in Supplementary Figure 3 of the revised manuscript.

Reviewer 4

1. **The authors should clearly motivate why they chose to employ accelerated MD in this study to explore the stability of the models. It is more reasonable to run multiple standard MD at room temperature and then carefully perform RMSD analysis on different regions of the system to see how each part is moving within each domain and between domains (within the same protein and across protein interfaces). Judicious clustering could also be useful here. This is all done with the understanding that simply showing stability does not actually prove that a model is stable. For instance, if the simulations are too short it most initial model builds will appear stable.**

   Response: Accelerated MD was carried out in this instance to explore the longer timeframe interactions between the two subunits, and we were particularly interested in the movement of the docking domains with respect to the ACP and C domains. We have now included longer classical MD simulations and RMSD analysis of each domain over the course of the simulation.

2. **Extended Data Figure 2 should include RMSD values of the model over time with reference to the initial build at time zero (not the relaxed simulations or some point along the trajectory). This is really needed to explore the stability of the entire complex. Also, it should be done in replicate to determine if the drifts are stochastic or if they are consistent.**

   Response: Please see our response to point 1 (above). The simulations were also conducted in triplicate, and the same drifts were observed over the course of the 500 ns simulations.

3. **Also, in panels a and b, the initial model should be shown as ghost with the final snapshot as solid. This will provide information regarding the stability over time from the initial build.**

   Response: We thank the reviewer for this helpful suggestion. It has been incorporated into the revised manuscript.

4. **Don't use frame numbers on your time series data, us the actual simulation time in nanoseconds.**

   Response: We thank the reviewer for this suggestion. It has been implemented in the revised manuscript.

5. **Would you indicate in panel b what the exact distance is that is being measured in panel c?**

Response: This has been included in Supplementary Figure 13, panel g.

6. **Perhaps it is best to explore the stability (RMSD compared to the starting configuration) for the licorice elements in panels a/b13?**

   Response: RMSD calculations for both the phosphopantetheinyl-Ser residue and active site His have been included in Supplementary Figure 13, panel f. Similarly to the RMSD for the domains, these residues do not deviate significantly from the starting positions.

7. **The proximity of the atom distance in panel c is potentially interesting, but it is hard to put into context for the overall stability and orientation of this region over time and its distortions from the beginning.**

   Response: Please see our response to point 6 (above).

8. **On lines 336-338, why are the authors referring to Supplemental Figure 7? What does this figure support, and how does it relate to Figure 5b? A superposition of an AF model with a solved X-ray structure is shown in the supplement, and the AF prediction is quite good through much of the protein, as it often is. Are the authors suggesting that AF was able to do a good job of predicting the conformation of the previously solved structure? Whatever their goal, they should improve the writing in this section for clarity. Clearly I am missing something here**.

   Response: We are grateful to the reviewer for raising this point. Additional text has been included in the revised manuscript to clarify this point.

9. **When describing the accelerated MD in the Methods, the authors provide the technical terms used in the scripts. Please indicate what each term refers to as well, as this is not common knowledge, even for MD experts.**

   Response: We are grateful to the reviewer for highlighting this. We have expanded the description of the accelerated MD in the Methods section to include explanations of the terms and how they are used to modify the potential. We have also provided links to sources explaining how these terms are estimated.

10. **What barostat and thermostat were used? What was your time step? The Methods section for the MD is lacking some key details.**

    Response: These points have been clarified in the revised manuscript.

11. **The authors use the nomenclature of classic MD with the cMD abbreviation on line 745 – this should be defined. Most abbreviate this simply as MD, but we do realize that the Shaw group sometimes uses the abbreviation cMD.**

    Response: cMD has been replaced with “Classical MD” to avoid any confusion.
